# Selective C–H Iodination of Weinreb Amides and Benzamides through Iridium Catalysis in Solution and under Mechanochemical Conditions

**DOI:** 10.1021/acs.orglett.3c03190

**Published:** 2023-11-06

**Authors:** Amparo Sanz-Marco, Beatriz Saavedra, Elis Erbing, Jesper Malmberg, Magnus J. Johansson, Belén Martín-Matute

**Affiliations:** † Department of Organic Chemistry, Stockholm University, Stockholm 10691, Sweden; ‡ Medicinal Chemistry, Research and Early Development, Respiratory and Immunology (R&I), Biopharmaceuticals R&D, AstraZeneca, Gothenburg 43183, Sweden; § Medicinal Chemistry, Research and Early Development; Cardiovascular, Renal and Metabolism, Biopharmaceuticals R&D, AstraZeneca, Pepparedsleden 1, Mölndal, 43150 Gothenburg, Sweden

## Abstract

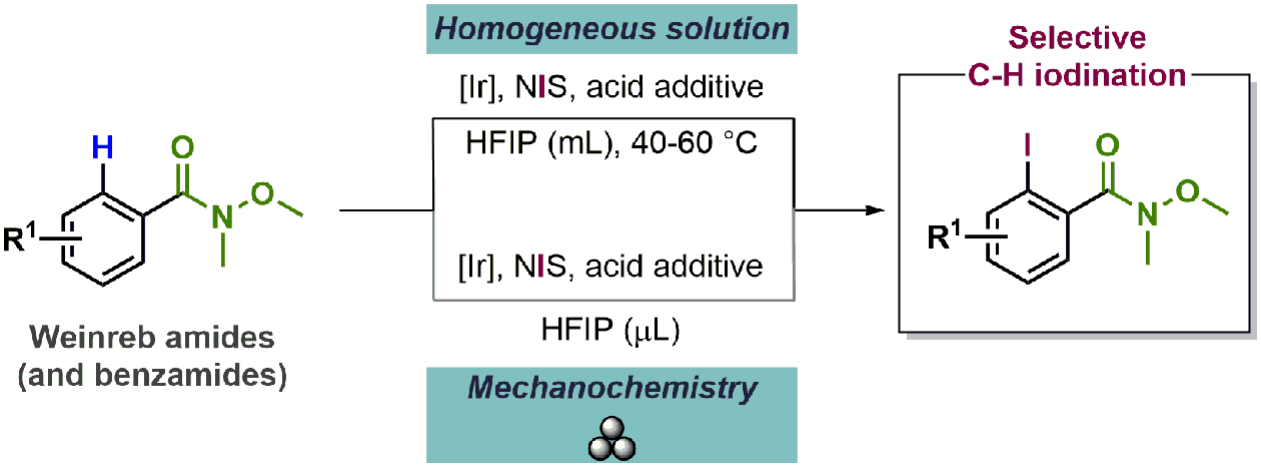

The acid mediated *ortho*-iodination of Weinreb amides using a readily available catalyst is described. The selective *ortho*-iodination of Weinreb amides, challenging substrates in directed C–H activations, and also of benzamides is achieved. The process works under mild conditions and tolerates air and moisture, having a great potential for industrial applications. The methodology can be applied under mechanochemical conditions maintaining the reaction outcome and selectivity.

Aryl halides are unarguably some of the most important building blocks in organic synthesis. They can be further transformed into valuable synthetic intermediates, such as Grignard reagents, and they can react with different nucleophiles in metal-catalyzed cross-coupling reactions.^1^ Furthermore, C–halide bonds are common motifs in compounds with important biological activity. Introduction of the halide functionality through a directed C–H activation methodology is an efficient and straightforward way to synthesize halogenated and other functionalized scaffolds.^2−5^

Amides are common and important functional groups in organic synthesis and have been reported to be efficient directing groups in transition-metal-catalyzed C–H activation/functionalization procedures (Scheme 1).^6^ In particular, Glorius and co-workers^7^ reported a first comprehensive study on their C–H halogenation of aromatic amides and ketones using commercially available Rh(III)-catalyst and a silver salt as co-catalyst in dichloroethane. Their work was later expanded to electron rich heterocycles.^8^ These pioneering results have been followed by others employing other catalytic systems based on cobalt,^9^ ruthenium,^10^ palladium,^11^ and other metals.^12^

**Scheme 1:**
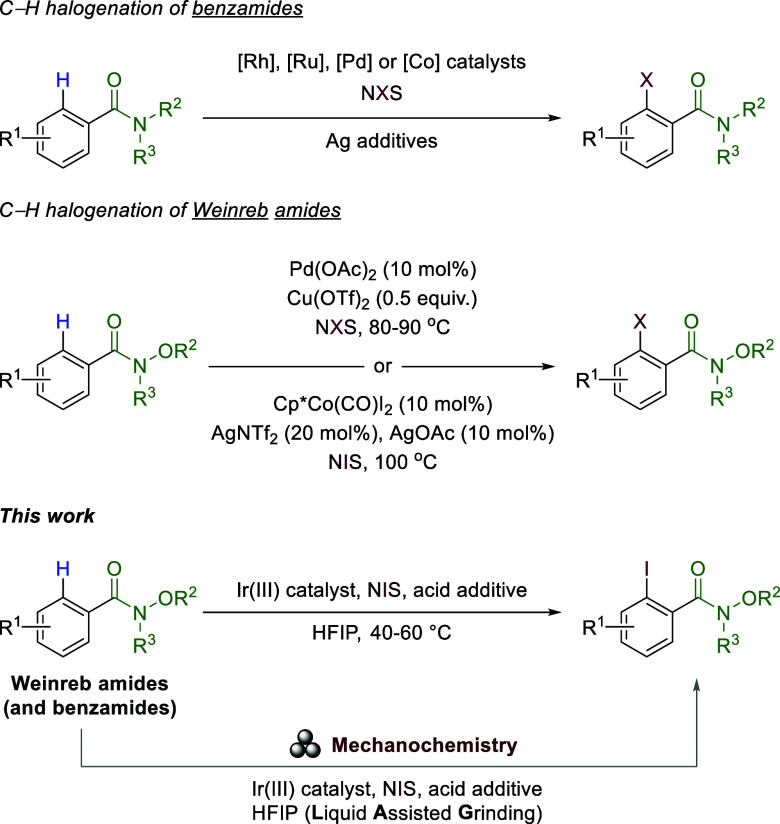
C–H Activation/Iodination of Substrates with Weakly Coordinating Directing Groups and This Work

Among the amides, Weinreb amides (*N*-methyl-*N*-methoxyamides) are very important functional groups from a synthetic point of view, due to their unique reactivity.^13^ Weinreb amides are challenging substrates in C–H activation reactions due to their weak coordination properties^14^ but have been quite thoroughly studied in combination with C–C bond-forming reactions.^15^ In 2017, Das and Kapur^16^ presented the first C–H activation/halogenation of Weinreb amides as the directing group, opening up a new route to this versatile class of molecules. The reaction required 10 mol % of a Pd(II) salt and 0.5 equiv of a Cu(II) co-catalyst. Shortly after, Matsunaga^17^ and co-workers also reported the ability of Weinreb amides to undergo C–C and C–I bond-forming reaction through *ortho-*C–H activation using Cp*Co(III) catalysis, highlighting the importance of this unexplored functionality for C–H activation processes. Despite the use of a Co catalyst, high catalyst loading was needed (10 mol %), in addition to 30 mol % of a mixture of two silver salts and high temperatures (100 °C). More recently, a palladium-catalyzed C–H iodination of phenylacetic acid Weinreb amides was published.^18^ Overall, the existing protocols for the use of the Weinreb amide as the directing group in C–H activations are somehow limited for industrial applications due to high catalyst loadings, harsh conditions, and the need for a significant number of additives.

Our group previously published an Ir(III)-catalyzed method to synthesize *ortho*-iodobenzoic acids under very mild and additive-free conditions through C–H activation using only NIS in 1,1,1,3,3,3-hexafluoroisopropanol (HFIP) at 40 °C.^4^ It was found that HFIP^19^ had a pivotal role in the reaction mechanism, as it lowered the activation energy of the reductive elimination step, in addition to being an excellent solvent for the reaction. Both the carboxylic acid and amide moieties are deactivating functional groups in electrophilic aromatic substitution reactions. In the absence of a catalyst, they direct the substitution reaction toward the *meta*-C–H bond. Later on, we also reported a selective *ortho-*monoiodination of benzoic acids with two unbiassed C–H bonds.^5^

Herein, the Ir(III)-catalyzed C–H iodination of different amides, and in particular of Weinreb, is reported.^20^ In addition to an acid additive, no additional reagents, such as oxidants, are required. Moreover, the method can be applied using liquid-assisted grinding (LAG) in a mechanochemical setup, requiring shorter reaction times and, more importantly, maintaining the reaction outcome and selectivity.

We started our investigations by applying [Cp*Ir(H_2_O)_3_]SO_4_ (3 mol %) as the catalyst and 1.5 equiv of NIS in HFIP at 40 °C to the C–H iodination of *N*-*tert*-butylbenzamide **1a**, as those previously employed for benzoic acids^4^ (i.e., Table 1, entry 1). After overnight stirring, only a moderate conversion into **2a** was obtained. Increasing the temperature to 50 or 60 °C did not result in a better yield (entries 2 and 3). Mechanistic investigations performed on the iodination of benzoic acids^4^ revealed that the resting state was an Ir(III) complex with the iodinated benzoate product as a ligand. The final *ortho-*iodobenzoic acid was formed upon protonation of this resting state intermediate with another molecule of benzoic acid. If one assumes a similar scenario for the C–H iodination of benzamides, breaking a related amidate–iridium complex through protonation might be more difficult due to the significantly lower acidity of benzamides compared to benzoic acids. We therefore investigated the effect of different acid additives on the reaction outcome (entries 4–7). Confirming this hypothesis, the addition of just 1 equiv of acetic acid provided quantitative conversion of **1a**. The beneficial effect of the acid additive is so significant that the reaction does not stop until essentially full consumption of NIS, which results in a mixture of mono- and diiodinated benzamide in a ratio of **2a**/**3a** = 60/40 (entry 4). On the other hand, *p*-TsOH inhibited the catalytic activity (entry 5). PivOH gave >95% conversion and better selectivity toward the **2a** (entry 6). Trifluoroacetic acid (TFA) afforded quantitative conversion and excellent yields of **2a** and **3a** with a similar selectivity in favor of the monoiodination product (**2a**/**3a** = 78/22, entry 7). Further investigations on the number of equivalents of TFA or PivOH (entries 8–14) revealed that 0.3 equiv of TFA yielded the highest selectivity (**2a**/**3a** = 85/15, entry 11) and in quantitative yield. Molecular iodine was also tested as an iodinating agent with no success (entry 15).

**Table 1:**
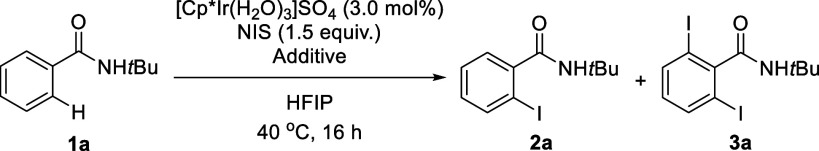
Screening of Acidic Additives for the C–H Activation/Iodination of Benzamides^a^

Entry	Additive (equiv)	*T* (°C)	Conv. (%)	Yield **2a**/**3a** (%)^b^
1		40	51	51/0
2		50	45	45/0
3		60	44	44/0
4	AcOH (1)	40	>99	60/40
5	*p*-TsOH^c^ (1)	40	0	0
6	PivOH (1)	40	>95	76/19
7	TFA (1)	40	>99	78/22
8	TFA (2)	40	>99	77/23
9	TFA (0.5)	40	>99	80/20
10	TFA (0.4)	40	>99	78/22
11	TFA (0.3)	40	>99	85/15
12	PivOH (0.3)	40	>85	69/17
13	TFA (0.2)	40	>99	83/17
14	TFA (0.1)	40	>99	74/26
15^d^	TFA (0.5)	40	0	0

a **1a** (0.1 mmol), NIS (1.5 equiv), [Cp*Ir(H_2_O)_3_]SO_4_ (3.0 mol %), and additive in HFIP (1.0 mL, [**1a**] = 0.1 M).

b Yield by ^1^H NMR spectroscopy using an internal standard.

c *p*-TsOH·H_2_O.

d Using I_2_ as the iodinating agent.

The capacity to enable solvent-free/solvent-less reactions is especially significant from a sustainability point of view. In this context, mechanochemical reactions have emerged as a powerful strategy for developing more sustainable synthetic methods.^21^ Mechanochemical processes can occur without solvents or in the presence of small amounts of solvent, i.e., liquid-assisted grinding (LAG).^22^ The C–H activation using mechanochemistry^23^ may have some potential advantages in this work, such as diminished consumption of HFIP, milder reaction conditions (no heating applied), and shorter reaction times. After screening the amount of iridium catalyst, the equivalents of NIS, and the frequency for the reaction, we found [Cp*Ir(H_2_O)_3_]SO_4_ (5 mol %), NIS (1.5 equiv), and 20 Hz to be the best conditions (Table 2). Different acid additives (entries 1–3) were also tested, and the best result was obtained using PivOH (entry 3). A better reaction outcome in terms of yield and selectivity (**2a**/**3a** = 79/11, entry 4) was achieved in a smaller stainless steel jar. Furthermore, adding two smaller grinding balls (5 ϕ mm) instead of just one (10 ϕ mm) gave slightly better selectivity. The amount of solvent was also evaluated (entries 5–7), obtaining the best result with 200 μL of HFIP (LAG = 0.76; [**1**] = 1.25 M, entry 7). Note that the *ortho-*iodination reaction without the Ir catalyst or without both Ir and PivOH did not take place.

**Table 2:**
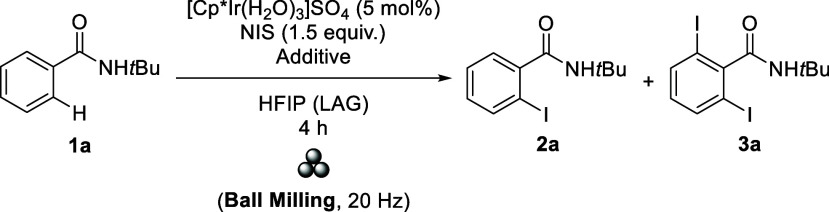
Optimization for the C–H Activation/Iodination of Benzamides Using Mechanochemistry^a^

Entry	Additive (5 equiv)	HFIP (μL)	Conv. (%)	Yield **2a**/**3a** (%)^b^
1	TFA	250	25	25/0^c^
2	AcOH	250	>55	51/3^c^
3	PivOH	250	>75	68/10^c^
4	PivOH	250	>90	79/11^d^
5	PivOH	250	>90	80/10^e^
6	PivOH	150	>55	50/3^e^
7	PivOH	200	>90	81/11^e^

a **1** (0.25 mmol), NIS (1.5 equiv), [Cp*Ir(H_2_O)_3_]SO_4_ (5.0 mol %), and acid (5 equiv) in HFIP (LAG).

b Yield by ^1^H NMR spectroscopy using an internal standard.

c 10 mL stainless steel jar and 1 grinding ball (10 ϕ mm).

d 5 mL stainless steel jar and 1 grinding ball (10 ϕ mm).

e 5 mL stainless steel jar and 2 grinding balls (5 ϕ mm).

The reaction scope was evaluated by testing *sec-* and *tert-*benzamides (Table 3) under the optimized reaction conditions in solution (Table 1, entry 11). Common *sec-*amide functionalities on the phenyl, such as NH*i*Pr (**1c**), NHEt (**1d**), and NHBn (**1e**), afforded excellent conversion into the iodinated products **2** and **3**. *Tertiary* amides also afforded excellent yields (**2f**, **2g**). Primary amides, however, gave very low conversions. Importantly, amides **1f**–**1h** reacted, giving the corresponding C–H iodination products with excellent selectivity. The method could expand to C–H bromination of **1a** giving product **2b** in good yield and selectivity. Under the best mechanochemical conditions from Table 2 (entry 7), *N*-(*tert*-butyl)benzamide (**1a**) and *N*-isopropylbenzamide (**1c**) gave isolated yields similar to that obtained under solution (70% yield and 71% yield, respectively). When *N*-benzylbenzamide (**1e**) was used, a higher yield and selectivity was observed. *N*,*N*-Dimethyl benzamide (**1g**) also gave a similar yield when mechanochemical conditions were applied (76% yield). Unfortunately, no better results were obtained when benzamide (**1h**) was used as the substrate (19% yield of **2h**). Note that, when the reaction was performed under mechanochemical conditions, shorter reaction times were needed (4 h versus 16 h), obtaining similar or even better yields and selectivities.

**Table 3:**
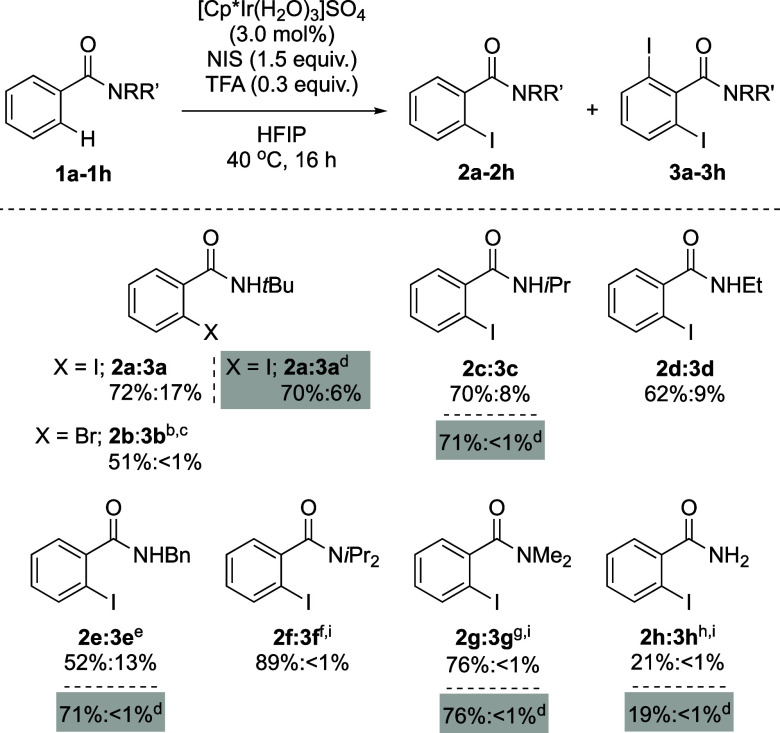
C–H Iodination of Benzamides^a^

a **1** (0.2 mmol), NIS (1.5 equiv), [Cp*Ir(H_2_O)_3_]SO_4_ (3.0 mol %), and TFA (0.3 equiv) in HFIP (2.0 mL, [**1**] = 0.1 M). Isolated yields unless otherwise noted. Unless noted, full conversion.

b NBS (2 equiv) and TFA (1 equiv).

c 42% of **1a**.

d Mechanochemical conditions: **1** (0.25 mmol), NIS (1.5 equiv), [Cp*Ir(H_2_O)_3_]SO_4_ (5.0 mol %), and PivOH (5 equiv) in HFIP (200 μL – η = 0.76; [**1**] = 1.25 M), 5 mL jar, 2 balls (ϕ = 5 mm) and 20 Hz for 4 h.

e 22% of **1e**.

f 60 °C.

g 24% of **1g**.

h Determined by ^1^H NMR spectroscopy.

i 6 h reaction time and portionwise addition of NIS (Supporting Information).

We turned our attention to the more challenging Weinreb amides. After a brief screening of the reaction conditions on *N*-methoxy-*N*-methylbenzamide (**4a**) as the substrate (Table S1), it was established that the best results were obtained with 0.5 equiv of TFA at 60 °C. Remarkably, complete selectivity toward monoiodination products (**5**) was obtained for all Weinreb amides tested. A robustness test based on the method provided by Collins and Glorius^24^ was performed. It was concluded that ketones are well tolerated (Table 4, entry 1) but aldehydes inhibit the catalysis as well as decompose under the reaction conditions (entry 2).

**Table 4:**
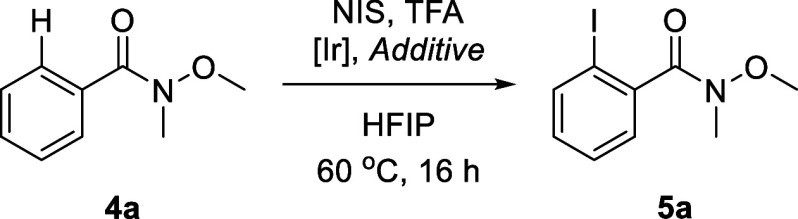

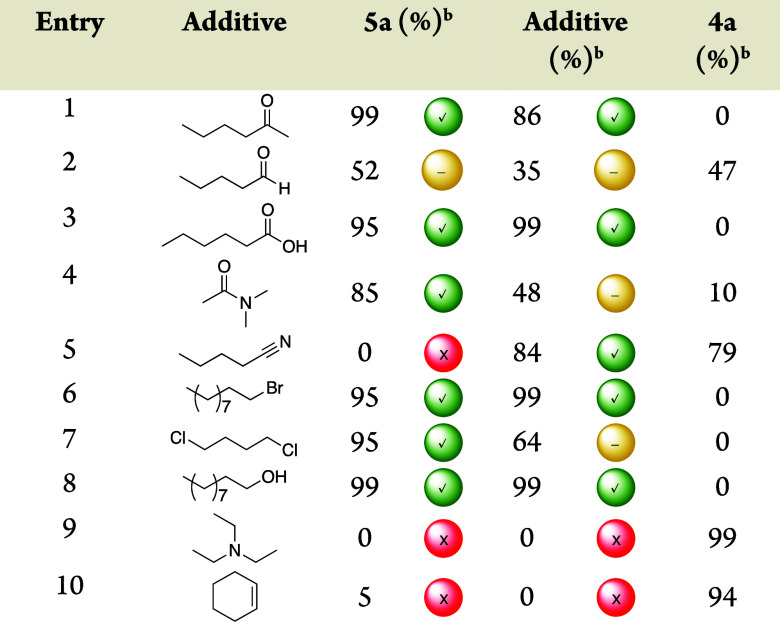
Robustness Test^a^

a **4a** (0.25 mmol), NIS (1.5 equiv), [Cp*Ir(H_2_O)_3_]SO_4_ (3.0 mol %), TFA (0.5 equiv), and additive (1 equiv) in HFIP (2.5 mL, [**4a**] = 0.1 M).

b Measured by ^1^H NMR using an IS.

Aliphatic carboxylic acids are well tolerated (entry 3), and aliphatic amides are only moderately compatible (entry 4). The nitrile functional group (entry 5) inhibited the catalysis completely. Other functional groups such as aliphatic bromides, chlorides, and alcohols (entries 6–8) are essentially compatible with the reaction conditions. Tertiary amines, however, inhibit the reaction (entry 9), and unsaturated compounds are not tolerated plausibly due to their inherent reactivity with NIS under these conditions (entry 10). The same conclusion was reached when an aliphatic bromide (entry 6, compatible) and Et_3_N (entry 9, not tolerated) were used under mechanochemical conditions.

With the robustness test results in hand, a number of Weinreb amides were subjected to the reaction conditions (Table 5). To avoid decomposition of NIS under the reaction conditions (see the Supporting Information), it was added in 3 portions every 2 h, giving a total reaction time of 6 h. Amide **4b**, with an *ortho-*F substituent, afforded **5b** in a 44% yield. As proposed by Matsunaga,^17^ the lack of reactivity or the smaller yields obtained for these substrates are likely due to the steric repulsion between the *ortho*-substituent and the Weinreb amide moiety, hindering the formation of the metallacycle intermediate.

**Table 5:**
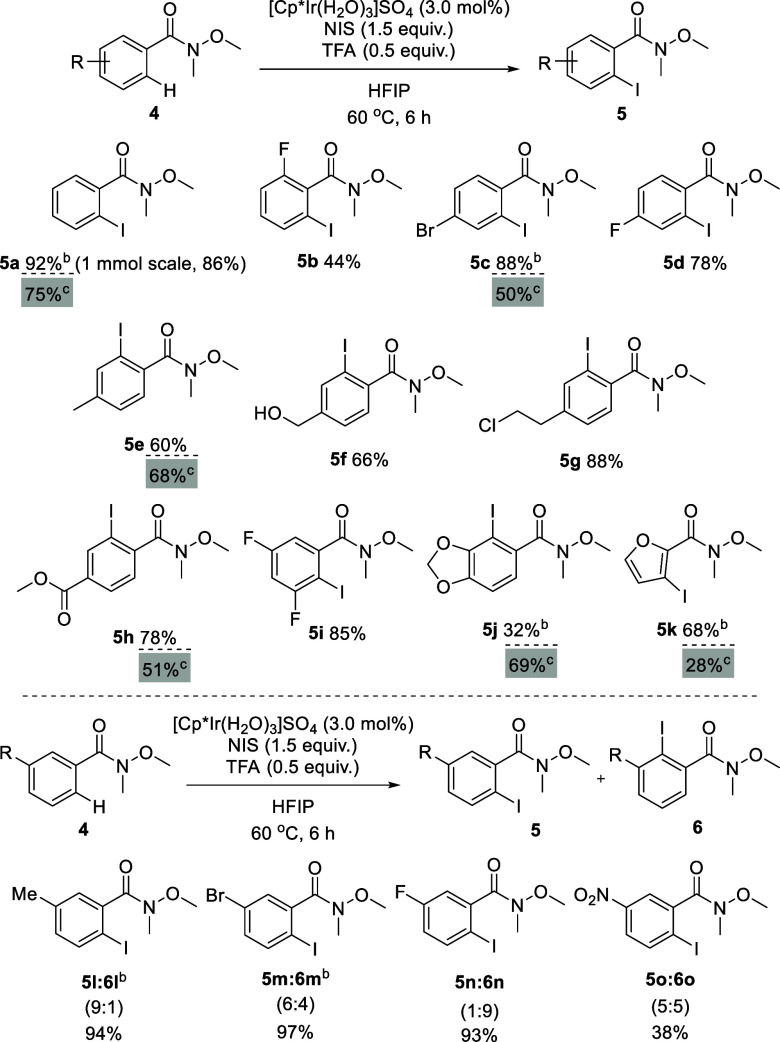
Scope of Weinreb Amides^a^

a **4** (0.25 mmol), NIS (1.5 equiv), [Cp*Ir(H_2_O)_3_]SO_4_ (3.0 mol %), and TFA (0.5 equiv) in HFIP (2.5 mL, [**4**] = 0.1 M). Isolated yields. The remainder was SM unless otherwise noted.

b No SM was detected.

c Mechanochemical conditions: **4** (0.25 mmol), NIS (1.5 equiv), [Cp*Ir(H_2_O)_3_]SO_4_ (5.0 mol %), and PivOH (5 equiv) in HFIP (200 μL – η = 0.77; [**4**] = 1.25 M), 5 mL jar, 2 balls (ϕ = 5 mm) and 20 Hz for 4 h.

These observations fit well with the high selectivity toward monoiodination of the parent Weinreb amide **4a** (i.e., the diiodinated species are not formed). At the *para*-position, bromo (**4c**), fluor (**4d**), methyl (**4e**), benzyl alcohol (**4f**), aliphatic group bearing a chloro moiety (**4g**), and methyl ester (**4h**) substituents afforded the respective products in good yield ranging from 60% to 88%, in agreement with the robustness test. Highly functionalized Weinreb amides (**4i**, **4j**) provided the desired product in a good to moderate yield. The Weinreb amide derived from furanoic acid (**4k**) afforded product **5k** in a good yield of 68%. Methyl (**4l**), bromo (**4m**), and fluor (**4n**) substituents at the *meta*-carbon were well tolerated. When a nitro group is present in the *meta*-position, a moderate conversion was obtained yielding products **5o** and **6o** (38% combined yield).^16^ Additionally, *N*-methoxy-*N*-methylbenzamide (**4a**) was successfully monoiodinated using mechanochemical reaction conditions giving a 75% yield and exclusively the *ortho*-iodinated product. A slightly better yield was obtained for **5e** (60% in solution versus 68% under mechanochemical reaction conditions). This methodology gave lower yields for compounds **5c**, **5h**, and **5k**, but surprisingly **5j** gave 69% yield.

A KIE of 1.96 ± 0.11 was obtained when comparing the rate of **1a** to that of **1a**-*d*_2_ (under noncompetition conditions). Moreover, a KIE of 2.77 ± 0.38 was observed when **4a** and **4a**-*d*_2_ were used (see the Supporting Information).

In conclusion, an Ir-catalyzed *ortho-*C–H functionalization of aromatics having weakly coordinating Weinreb amides was developed. The method can also be applied to benzamides. A key aspect to increase the turnover of the catalyst is the use of acid additives. We have also shown that the iodination can be performed mechanochemically, minimizing the use of HFIP. This setup requires shorter times than when the solvent conditions are used and do not require consecutive additions of NIS, giving similar or even higher yields and selectivities. The methods herein presented are very well suited for SAR studies of small molecules in drug discovery settings. It enables diversification in a single step and is complementary to electrophilic aromatic substitutions, which would be directed to the *meta-*C–H bond for these substrates. The iodine allows for a plethora of further downstream functionalizations. In early drug discovery, where access to close analogues of lead compounds is of ultimate importance, C–H functionalization offers disconnections with overall lower step count compared to more traditional methodologies. Despite using iridium catalysis, the very high functional group tolerance makes this method an excellent tool in early drug discovery, providing drug analogues with a reduced carbon footprint due to the step economy gained in C–H activation.

## Data Availability

The data underlying this study are available in the published article and its Supporting Information.

## Supporting Information Available

Screening of conditions for Weinreb amides and stability studies of NIS under the reaction conditions; experimental procedures; and characterization and NMR spectra of products (PDF)
