# Supplementary material for: Selective C–H Iodination of Weinreb Amides and Benzamides through Iridium Catalysis in Solution and under Mechanochemical Conditions
Source: Org Lett. 2023 Nov 6;26(14):2800–5. doi: 10.1021/acs.orglett.3c03190 (PMC11019638; doi:10.1021/acs.orglett.3c03190)

Supporting Information

Selective C–H Iodination of Weinreb- and Benzamides through Iridium Catalysis in Solution and under Mechanochemical Conditions

Amparo Sanz-Marco,[a,‡] Beatriz Saavedra,[a,‡] Elis Erbing,[a] Jesper Malmberg,[b] Magnus J. Johansson,[c] Belén Martín-Matute[a,*]

[a] Department of Organic Chemistry, Stockholm University, Stockholm 10691, Sweden.

[b] Medicinal Chemistry, Research and Early Development, Respiratory and Immunology (R&I), Biopharmaceuticals R&D, AstraZeneca, Gothenburg 43183, Sweden.

[c] Medicinal Chemistry, Research and Early Development; Cardiovascular, Renal and Metabolism, Biopharmaceuticals R&D, AstraZeneca, Pepparedsleden 1, Mölndal, 431 50 Gothenburg.

belen.martin.matute@su.se

Table of contents

S2 General information

S3 Optimization studies

S5 General procedure for the synthesis of *ortho*-halogenated products

S6. Mechanochemical procedure for the synthesis of *ortho*-halogenated products

S6 Synthesis and characterization data for *ortho*-halogenated benzamides **2**

S10 Characterization data for *ortho*-diiodobenzamides **3**

S12 Synthesis and characterization data for *ortho*-iodo-*N*-methoxy-*N*-methylbenzamides **5** and **6**

S22 Synthesis and characterization data for deuterated benzamides **1a**-d_2 and **4a**-d_2

S23 Synthesis and characterization data for deuterated *ortho*-iodo-benzamides **2a**-*d* and **5a**-*d*

S25 Kinetic Isotope Effect

S26 References

S27 ^{1}H NMR and ^{13}C NMR of *ortho*-halogenated products **2**, **3**, **5** and **6**

S56 ^{1}H NMR, ^{13}C NMR of deuterated benzamides **1a**-d_2 and **4a**-d_2

S58 ^{1}H NMR, ^{13}C NMR of deuterated *ortho*-iodo-benzamides **2a**-d_1 and **5a**-d_1

General information

All reagents were used as obtained from commercial sources without further purification. Flash chromatography was performed on a Biotage® Isolera™ One, automated flash system, with SNAP KP-Sil or SNAP Ultra cartridges, using a step- or gradient eluent, detection by UV 200-400 nm using mixtures pentane / acetone as eluent or with 60 Å (35-70 μm) silica gel (GC 60A 35-70 Micron, DAVISIL) using mixtures petroleum ether / EtOAc as eluent. Analytical TLC was performed on aluminum plates pre-coated with silica gel (Merck, Silica Gel 60 F254). Compounds were detected by exposure to UV light or by revealing the plates in a solution of 5% $KMnO_4$ in water. Melting points were recorded in metal block and are uncorrected. ^1H and ^{13}C NMR spectra were recorded at 400 or 500 MHz and 100 or 125 MHz respectively on Bruker Advance spectrometers. Chemical shifts (δ) are shown in ppm, using the residual peaks of $CH(D)Cl_3$ (δ_H 7.26 and δ_C 77.00) as reference. Coupling constants (J) are given in Hz. NMR yields were calculated using 1 equiv. of 1,2,4,5-tetrachloro-3-nitrobenzene as internal standard. High-resolution mass spectra (HRMS) were recorded on Bruker *micro*TOF ESI-TOF mass spectrometer. Benzamides **1a-1e**,[1] **1g**[2] were synthetized following procedures reported in the literature. Benzamides **1f** and **1h** are were purchased from Aldrich Chemical Co. and used without further purification. Weinreb amide **4f** was synthesized following a literature procedure.[3] All other Weinreb amides **4**, were synthesized from the corresponding acyl chlorides or carboxylic acids using *N,O*-dimethylhydroxylamine hydrochloride.[4] The catalyst, $[Cp^*Ir(H_2O)_3]SO_4$, was synthesized following a literature procedure.[5] Mechanochemical reactions were performed using a Restch Mixer Mill MM 500 Vario, stainless steel screw-top grinding jars of 5-10 mL and stainless steel grinding jars ϕ = 5-10 mm.

Optimization Studies

Table S1. Screening of additives for C-H activation / iodination employing Weinreb amides as the directing group.[a]

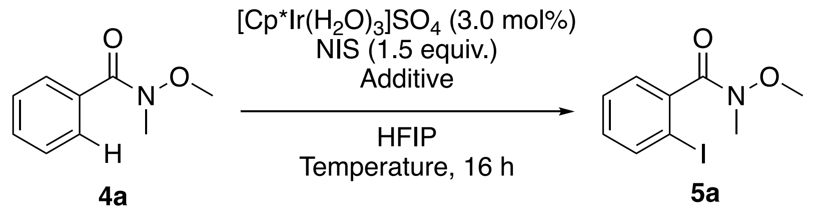

Entry	Additive (equiv.)	Temp (°C)	Conversion (%)	Yield (%)[b]
1	-	RT	24	22
2	-	40	24	20
3	-	60	30	21
4	AcOH (1)	60	>99	90
5	TsOH[c] (1)	60	0	0
6	TFA (1)	60	>99	97
7	TFA (1)	40	>99	99
8	TFA (2)	40	66	66
9	TFA (5)	60	25	0
10	TFA (2.5)	60	47	18
11	TFA (2)	60	>99	92
12	TFA (0.75)	60	>99	94
13	TFA (0.5)	60	>99	94
14[d]	TFA (0.5)	40	0	0

[a]**4a** (0.25 mmol), NIS (0.375 mmol), $[Cp^*Ir(H_2O)_3]SO_4$ (3.0 mol%, 0.0075 mmol) and additive in HFIP (2.5 mL, [**4a**] = 0.1M). [b]Yield measured by ^1H NMR using 1,2,4,5-tetrachloro-3-nitrobenzene as an internal standard. [c]*p*-TsOH•H_2O [d]Using I_2 instead of NIS.

Stability investigation of NIS under the reaction conditions

During the investigation of the conditions, formation of molecular iodine was observed. This is unwanted, as I_2 is not an active iodinating agent under these conditions. Therefore, we were interested in knowing the stability of NIS under our conditions. To investigate this, the formation of I_2 was studied for 2 hours, in only HFIP, with the Ir-complex, TFA and the mixture of both (Figure S1). It could be concluded that the 3formation of molecular iodine is fast when the catalyst is present, and slightly increased by the presence of TFA. These experiments were performed by stirring the NIS (0.375 mmol, 84 mg) for 2 h in HFIP (2.5 mL) (**1**), adding TFA (0.0125 mmol, 10 µL) (**2**), $[Cp^*Ir(H_2O)_3]SO_4$ (0.0075 mmol, 3.5 mg) (**3**) and both TFA and the Iridium catalyst (**4**), at room temperature and 60 °C.

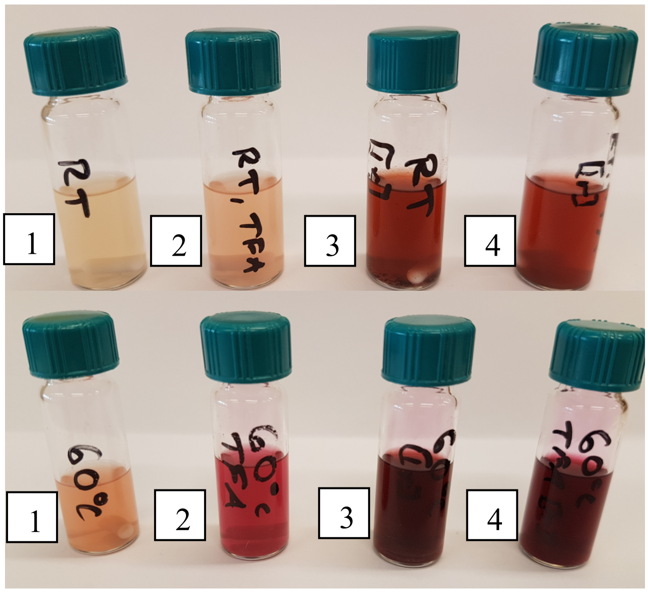

Figure S1: (In)stability of NIS under the reaction conditions. Top: At room temperature. Bottom: At 60 ºC. From the left: 1) Only HFIP. 2) HFIP +TFA. 3) HFIP + [Ir]. 4) HFIP + TFA + [Ir].

General procedure for the synthesis of *ortho*-halogenated products

Method A:

Amide **1** (0.2 mmol), [Cp*Ir(H_2O)$_3$]SO_4 (3.0 mol%, 0.006 mmol, 3 mg), *N*-iodosuccinimide (0.3 mmol, 68 mg) or *N*-bromosuccinimide (0.4 mmol, 72 mg) and trifluoroacetic acid (0.06 mmol, 5 μL) were dissolved in 1,1,1,3,3,3-hexafluoro-2-propanol (HFIP, 0.1 M, 2.0 mL) under air atmosphere in a capped vial and stirred at 40 °C (oil bath) for 16 hours. The reaction was quenched by addition of $Na_2S_2O_3$ (sat.) (2 mL). The aqueous phase was extracted with CH_2Cl_2 (3 x 20 mL), and the combined organic phases were washed with brine (10 mL), dried over $MgSO_4$, and concentrated under reduced pressure. The product was purified by column chromatography (petroleum ether / ethyl acetate) providing the *ortho*-iodobenzamide **2**.

Method B:

The substrate (0.25 mmol) and [Cp*Ir(H_2O)$_3$]SO_4 (3.0 mol%, 7.5 μmol, 3.5 mg) was dissolved in HFIP (0.1 M, 2.5 mL) under air atmosphere in a capped vial and trifluoroacetic acid (0.125 mmol, 10 μL) was added. When the reagents were completely dissolved *N*-iodosuccinimide (0.375 mmol, 84 mg) was added in three portions waiting 2 hours between additions and stirring the reaction at 60 °C (oil bath). After 6 hours the reaction was quenched by the addition of $Na_2S_2O_3$ (sat. 0.5 mL) and water (20 mL), extracted with CH_2Cl_2 (25 mL), dried with $MgSO_4$, filtered and evaporated. The product was purified by column chromatography (petroleum ether / ethyl acetate) providing the *ortho*-iodobenzamide **4**, **5** or **6**.

1 mmol scale reaction: Weinreb amide **4a** (1 mmol, 165 mg) and [Cp*Ir(H_2O)$_3$]SO_4 (3.0 mol%, 0.03 mmol, 14 mg) was dissolved in HFIP (0.1 M, 10 mL) under air atmosphere in a capped vial and trifluoroacetic acid (0.5 mmol, 38 μL) was added. When the reagents were completely dissolved *N*-iodosuccinimide (1.5 mmol, 337 mg) was added in three portions waiting 2 hours between additions and stirring the reaction at 60 °C (oil bath). After 6 hours the reaction was quenched by the addition of $Na_2S_2O_3$ (sat. 20 mL) and water (20 mL), extracted with CH_2Cl_2 (3 x 40 mL), dried with $MgSO_4$, filtered and evaporated. The product was purified by column chromatography (pentane / acetone, 90:10) providing **5a** (251.7 mg, 86%).

Mechanochemical procedure for the synthesis of *ortho*-halogenated products

Amide **1** or **4** (0.25 mmol), $[Cp^*Ir(H_2O)_3]SO_4$ (5.0 mol%, 0.0125 mmol, 6 mg) and pivalic acid (1.25 mmol, 128 mg) were dissolved in HFIP (1.25 M, 200 µL) under air atmosphere in a stainless steel grinding jar (5 mL) equipped with 2 stainless steel grinding ball (ϕ = 5 mm). Then, *N*-iodosuccinimide (0.375 mmol, 84 mg) was added and the mixture was mixed (20 Hz) for 4 hours (no heating). The reaction was quenched by addition of $Na_2S_2O_3$ (sat.) (2 mL). The aqueous phase was extracted with CH_2Cl_2 (3 x 10 mL), and the combined organic phases were washed with brine (10 mL), dried over $MgSO_4$, and concentrated under reduced pressure. The product was purified by column chromatography providing the *ortho*-iodobenzamide **2** or **5**.

Synthesis and characterization data for *ortho*-halogenated benzamides 2

_N_-(_tert_-Butyl)-2-iodobenzamide (2a)

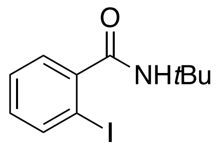

The title compound was prepared according the method A from *N*-(*tert*-butyl)benzamide (**1a**, 35.5 mg). Purification by column chromatography (SiO_2; petroleum ether / ethyl acetate, 90:10) afforded **2a** as a colorless solid (43.6 mg, 72%).

^{1}H NMR (400 MHz, $CDCl_3$) δ = 7.83 (ddd, *J* = 8.0, 1.1, 0.5 Hz, 1H), 7.39–7.33 (m, 2H), 7.06 (ddd, *J* = 8.0, 6.9, 2.3 Hz, 1H), 5.73 (bs, 1H), 1.49 (s, 9H) ppm. ^{13}C NMR (100 MHz, $CDCl_3$) δ = 168.7, 143.3, 139.7, 130.7, 128.14, 128.12, 92.4, 52.2, 28.7 ppm. HRMS-ESI: Calculated for $C_{11}H_{14}NOINa$ $[M+Na]^+$ = 326.0012 m/z. Found: 326.0045 m/z.

The spectral properties of **2a** is in good agreement with literature data.[6]

2-Bromo-*N*-(*tert*-butyl)benzamide (2b)

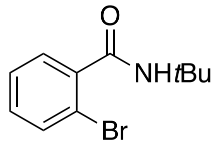

The title compound was prepared according the method A from *N*-(*tert*-butyl)benzamide (**1a**, 35.5 mg), and trifluoroacetic acid (0.4 mmol, 32 μL). Purification by column chromatography (Pentane:Acetone - 9:1) afforded **2b** as a colorless solid (26.1 mg, 51%).

^{1}H NMR (400 MHz, $CDCl_3$) δ = 7.55 (dd, *J* = 8.1, 1.1 Hz, 1H), 7.48 (dd, *J* = 7.6, 1.8 Hz, 1H), 7.33 (td, *J* = 7.6, 1.2 Hz, 1H), 7.23 (ddd, *J* = 8.1, 7.4, 1.8 Hz, 1H), 5.73 (bs, 1H), 1.47 (s, 9H) ppm. ^{13}C NMR (100 MHz, $CDCl_3$) δ = 166.9, 139.0, 133.2, 130.8, 129.3, 127.5, 119.1, 52.2, 28.7 ppm. HRMS-ESI: Calculated for $C_{11}H_{14}NO^{79}BrNa$ $[M+Na]^+$ = 278.0151 m/z. Found: 278.0150 m/z.

The spectral properties of **2b** is in good agreement with literature data.[7]

2-Iodo-*N*-isopropylbenzamide (2c)

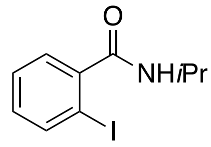

The title compound was prepared according the method A from *N*-isopropylbenzamide (**1c**, 32.6 mg). Purification by column chromatography (SiO_2; petroleum ether / ethyl acetate, 90:10) afforded **2c** as a colorless solid (40.5 mg, 70%).

^{1}H NMR (400 MHz, $CDCl_3$) δ = 7.85–7.82 (m, 1H), 7.38–7.33 (m, 2H), 7.09–7.05 (m, 1H), 5.59 (bs, 1H), 4.31–4.26 (m, 1H), 1.28 (d, *J* = 6.6 Hz, 6H) ppm. ^{13}C NMR (100 MHz, $CDCl_3$) δ = 168.5, 142.5, 139.7, 130.9, 128.2, 128.1, 92.4, 42.2, 22.6 ppm. HRMS-ESI: Calculated for $C_{10}H_{12}NOINa$ $[M+Na]^+$ = 311.9856 m/z. Found: 311.9859 m/z.

The spectral properties of **2c** is in good agreement with literature data.[9]

***N*-Ethyl-2-iodobenzamide (2d)**

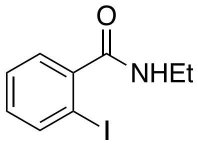

The title compound was prepared according the method A from *N*-ethylbenzamide (**1d**, 30.0 mg). Purification by column chromatography (Pentane:Acetone - 9:1) afforded **2d** as a colorless solid (34.1 mg, 62%).

^{1}H NMR (400 MHz, $CDCl_3$) δ = 7.85−7.82 (m, 1H), 7.37−7.33 (m, 2H), 7.09−7.05 (m, 1H), 5.82 (bs, 1H), 3.51−3.44 (m, 2H), 1.26 (t, J = 7.3 Hz, 3H) ppm. ^{13}C NMR (100 MHz, $CDCl_3$) δ = 169.3, 142.4, 139.7, 130.9, 128.2, 128.1, 92.4, 35.0, 14.6 ppm. HRMS-ESI: Calculated for $C_9H_{10}NOINa$ $[M+Na]^+$ = 297.9699 m/z. Found: 297.9719 m/z.

The spectral properties of **2d** is in good agreement with literature data.[8]

***N*-Benzyl-2-iodobenzamide (2e)**

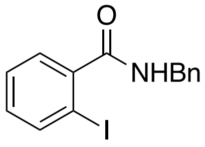

The title compound was prepared according the method A from *N*-benzylbenzamide (**1e**, 42.2 mg). Purification by column chromatography (Pentane:Acetone - 9:1) afforded **2e** as a colorless solid (35.1 mg, 52%). The product was obtained as a mixture of two rotamers at rt *ca.* 70:30.

^{1}H NMR (400 MHz, $CDCl_3$, mixture of two rotamers at rt *ca.* 70:30) δ = 7.84 (dd, J = 7.9, 1.1 Hz, 1H (both rotamers)), 7.68−7.65 (m, 1H (major rotamer)), 7.41−7.33 (m, 5H (both rotamers)), 7.41−7.33 (m, 2H (minor rotamer)), 7.16−7.13 (m, 1H (major rotamer)), 7.11−7.06 (m, 1H (both rotamers)), 6.22 (bs, 1H (minor rotamer)), 6.09 (bs, 1H (major rotamer)), 4.62 (d, J = 5.7 Hz, 2H (major rotamer)), 4.56 (d, J = 5.9 Hz, 2H (minor rotamer)) ppm. ^{13}C NMR (100 MHz, $CDCl_3$, mixture of two rotamers at rt *ca.* 70:30) δ = 169.21, 169.15, 142.0, 141.8, 139.89, 139.87, 138.9, 137.7, 137.5, 137.4, 131.2, 131.1, 130.0, 128.7, 128.27, 128.25, 128.15, 128.13, 127.7, 93.1, 92.41, 92.38, 44.2, 43.5 ppm. HRMS-ESI: Calculated for $C_{14}H_{12}NOINa$ $[M+Na]^+$ = 359.9856 m/z. Found: 359.9856 m/z.

2-Iodo-*N,N*-diisopropylbenzamide (2f)

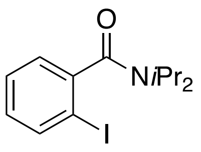

The title compound was prepared according the method B from *N,N*-diisopropylbenzamide (**1f**, 51.3 mg). Purification by column chromatography (Pentane:Acetone - 9:1) afforded **2f** as a colorless oil (74 mg, 89%).

^{1}H NMR (400 MHz, $CDCl_3$) δ = 7.80 (dd, *J* = 8.0, 1.1 Hz, 1H), 7.34 (td, *J* = 7.5, 1.1 Hz, 1H), 7.12 (dd, *J* = 7.6, 1.7 Hz, 1H), 7.02 (ddd, *J* = 8.0, 7.4, 1.7 Hz, 1H), 3.60–3.47 (m, 2H), 1.59 (d, *J* = 6.8 Hz, 3H), 1.55 (d, *J* = 6.8 Hz, 3H), 1.26 (d, *J* = 6.7 Hz, 3H), 1.06 (d, *J* = 6.7 Hz, 3H) ppm. ^{13}C NMR (100 MHz, $CDCl_3$) δ = 169.8, 144.1, 139.3, 129.4, 128.2, 125.8, 92.2, 51.2, 46.0, 20.7, 20.6*, 20.0* ppm.

*Methyl groups of one *i*Pr are not equal due to interaction with the iodide.

The spectral properties of **2f** is in good agreement with literature data.[9]

2-Iodo-*N,N*-dimethylbenzamide (2g)

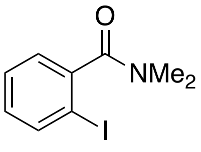

The title compound was prepared according the method B from *N,N*-dimethylbenzamide (**1g**, 37.3 mg). Purification by column chromatography (Pentane:Acetone - 9:1) afforded **2g** as a colorless oil (52.2 mg, 76%).

^{1}H NMR (400 MHz, $CDCl_3$) δ = 7.88 (dd, *J* = 8.0, 1.1 Hz, 1H), 7.38 (td, *J* = 7.5, 1.1 Hz, 1H), 7.20 (dd, *J* = 7.6, 1.7 Hz, 1H), 7.05 (ddd, *J* = 8.0, 7.4, 1.7 Hz, 1H), 3.12 (s, 3H), 2.83 (s, 3H) ppm. ^{13}C NMR (100 MHz, $CDCl_3$) δ = 170.7, 142.8, 139.0, 130.0, 128.3, 127.0, 92.3, 38.4, 34.7 ppm. HRMS-ESI: Calculated for $C_9H_{10}NOINa$ $[M+Na]^+$ = 297.9699 m/z. Found: 297.9728 m/z.

The spectral properties of **2g** is in good agreement with literature data.[9]

2-Iodobenzamide (2h)

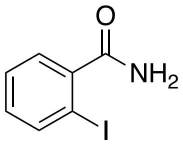

The title compound was prepared according the method B from benzamide (**1h**, 30.3 mg). Purification by column chromatography (Pentane:Acetone - 9:1) afforded **2h** as a colorless oil (13 mg, 21%).

^{1}H NMR (400 MHz, $CDCl_3$) δ = 7.90 (dd, *J* = 8.0, 1.1 Hz, 1H), 7.48 (dd, *J* = 7.6, 1.8 Hz, 1H), 7.40 (td, *J* = 7.6, 1.1 Hz, 1H), 7.12 (ddd, *J* = 8.0, 7.4, 1.8 Hz, 1H), 5.82 (bs, 2H) ppm. ^{13}C NMR (100 MHz, $CDCl_3$) δ = 170.8, 141.1, 140.2, 131.5, 128.4, 128.2, 92.0 ppm. HRMS-ESI: Calculated for C_7H_6NOINa $[M+Na]^+$ = 269.9386 m/z. Found: 269.9381 m/z.

The spectral properties of **2h** is in good agreement with literature data.[10]

Characterization data for *ortho*-diiodobenzamides 3

***N*-(*tert*-Butyl)-2,6-diiodobenzamide (3a)**

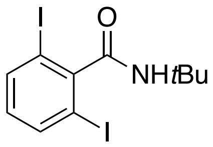

The title compound was prepared according the method A from *N*-(*tert*-butyl)benzamide (**1a**, 35.5 mg). Purification by column chromatography (SiO_2; petroleum ether / ethyl acetate, 90:10) afforded **3a** as a colorless solid (15 mg, 17%).

^{1}H NMR (400 MHz, $CDCl_3$) δ = 7.77 (d, *J* = 7.9, 2H), 6.69 (t, *J* = 7.9 Hz, 1H), 5.39 (bs, 1H), 1.51 (s, 9H) ppm. ^{13}C NMR (100 MHz, $CDCl_3$) δ = 168.5, 147.3, 139.0, 131.4, 92.3, 52.5, 28.5 ppm. HRMS-ESI: Calculated for $C_{11}H_{13}NOI_2Na$ $[M+Na]^+$ = 451.8979 m/z. Found: 451.9012 m/z.

2,6-Diiodo-*N*-isopropylbenzamide (3c)

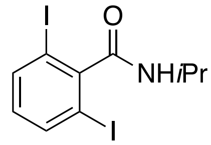

The title compound was prepared according the method A from *N*-isopropylbenzamide (**1c**, 32.6 mg). Purification by column chromatography (SiO_2; petroleum ether / ethyl acetate, 90:10) afforded **3c** as a colorless solid (7 mg, 8%).

^1H NMR (400 MHz, $CDCl_3$) δ = 7.79 (d, *J* = 7.9, 2H), 6.71 (t, *J* = 7.9 Hz, 1H), 5.43 (bs, 1H), 4.38–4.30 (m, 1H), 1.31 (d, *J* = 6.6 Hz, 6H) ppm. ^{13}C NMR (100 MHz, $CDCl_3$) δ = 168.7, 147.1, 139.0, 131.6, 92.2, 42.1, 22.4 ppm. HRMS-ESI: Calculated for $C_{10}H_{11}NOI_2Na$ $[M+Na]^+$ = 437.8822 m/z. Found: 437.8857 m/z.

***N*-Ethyl-2,6-diiodobenzamide (3d)**

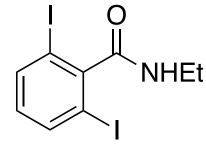

The title compound was prepared according the method A from *N*-ethylbenzamide (**1d**, 30.0 mg). Purification by column chromatography (Pentane:Acetone - 9:1) afforded **3d** as a colorless solid (6.9 mg, 9%).

^1H NMR (400 MHz, $CDCl_3$) δ = 7.9 (d, *J* = 7.9, 2H), 6.72 (t, *J* = 7.9 Hz, 1H), 5.61 (bs, 1H), 3.57–3.50 (m, 1H), 1.30 (d, *J* = 7.3 Hz, 3H) ppm. ^{13}C NMR (100 MHz, $CDCl_3$) δ = 169.6, 147.2, 139.0, 131.7, 92.2, 35.0, 14.4 ppm. HRMS-ESI: Calculated for $C_9H_9NOI_2Na$ $[M+Na]^+$ = 423.8666 m/z. Found: 423.8696 m/z.

Synthesis and characterization data for *ortho*-iodo-*N*-methoxy-*N*-methylbenzamides 5 and 6

2-Iodo-*N*-methoxy-*N*-methylbenzamide (5a)

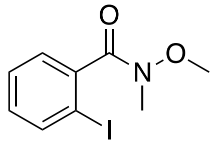

The title compound was prepared according the method B from *N*-methoxy-*N*-methylbenzamide (**4a**, 41 mg). Column chromatography (Pentane:Acetone - 9:1) afforded **5a** as a slightly orange oil (64 mg, 92% yield). The product was obtained as a mixture of two rotamers at rt *ca.* 75:25.

^{1}H NMR (400 MHz, $CDCl_3$, mixture of two rotamers at rt *ca.* 75:25) δ = 7.79 (dd, *J* = 8.0, 1.1 Hz, 1H (both rotamers)), 7.35 (td, *J* = 7.6, 1.1 Hz, 1H (both rotamers)), 7.23 (dd, *J* = 7.6, 1.7 Hz, 1H (both rotamers)), 7.05 (td, *J* = 7.7, 1.7 Hz, 1H (both rotamers)), 3.88 (bs, 3H (minor rotamer)), 3.42 (s, 3H (major rotamer)), 3.35 (s, 3H (major rotamer)), 3.08 (bs, 3H (minor rotamer)) ppm. ^{13}C NMR (100 MHz, $CDCl_3$, mixture of two rotamers at rt *ca.* 75:25) δ = 170.6, 141.5, 139.2, 138.8, 130.3, 127.5, 127.0, 92.3, 61.3, 36.3, 32.5 ppm.

The spectral properties of **5a** is in good agreement with literature data.[11,12]

2-Fluoro-6-iodo-*N*-methoxy-*N*-methylbenzamide (5b)

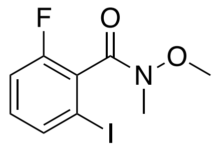

The title compound was prepared according the method B from 2-fluoro-*N*-methoxy-*N*-methylbenzamide (**4b**, 46 mg). Column chromatography (Pentane:Acetone - 9:1) afforded **5b** as a white solid (34 mg, 44% yield). The product was obtained as a mixture of two rotamers at rt *ca.* 80:20.

^{1}H NMR (400 MHz, $CDCl_3$, mixture of two rotamers at rt *ca.* 80:20) δ = 7.64 – 7.60 (m, 1H (both rotamers)), 7.12 – 7.06 (m, 2H (both rotamers)), 3.93 (s, 3H (minor rotamer)), 3.54 (s,

3H (major rotamer)), 3.41 (s, 3H (major rotamer)), 3.15 (s, 3H (minor rotamer)) ppm. ^{13}C NMR (100 MHz, $CDCl_3$, mixture of two rotamers at rt *ca.* 80:20) δ = 166.3, 158.0 (d, $J_{C\text{-}F}$ = 251.4 Hz), 135.0 (d, $J_{C\text{-}F}$ = 3.7 Hz), 134.6 (d, $J_{C\text{-}F}$ = 3.4 Hz), 132.1 (d, $J_{C\text{-}F}$ = 8 Hz), 131.4 (d, $J_{C\text{-}F}$ = 8.2 Hz), 130.3 (d, $J_{C\text{-}F}$ = 21.3 Hz), 115.8 (d, $J_{C\text{-}F}$ = 22.3 Hz), 115.3 (d, $J_{C\text{-}F}$ = 21.5 Hz), 99.9, 92.9 (d, $J_{C\text{-}F}$ = 3.4 Hz), 61.7, 60.7, 35.8, 32.5 ppm. ^{19}F NMR (376 MHz, $CDCl_3$, mixture of two rotamers at rt *ca.* 80:20) δ = −111.2 (minor rotamer), −111.5 (major rotamer) ppm.

The spectral properties of **5b** is in good agreement with literature data.[13]

4-Bromo-2-iodo-*N*-methoxy-*N*-methylbenzamide (5c)

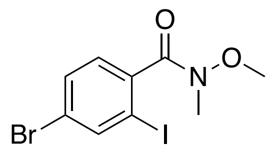

The title compound was prepared according the method B from 4-bromo-*N*-methoxy-*N*-methylbenzamide (**4c**, 62 mg). Column chromatography (Pentane:Acetone – 9:1) afforded **5c** as a white solid (82 mg, 88% yield). The product was obtained as a mixture of two rotamers at rt *ca.* 80:20.

^{1}H NMR (400 MHz, $CDCl_3$, mixture of two rotamers at rt *ca.* 80:20) δ = 7.99 (d, *J* = 1.9 Hz, 1H (both rotamers)), 7.52 (dd, *J* = 8.2, 1.9 Hz, 1H (both rotamers)), 7.13 (d, *J* = 8.1 Hz, 1H (both rotamers)), 3.92 (bs, 3H (minor rotamer)), 3.49 (s, 3H (major rotamer)), 3.40 (s, 3H (major rotamer)), 3.15 (bs, 3H (minor rotamer)) ppm. ^{13}C NMR (100 MHz, $CDCl_3$, mixture of two rotamers at rt *ca.* 80:20) δ = 169.8, 141.0, 140.5, 130.9, 128.3, 123.3, 93.1, 61.5, 32.6 ppm.

The spectral properties of **5c** is in good agreement with literature data.[12]

4-Fluoro-2-iodo-*N*-methoxy-*N*-methylbenzamide (5d)

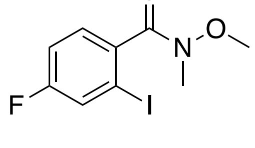

The title compound was prepared according the method B from 4-fluoro-*N*-methoxy-*N*-methylbenzamide (**4d**, 46 mg). Column chromatography (Pentane:Acetone – 9:1) afforded **5d**

as a colorless oil (60 mg, 78% yield). The product was obtained as a mixture of two rotamers at rt *ca.* 80:20.

^{1}H NMR (400 MHz, $CDCl_3$, mixture of two rotamers at rt *ca.* 80:20) δ = 7.54 (dd, J = 8.1, 2.5 Hz, 1H (both rotamers)), 7.23 (dd, J = 8.5, 5.7 Hz, 1H (both rotamers)), 7.09 (td, J = 8.3, 2.5 Hz, 1H (both rotamers)), 4.13 – 2.81 (m, 6H (both rotamers)) ppm. ^{13}C NMR (100 MHz, $CDCl_3$, mixture of two rotamers at rt *ca.* 80:20) δ = 169.9, 161.9 (d, $J_{C\text{-}F}$ = 253.8 Hz), 137.6, 128.4, 126.1 (d, $J_{C\text{-}F}$ = 23.9 Hz), 115.0 (d, $J_{C\text{-}F}$ = 18.4 Hz), 92.3 (d, $J_{C\text{-}F}$ = 8.2 Hz), 61.4, 32.6 ppm. ^{19}F NMR (376 MHz, $CDCl_3$, mixture of two rotamers at rt *ca.* 80:20) δ = −109.03 (minor rotamer), −110.24 (major rotamer) ppm.

The spectral properties of **5d** is in good agreement with literature data.[11]

2-Iodo-*N*-methoxy-*N*,4-dimethylbenzamide (5e)

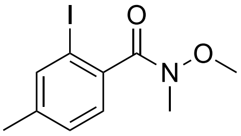

The title compound was prepared according the method B from *N*-methoxy-*N*,4-dimethylbenzamide (**4e**, 45 mg). Column chromatography (Pentane:Acetone – 9:1) afforded **5e** as a colorless oil (45 mg, 60% yield). The product was obtained as a mixture of two rotamers at rt *ca.* 75:25.

^{1}H NMR (400 MHz, $CDCl_3$, mixture of two rotamers at rt *ca.* 75:25) δ = 7.67–7.66 (m, 1H (both rotamers)), 7.19–7.14 (m, 2H (both rotamers)), 3.88 (bs, 3H (minor rotamer)), 3.47 (s, 3H (major rotamer)), 3.37 (s, 3H (major rotamer)), 3.13 (bs, 3H (minor rotamer)), 2.33 (s, 3H (both rotamer)) ppm. ^{13}C NMR (100 MHz, $CDCl_3$, mixture of two rotamers at rt *ca.* 75:25) δ = 170.8, 140.8, 139.6, 138.7, 128.8, 128.6, 128.5, 127.2, 92.6, 61.5, 32.8, 20.9 ppm.

The spectral properties of **5e** is in good agreement with literature data.[14]

4-(Hydroxymethyl)-2-iodo-*N*-methoxy-*N*-methylbenzamide (5f)

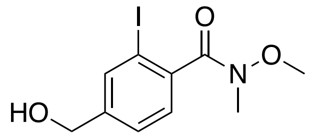

The title compound was prepared according the method B from 4-(hydroxymethyl)-*N*-methoxy-*N*-methylbenzamide (**4f**, 49 mg). Column chromatography (Pentane:Acetone – 9:1) afforded **5f** as a colorless oil (53 mg, 66% yield). The product was obtained as a mixture of two rotamers at rt *ca.* 75:25.

^{1}H NMR (400 MHz, $CDCl_3$, mixture of two rotamers at rt *ca.* 75:25) δ = 7.80 (m, 1H (both rotamers)), 7.34 (ddd, J = 7.8, 1.5, 0.8 Hz, 1H (both rotamers)), 7.22 (d, J = 7.8 Hz, 1H (both rotamers)), 4.66 (d, J = 4.4 Hz, 2H (both rotamers)), 3.91 (bs, 3H (minor rotamer)), 3.45 (s, 3H (major rotamer)), 3.38 (s, 3H (major rotamer)), 3.10 (bs, 3H (minor rotamer)), 2.62 (bs, 1H (minor rotamer)), 2.45 (bs, 1H (major rotamer)) ppm. ^{13}C NMR (100 MHz, $CDCl_3$, mixture of two rotamers at rt *ca.* 75:25) = 170.8, 143.8, 140.3, 136.9, 127.1, 125.8, 92.5, 63.6, 61.5, 32.6 ppm. HRMS-ESI: Calculated for $C_{10}H_{12}NO_3INa$ $[M+Na]^+$ = 343.9754 m/z. Found: 343.9758 m/z.

4-(2-Chloroethyl)-2-iodo-*N*-methoxy-*N*-methylbenzamide (5g)

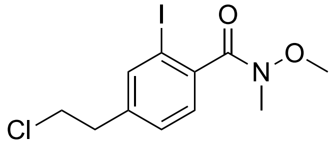

The title compound was prepared according the method B from 4-(2-chloroethyl)-*N*-methoxy-*N*-methylbenzamide (**4g**, 57 mg). Column chromatography (Pentane:Acetone – 9:1) afforded **5g** as a colorless oil (78 mg, 88% yield). The product was obtained as a mixture of two rotamers at rt *ca.* 75:25.

^{1}H NMR (400 MHz, $CDCl_3$, mixture of two rotamers at rt *ca.* 75:25) δ = 7.70–7.69 (m, 1H (both rotamers)), 7.24–7.20 (m, 2H (both rotamers)), 3.91 (bs, 3H (minor rotamer)), 3.69 (t, J = 7.2 Hz, 2H (both rotamers)), 3.46 (s, 3H (major rotamer)), 3.38 (s, 3H (major rotamer)), 3.12 (bs, 3H (minor rotamer)), 3.03 (t, J = 7.2 Hz, 2H (both rotamers)) ppm. ^{13}C NMR (100 MHz, $CDCl_3$, mixture of two rotamers at rt *ca.* 75:25) = 170.8, 140.8, 140.3, 139.4, 128.4, 127.4,

92.8, 61.6, 44.3, 38.3, 32.8 ppm. HRMS-ESI: Calculated for $C_{11}H_{13}NO_2I^{35}ClNa$ $[M+Na]^+$ = 375.9572 m/z. Found: 375.9582 m/z.

Methyl 3-iodo-4-(methoxy(methyl)carbamoyl)benzoate (5h)

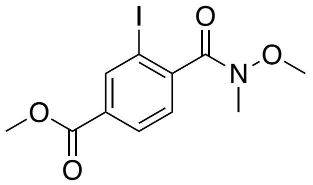

The title compound was prepared according the method B from methyl 4-(methoxy(methyl)carbamoyl)benzoate (**4h**, 56 mg). Column chromatography (Pentane:Acetone – 9:1) afforded **5h** as a white solid (68 mg, 78% yield).

^{1}H NMR (400 MHz, $CDCl_3$) δ = 8.51 (d, *J* = 1.5 Hz, 1H), 8.06 (dd, *J* = 8.0, 1.6 Hz, 1H), 7.34 (d, *J* = 7.9 Hz, 1H), 3.95 (s, 3H), 3.92 – 3.05 (m, 6H) ppm. ^{13}C NMR (101 MHz, $CDCl_3$) δ = 170.0, 165.0, 145.9, 139.9, 131.8, 129.3, 128.8, 128.1, 127.0, 92.1, 61.5, 52.6, 32.6 ppm.

The spectral properties of **5h** is in good agreement with literature data.[12]

3,5-Difluoro-2-iodo-*N*-methoxy-*N*-methylbenzamide (5i)

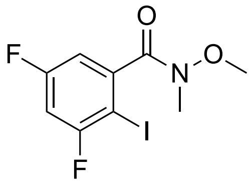

The title compound was prepared according the method B from methyl 3,5-difluoro-*N*-methoxy-*N*-methylbenzamide (**4i**, 50 mg). Column chromatography (Pentane:Acetone – 9:1) afforded **5i** as a white solid (69 mg, 85% yield).

^{1}H NMR (400 MHz, $CDCl_3$, mixture of two rotamers at rt *ca.* 75:25) δ = 6.88–6.83 (m, 2H (both rotamers)), 3.91 (bs, 3H (minor rotamer)), 3.48 (s, 3H (major rotamer)), 3.37 (s, 3H (major rotamer)), 3.10 (bs, 3H (minor rotamer)) ppm. ^{13}C NMR (100 MHz, $CDCl_3$, mixture of two rotamers at rt *ca.* 75:25) = 168.5, 163.0 (dd, $J_{C\text{-}F}$ = 251.5, 11.2 Hz), 162.0 (dd, $J_{C\text{-}F}$ = 248.3, 11.8 Hz), 144.6 (d, $J_{C\text{-}F}$ = 8.0 Hz), 110.7 (d, $J_{C\text{-}F}$ = 23.9 Hz), 104.5 (t, $J_{C\text{-}F}$ = 27.0 Hz), 74.7 (dd, $J_{C\text{-}F}$ = 28.0, 4.3 Hz), 61.7, 60.8, 36.5, 32.7 ppm. ^{19}F NMR (376 MHz, $CDCl_3$, mixture of two

rotamers at rt *ca.* 80:20) δ = −85.7 (minor rotamer), −86.9 (d, *J* = 9 Hz, major rotamer), −107.7 (minor rotamer), −109.3 (d, *J* = 9 Hz, major rotamer) ppm. HRMS-ESI: Calculated for $C_9H_8NF_2O_2INa$ $[M+Na]^+$ = 349.9460 m/z. Found: 349.9456 m/z.

4-Iodo-*N*-methoxy-*N*-methylbenzo[d][1,3]dioxole-5-carboxamide (5j)

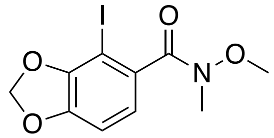

The title compound was prepared according the method B from *N*-methoxy-*N*-methyl-1,3-benzodioxole-5-carboxamide (**4j**, 52 mg). Column chromatography (Pentane:Acetone – 9:1) afforded **5j** as a white solid (27 mg, 32% yield).

Melting point 130.0-131.6 °C. ^1H NMR (500 MHz, $CDCl_3$) δ = 6.80 (d, *J* = 7.9 Hz, 1H), 6.77 (d, *J* = 7.9 Hz, 1H), 6.05 (s, 2H), 3.85 – 3.03 (m, 6H) ppm. ^{13}C NMR (125 MHz, $CDCl_3$) δ = 170.0 (br), 149.8, 146.6, 134.3, 121.3, 108.1, 100.9, 70.3, 61.3, 33.1 (br) ppm. HRMS-ESI: Calculated for $C_{10}H_{10}NO_4INa$ $[M+Na]^+$ = 357.9547 m/z. Found: 357.9564 m/z.

3-Iodo-*N*-methoxy-*N*-methylfuran-2-carboxamide (5k)

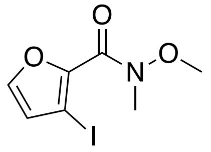

The title compound was prepared according the method B from *N*-methoxy-*N*-methylfuran-2-carboxamide (**4k**, 39 mg). Column chromatography (Pentane:Acetone – 9:1) afforded **5k** as a white solid (48mg, 68% yield).

Melting point 78.4-80.0 °C. ^1H NMR (400 MHz, $CDCl_3$) δ = 7.01 (dd, *J* = 3.5, 1.5 Hz, 1H), 6.67 (dd, *J* = 3.5, 1.5 Hz, 1H), 3.76 (s, 3H), 3.33 (s, 3H) ppm. ^{13}C NMR (100 MHz, $CDCl_3$) δ = 158.0, 151.2, 122.3, 119.7, 93.7, 61.5, 33.2 ppm. HRMS-ESI: Calculated for $C_7H_8NO_3INa$ $[M+Na]^+$ = 303.9441 m/z. Found: 303.9443 m/z.

2-Iodo-*N*-methoxy-*N*,5-dimethylbenzamide + 2-iodo-*N*-methoxy-*N*,3-dimethylbenzamide (5l + 6l)

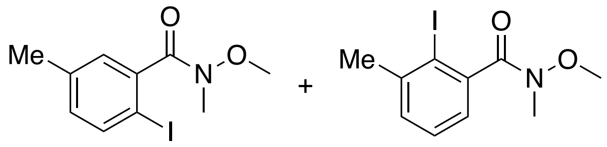

The title compound was prepared according the method B from *N*-methoxy-*N,*3-dimethylbenzamide (**4l**, 45 mg). Column chromatography (Pentane:Acetone – 9:1) afforded **5l** and **6l** (9:1 mixture) as a slightly yellow solid (72 mg, 94% yield).

Compound **5l**:

^{1}H NMR (500 MHz, $CDCl_3$, mixture of two rotamers at rt *ca.* 70:30) δ = 7.67 (d, *J* = 8.1 Hz, 1H (both rotamers)), 7.07 – 7.06 (m, 1H (both rotamers)), 6.90 – 6.88 (m, 1H (both rotamers)), 3.91(bs, 3H (minor rotamer)), 3.46 (s, 3H (major rotamer)), 3.37 (s, 3H (major rotamer)), 3.08 (bs, 3H (minor rotamer)), 2.31 (s, 3H (both rotamers)) ppm. ^{13}C NMR (125 MHz, $CDCl_3$, mixture of two rotamers at rt *ca.* 70:30) δ = 170.7, 166.5, 141.4, 140.7, 138. 9, 138.5, 137.7, 131.8, 131.2, 128.2, 127.8, 88.2, 61.4, 60.4, 36.3, 32.6, 20.8 ppm.

The spectral properties of **5l** is in good agreement with literature data.[12]

Compound **6l**:

Selected signals: ^{1}H NMR (500 MHz, $CDCl_3$) δ = 7.30 − 7.21 (m, 2H), 7.03 (dd, *J* = 7.2, 1.9 Hz, 1H), 3.46 (s, 3H), 3.37 (s, 3H), 2.46 (s, 3H) ppm. ^{13}C NMR (125 MHz, $CDCl_3$) δ = 171.5, 142.8, 142.1, 129.6, 124.0, 99.1, 28.7 ppm.

The spectral properties of **6l** is in good agreement with literature data.[13]

5-Bromo-2-iodo-*N*-methoxy-*N*-methylbenzamide (5m)

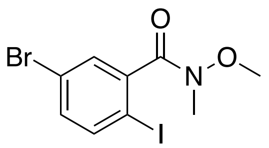

The title compound was prepared according the method B from 3-bromo-*N*-methoxy-*N*-methylbenzamide (**4m**, 62 mg). Column chromatography (Pentane:Acetone – 9:1) afforded **5m** as a pale white solid (53 mg, 57% yield). The product was obtained as a mixture of two rotamers at rt *ca.* 75:25.

Melting point: 80.3-85.0 °C. ^{1}H NMR (400 MHz, $CDCl_3$, mixture of two rotamers at rt *ca.* 75:25) δ = 7.65 (d, *J* = 8.4 Hz, 1H (both rotamers)), 7.37 (d, *J* = 2.4 Hz, 1H (both rotamers)), 7.20 (dd, *J* = 8.4, 2.4 Hz, 1H (both rotamers)), 3.89 (bs, 3H (minor rotamer)), 3.47 (s, 3H (major rotamer)), 3.36 (s, 3H (major rotamer)), 3.11 (bs, 3H (minor rotamer)) ppm. ^{13}C NMR (100 MHz, $CDCl_3$, mixture of two rotamers at rt *ca.* 75:25) δ = 169.0, 143.3, 140.6, 140.2, 133.9, 133.4, 130.4, 130.0, 121.9, 121.8, 90.4, 61.5, 60.5, 36.4, 32.6 ppm. HRMS-ESI: Calculated for $C_9H_9NO_2I^{79}BrNa$ $[M+Na]^+$ = 391.8754 m/z. Found: 391.8745 m/z.

3-Bromo-2-iodo-*N*-methoxy-*N*-methylbenzamide (6m)

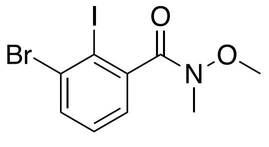

The title compound was prepared according the method B from 3-bromo-*N*-methoxy-*N*-methylbenzamide (**4m**, 62 mg). Column chromatography (Pentane:Acetone – 9:1) afforded **6m** as a colorless oil (37 mg, 40% yield). The product was obtained as a mixture of two rotamers at rt *ca.* 80:20.

^{1}H NMR (400 MHz, $CDCl_3$, mixture of two rotamers at rt *ca.* 80:20) δ = 7.63 (dd, *J* = 7.7, 1.5 Hz, 1H (both rotamers)), 7.24 (t, *J* = 7.7 Hz, 1H (both rotamers)), 7.13 (dd, *J* = 7.4, 1.5 Hz, 1H (both rotamers)), 3.91 (s, 3H (minor rotamer)), 3.46 (s, 3H (major rotamer)), 3.37 (s, 3H (major rotamer)), 3.10 (s, 3H (minor rotamer)) ppm. ^{13}C NMR (100 MHz, $CDCl_3$, mixture of two rotamers at rt *ca.* 80:20) δ = 170.4, 144.9, 133.2, 132.7, 131.0, 129.9, 129.2, 125.5, 125.0, 99.9,

61.4, 60.4, 36.3, 32.8 ppm. HRMS-ESI: Calculated for $C_9H_9NO_2I^{79}BrNa$ $[M+Na]^+$ = 391.8754 m/z. Found: 391.8763 m/z.

5-Fluoro-2-iodo-*N*-methoxy-*N*-methylbenzamide (5n)

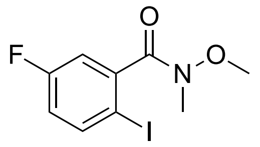

The title compound was prepared according the method B from 3-fluoro-*N*-methoxy-*N*-methylbenzamide (**4n**, 46 mg). Column chromatography (Pentane:Acetone – 9:1) afforded **5n** as a colorless oil (6 mg, 8% yield). The product was obtained as a mixture of two rotamers at rt *ca.* 75:25.

Selected signals: ^{1}H NMR (400 MHz, $CDCl_3$, mixture of two rotamers at rt *ca.* 75:25) δ = 7.77 (dd, J = 8.7, 5.2 Hz, 1H), 7.02 (dd, J = 8.3, 3.0 Hz, 1H), 6.86 (td, J = 8.5, 3.0 Hz, 1H), 3.93 (bs, 3H (minor rotamer)), 3.50 (s, 3H (major rotamer)), 3.39 (s, 3H (major rotamer)), 3.13 (bs, 3H (minor rotamer)) ppm. ^{19}F NMR (376 MHz, $CDCl_3$, mixture of two rotamers at rt *ca.* 75:25) δ = −112.1 (minor rotamer), −113.6 (major rotamer) ppm.

The spectral properties of **5n** is in good agreement with literature data.[13]

3-Fluoro-2-iodo-*N*-methoxy-*N*-methylbenzamide (6n)

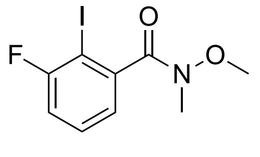

The title compound was prepared according the method B from 3-fluoro-*N*-methoxy-*N*-methylbenzamide (**4n**, 46 mg). Column chromatography (Pentane:Acetone – 9:1) afforded **6n** as a colorless oil (60 mg, 85% yield). The product was obtained as a mixture of two rotamers at rt *ca.* 80:20.

^{1}H NMR (400 MHz, $CDCl_3$, mixture of two rotamers at rt *ca.* 80:20) δ = 7.38–7.33 (m, 1H (both rotamers)), 7.09–7.05 (m, 2H (both rotamers)), 3.92 (bs, 3H (minor rotamer)), 3.46 (s, 3H (major rotamer)), 3.39 (s, 3H (major rotamer)), 3.10 (bs, 3H (minor rotamer)) ppm. ^{13}C

NMR (100 MHz, $CDCl_3$, mixture of two rotamers at rt *ca.* 80:20) δ = 169.8, 161.7 (d, $J_{C\text{-}F}$ = 246.4 Hz), 144.1, 130.8 (d, $J_{C\text{-}F}$ = 5.8 Hz), 130.0 (d, $J_{C\text{-}F}$ = 7.4 Hz), 116.4 (d, $J_{C\text{-}F}$ = 19.4 Hz), 115.8 (d, $J_{C\text{-}F}$ = 24.4 Hz), 80.9 (d, $J_{C\text{-}F}$ = 27.6 Hz), 61.6, 60.8, 36.7, 32.7 ppm. ^{19}F NMR (376 MHz, $CDCl_3$, mixture of two rotamers at rt *ca.* 80:20) δ = −90.2 (minor rotamer), −91.3 (major rotamer) ppm.

The spectral properties of **6n** is in good agreement with literature data.[15]

2-Iodo-*N*-methoxy-*N*-methyl-5-nitrobenzamide (5o)

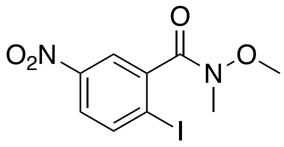

The title compound was prepared according the method B from *N*-methoxy-*N*-methyl-3-nitrobenzamide (**4o**, 52.5 mg). Column chromatography (Pentane:Acetone – 9:1) afforded **5o** as a white solid (16 mg, 19% yield). The product was obtained as a mixture of two rotamers at rt *ca.* 90:10.

Melting point: 129.6-132.1 °C. ^{1}H NMR (400 MHz, $CDCl_3$, mixture of two rotamers at rt *ca.* 90:10) δ = 8.12 (d, J = 2.8 Hz, 1H (both rotamers)), 8.07 (d, J = 8.6 Hz, 1H (both rotamers)), 7.93 (dd, J = 8.6, 2.6 Hz, 1H (both rotamers)), 3.92 (bs, 3H (minor rotamer)), 3.49 (s, 3H (major rotamer)), 3.42 (s, 3H (major rotamer)), 3.16 (bs, 3H (minor rotamer)) ppm. ^{13}C NMR (100 MHz, $CDCl_3$, mixture of two rotamers at rt *ca.* 90:10) δ = 168.4, 147.6, 143.3, 140.3, 124.5, 121.9, 101.0, 61.7, 32.7 ppm. HRMS-ESI: Calculated for $C_9H_9N_2O_4INa$ $[M+Na]^+$ = 358.9499 m/z. Found: 358.9482 m/z.

2-Iodo-*N*-methoxy-*N*-methyl-3-nitrobenzamide (6o)

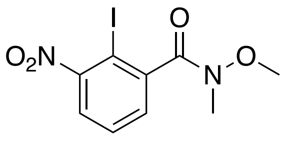

The title compound was prepared according the method B from *N*-methoxy-*N*-methyl-3-nitrobenzamide (**4o**, 52.5 mg). Column chromatography (Pentane:Acetone – 9:1) afforded **6o**

as a yellow solid (16 mg, 19% yield). The product was obtained as a mixture of two rotamers at rt *ca.* 85:15.

Melting point: 125.5-127.0 °C. ^{1}H NMR (400 MHz, $CDCl_3$, mixture of two rotamers at rt *ca.* 85:15) δ = 7.76 (dd, *J* = 8.0, 1.6 Hz, 1H (both rotamers)), 7.55 (dd, *J* = 8.0, 7.8 Hz, 1H (both rotamers)), 7.43 (dd, *J* = 7.6, 1.5 Hz, 1H (both rotamers)), 3.97 (bs, 3H (minor rotamer)), 3.50 (s, 3H (major rotamer)), 3.43 (s, 3H (major rotamer)), 3.15 (bs, 3H (minor rotamer)) ppm. ^{13}C NMR (100 MHz, $CDCl_3$, mixture of two rotamers at rt *ca.* 85:15) δ = 169.5, 154.1, 145.7, 130.3, 129.7, 129.0, 125.3, 124.8, 85.1, 61.6, 60.6, 36.5, 32.8 ppm. HRMS-ESI: Calculated for $C_9H_9N_2O_4INa$ $[M+Na]^+$ = 358.9499 m/z. Found: 358.9494 m/z.

Synthesis and characterization data for deuterated benzamides 1a-*d₂* and 4a-*d₂*

Benzoic acid (490 mg, 4.0 mmol), $[Cp^*IrCl_2]_2$ (63.7 mg, 2 mol%) were suspended in D_2O (20 mL) in a microwave vial. The contents were sonicated for 2 minutes, after which the mixture was heated in a microwave reactor at 170 °C for 1 hour. The resulting mixture was extracted with CH_2Cl_2 (3 x 30 mL). The Organic phase was dried over $MgSO_4$ and concentrated *in vacuo*. The residue was dissolved in Et_2O (20 mL), upon which most of the present catalyst precipitated. The product was directly submitted to the next reaction without further purification.

***N*-(*tert*-Butyl)benzamide-2,6-*d*$_2$ (1a-*d*$_2$)**

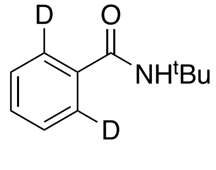

To a solution of benzoic-2,6-*d*$_2$ acid (250 mg, 2 mmol) in CH_2Cl_2 (10 mL), $SOCl_2$ (0.18 mL, 2.4 mmol) and DMF (2 drops) were added. After 3 h reactions, the mixture was concentrated *in vacuo* and potassium carbonate (552 mg, 4 mmol), and THF (20 mL) were added. To the reaction mixture, *tert*-butylamine (0.15 mL, 2 mmol) was added. The flask was capped with a septum and the reaction was allowed to stir. After 12 hours the reaction was quenched with 1 M HCl and extracted with AcOEt (3 x 30 ml), washed with brine and dried over $MgSO_4$, and concentrated *in vacuo.* The product was purified by column chromatography (petroleum ether / ethyl acetate) providing a white solid **1a**-*d*$_2$ (325 mg, 91%, 98% D).

^1H NMR (400 MHz, $CDCl_3$) δ = 7.48–7.44 (m, 1H), 7.40–7.38 (m, 2H), 5.96 (brs, 1H), 1.47 (s, 9H) ppm. ^{13}C NMR (100 MHz, $CDCl_3$) δ = 167.0, 135.9, 131.2, 128.5, 126.5 (t, $^1J_{C\text{-}D}$ = 24.1 Hz), 51.7, 29.0 ppm. HRMS-ESI: Calculated for $C_{11}H_{13}NOD_2Na$ $[M+Na]^+$ = 202.1171 m/z. Found: 202.1180 m/z.

***N*-Methoxy-*N*-methylbenzamide-2,6-*d*₂ (4a-*d*₂)**

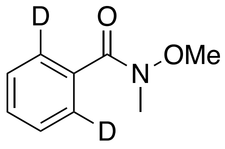

To a solution of benzoic-2,6-d_2 acid (250 mg, 2 mmol) in CH_2Cl_2 (10 mL), $SOCl_2$ (0.18 mL, 2.4 mmol) and DMF (2 drops) were added. After 3 h reactions, the mixture was concentrated *in vacuo* and N-methoxymethylamine hydrochloride salt (234 mg, 2 mmol) in CH_2Cl_2 (10 mL). To the reaction mixture, triethylamine (0.4 mL, 4 mmol) was added. After 12 hours the reaction was quenched with $NaHCO_3$ solution (15 ml) and extracted with CH_2Cl_2 (3 x 30 ml), washed with brine and dried over $MgSO_4$, and concentrated *in vacuo.* The product was purified by column chromatography (petroleum ether / ethyl acetate) providing an oil **4a**-d_2 (318 mg, 95%, 98% D).

^1H NMR (400 MHz, $CDCl_3$) δ = 7.44–7.41 (m, 1H), 7.38–7.36 (m, 2H), 3.52 (s, 3H), 3.33 (s, 3H) ppm. ^{13}C NMR (100 MHz, $CDCl_3$, mixture of two rotamers at rt) δ = 170.0, 169.1, 133.9, 133.1, 130.6, 130.5, 128.2, 127.92, 127.90 (t, $^1J_{C\text{-}D}$ = 28 Hz), 61.1, 33.8 ppm. HRMS-ESI: Calculated for $C_9H_9NO_2D_2Na$ $[M+Na]^+$ = 190.0808 m/z. Found: 190.0797 m/z.

Synthesis and characterization data for deuterated *ortho*-iodo-benzamides 2a-*d* and 5a-*d*

***N*-(*tert*-Butyl)-2-iodobenzamide-6-*d* (2a-*d*)**

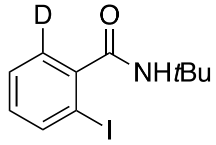

N-(*tert*-Butyl)benzamide-2,6-*d*$_2$ (**1a**-*d*$_2$, 36 mg, 0.2 mmol), [Cp*Ir(H_2O)$_3$]SO_4 (3.0 mol%, 0.006 mmol, 3 mg), *N*-iodosuccinimide (0.3 mmol, 68 mg) and trifluoroacetic acid (0.06 mmol, 5 μL) were dissolved in 1,1,1,3,3,3-hexafluoro-2-propanol (HFIP, 0.1 M, 2.0 mL) under air atmosphere in a capped vial and stirred at 40 °C (oil bath) for 16 hours. The reaction was quenched by addition of $Na_2S_2O_3$ (sat) (2 mL). The aqueous phase was extracted with CH_2Cl_2 (3 x 20 mL), and the combined organic phases were washed with brine (10 mL), dried over $MgSO_4$, and concentrated under reduced pressure. The product was purified by column chromatography (petroleum ether / ethyl acetate) providing the *ortho*-iodobenzamide **2a**-*d* (41 mg, 67%).

^{1}H NMR (400 MHz, $CDCl_3$) δ = 7.83 (dd, *J* = 8.0, 1.1 Hz, 1H), 7.36–7.34 (m, 1H), 7.06 (t, *J* = 7.7 Hz, 1H), 5.54 (bs, 1H), 1.48 (s, 9H) ppm. ^{13}C NMR (100 MHz, $CDCl_3$) δ = 168.8, 143.3, 139.8, 130.9, 128.2, 128.1 (t, $^1J_{C\text{-}D}$ = 10.1 Hz), 92.5, 52.4, 28.9 ppm. HRMS-ESI: Calculated for $C_{11}H_{13}NDOINa$ $[M+Na]^+$ = 327.0075 m/z. Found: 327.0062 m/z.

2-Iodo-*N*-methoxy-*N*-methylbenzamide-6-*d* (5a-*d*)

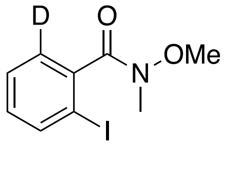

N-Methoxy-*N*-methylbenzamide-2,6-*d*$_2$ (**4a**-*d*$_2$, 42 mg, 0.25 mmol, 98%D) and [Cp*Ir(H_2O)$_3$]SO_4 (3.0 mol%, 7.5 μmol, 3.5 mg) was dissolved in HFIP (0.1 M, 2.5 mL) under air atmosphere in a capped vial and trifluoroacetic acid (0.125 mmol, 10 μL) was added. When the reagents were completely dissolved *N*-iodosuccinimide (0.375 mmol, 84 mg) was added in three portions waiting 2 hours between addition and stirring the reaction at 60 °C (oil bath). After 6 hours the reaction was quenched by the addition of $Na_2S_2O_3$ (aq, sat. 0.5 mL) and water (20 mL), extracted with DCM (25 mL), dried with $MgSO_4$, filtered and evaporated. The product was purified by column chromatography (petroleum ether / ethyl acetate) providing an oil **5a**-*d* (66 mg, 90%, 98% D).

^{1}H NMR (400 MHz, $CDCl_3$, mixture of two rotamers at rt *ca.* 75:25) δ = 7.83–7.81 (m, 1H (both rotamers)), 7.37 (d, *J* = 7.5 Hz, 1H (both rotamers)), 7.10–7.06 (m, 1H (both rotamers)), 3.91 (bs, 3H (minor rotamer)), 3.45 (s, 3H (major rotamer)), 3.38 (s, 3H (major rotamer)), 3.11 (bs, 3H (minor rotamer)) ppm. ^{13}C NMR (100 MHz, $CDCl_3$, mixture of two rotamers at rt *ca.*

75:25) δ = 170.8, 141.7, 139.0, 130.4, 128.4, 127.6, 127.2, 126.7, 92.6, 92.4, 61.5, 60.6, 36.6, 32.7 ppm.* HRMS-ESI: Calculated for $C_9H_9NO_2DINa$ $[M+Na]^+$ = 314.9711 m/z. Found: 314.9706 m/z.* Broad signals of aromatic carbons, *J* of C–D bond was not possible to determine.

Kinetic Isotope Effect

[**1a**-*d*$_2$] and [**1a**]

Ten parallel reactions, five with *N*-(*tert*-butyl)benzamide (**1a**) and other five with *N*-(*tert*-butyl)benzamide-2,6-*d*$_2$ (98% D, **1a**-*d*$_2$), were carried out. Benzamides **1a** or **1a**-*d*$_2$ (0.1 mmol), NIS (34 mg, 0.15 mmol) and $[Cp^*Ir(H_2O)_3]SO_4$ (3 mol%, 2 mg) were dissolved in HFIP (0.1 M, 1.0 mL) and TFA (3 µL, 0.3 equiv.) in a capped vial. The reactions were quenched at 1 min, 2 min, 3 min, 4 min and 5 min. Signals from the aromatic protons of the product were used to monitor the formation of *N*-(*tert*-butyl)-2-iodobenzamide (**2a**). Each experiment was performed by duplicate. The average of the initial rate plots for the experiments with each allylic alcohol (**1a**, **1a**-***d***$_2$) are given in Figure S1. A KIE of 1.96 ± 0.11 was obtained.

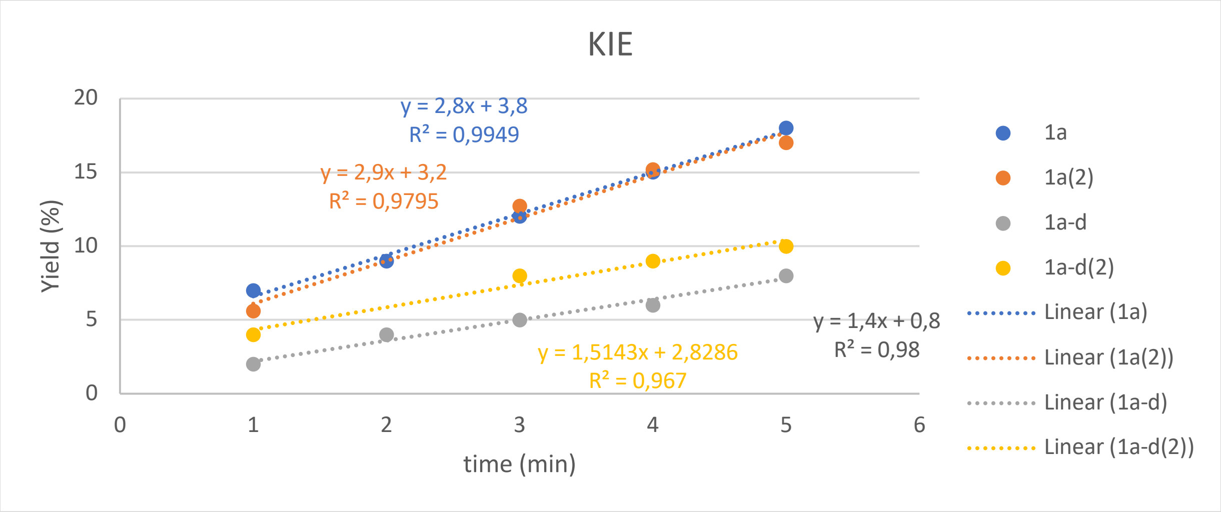

Figure S1

[**4a**-*d*$_2$] and [**4a**]

Ten parallel reactions, five with *N*-methoxy-*N*-methylbenzamide (**4a**) and other five with *N*-(*tert*-butyl)benzamide-2,6-*d*$_2$ (98% D, **4a**-*d*$_2$), were carried out. Benzamides **4a** or **4a**-*d*$_2$ (0.25 mmol), NIS (84 mg, 0.375 mmol) and $[Cp^*Ir(H_2O)_3]SO_4$ (3 mol%, 4 mg) were dissolved in

HFIP (0.1 M, 2.5 mL) and TFA (10 µL, 0.5 equiv.) in a capped vial. The reactions were quenched at 1 min, 2 min, 3 min, 4 min and 5 min. Signals from the aromatic protons of the product were used to monitor the formation of 2-iodo-*N*-methoxy-*N*-methylbenzamide (**5a**). Each experiment was performed by duplicate. The average of the initial rate plots for the experiments with each allylic alcohol (**5a**, **5a-*d*$_2$**) are given in Figure S2. A KIE of 2.77 ± 0.38 was obtained.

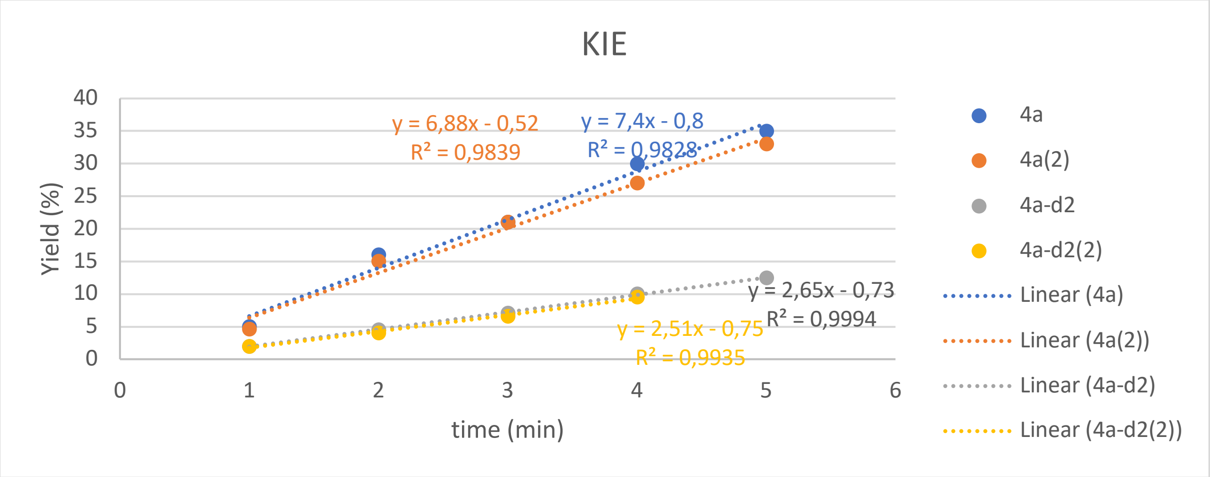

Figure S2

REFERENCES

[1] Hyster, T. K.; Rovis, T. *J. Am. Chem. Soc.* **2010**, *132*, 10565–10569

[2] Hanada, S.; Tsutsumi, E.; Motoyama, Y.; Nagashima, H. *J. Am. Chem. Soc.* **2009**, *131*, 15032–15040

[3]Tsukasa, M.; Shinya, O.; Hiroaki, O.; Kazuya, S.; Masao, M.; Nobutaka, F. *Bioorg. Med. Chem.* **2013**, *21*, 2079-2087

[4] Kerr, W. J.; Morrison, A. J.; Pazicky, M.; Weber, T. *Org. Lett.* **2012**, *14*, 2250-2253.

[5] Ogo, S.; Makihara, N.; Watanabe, Y. *Organometallics* **1999**, *18*, 5470-5474

[6] Yu, D.-G.; Gensch, T.; de Azambuja, F.; Vaśquez-Ceśpedes, S.; Glorius, F.*J. Am. Chem. Soc.* **2014**, 136, 17722−17725

[7] Liu, X.; Han, W.; Li, C.; Ma, Z.; Li, R.; Zheng, X.; Fu, H.; Chen, H. *Eur. J. Org. Chem. 2016*, 389–393

[8] Zhou, Y.; Zhu, J.; Li, B.; Zhang, Y.; Feng, J.; Hall, A.; Shi, J.; Zhu, W. *Org. Lett.* **2016**, *18*, 380−383

[9] Qiu, F.-C.; Yang, W.-C.; Chang, Y.-Z.; Guan, B.-T. *Asian J. Org. Chem.* **2017**, *6*,1361 –1364

[10] Brahmchari, D.; Verma, A. K.; Mehta, S.*J. Org. Chem.* **2018**, *83*, 3339−3347

[11] Das, R.; Kapur, M. *J. Org. Chem.* **2017**, *82*, 1114−1126

[12] Kawai, K.; Bunno, Y.; Yoshino, T.; Matsunaga, S. *Chem. Eur. J.* **2018**, *24*, 10231-10237

[13] Jithunsa, M.; Ueda, M.; Miyata, O. *Org. Lett.* **2011**, *13*, 518-521

[14] Das, R.; Kapur, M. *J. Org. Chem.* **2017**, *82*, 1114−1126

[15] Kawai, K.; Bunno,Y.; Yoshino, T.; Matsunaga, S. *Chem. Eur. J.* **2018**, *24*,10231–10237

^{1}H NMR and ^{13}C NMR of *ortho*-halogenated products 2, 3 and 5

***N*-(*tert*-Butyl)-2-iodobenzamide (2a)**

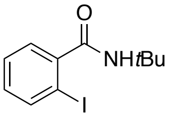

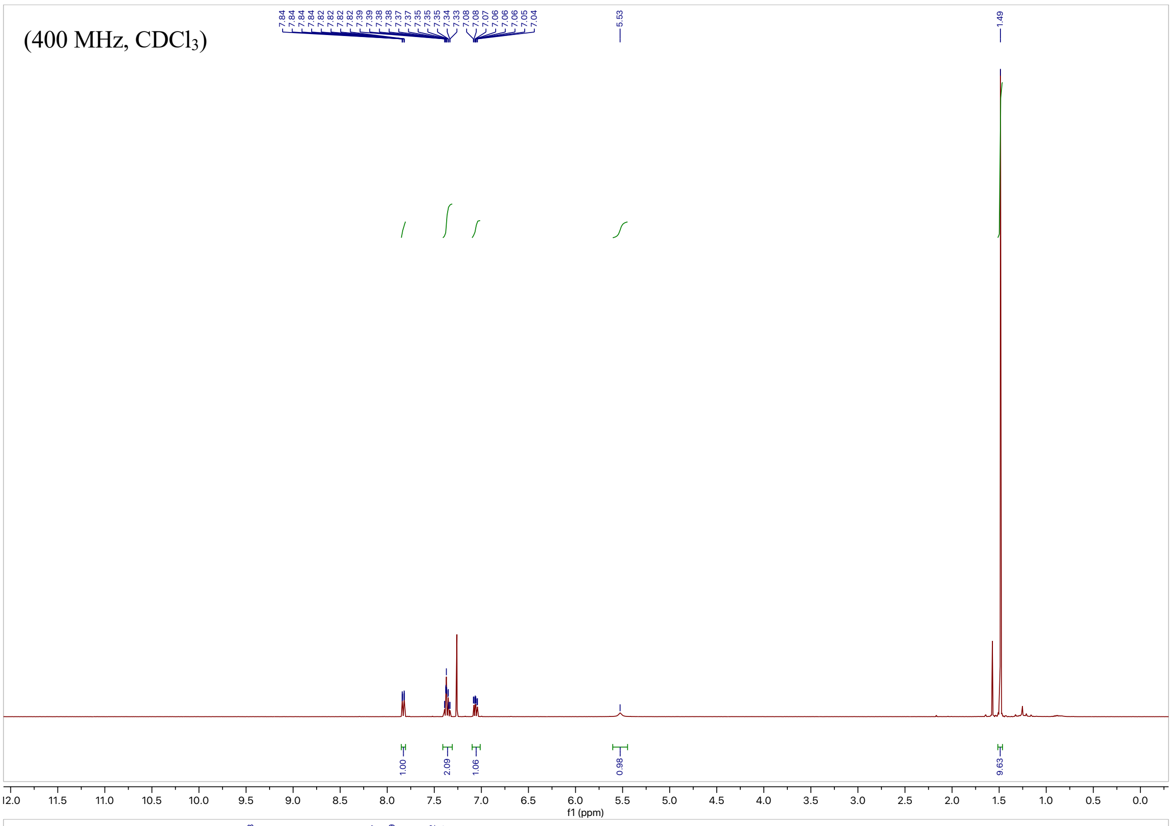

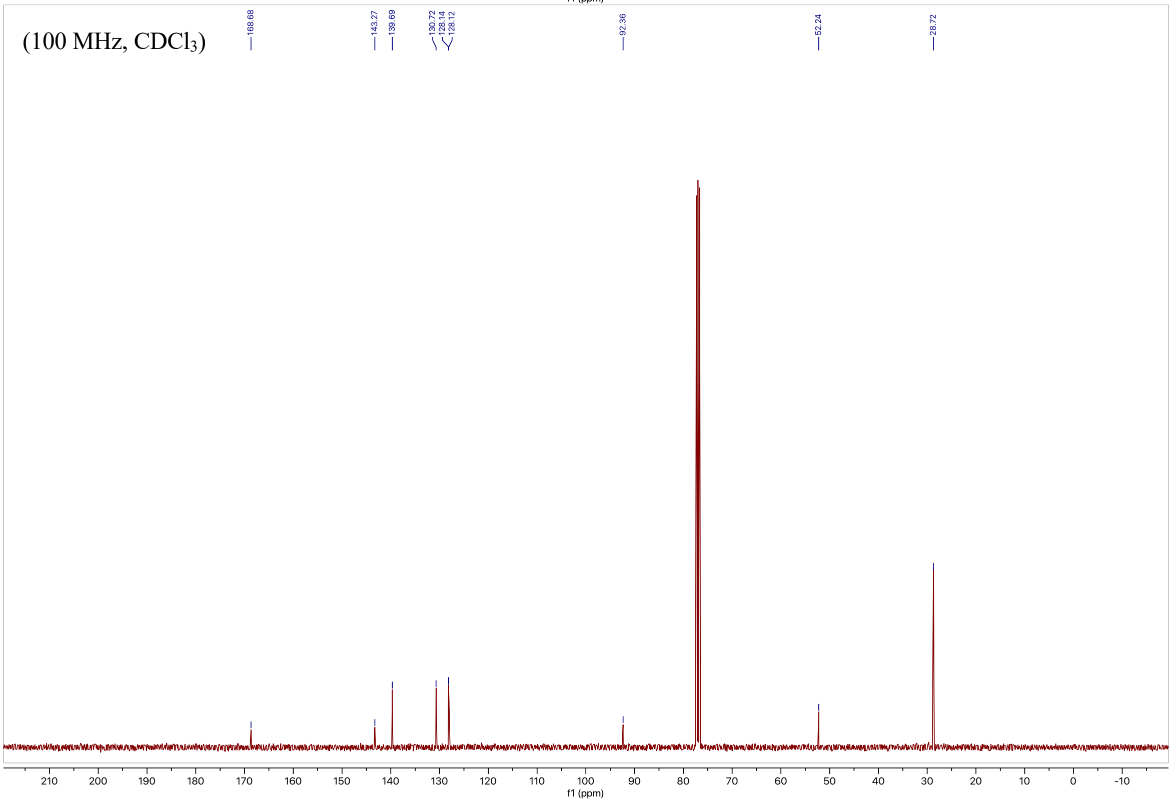

2-Bromo-*N*-(*tert*-butyl)benzamide (2b)

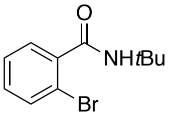

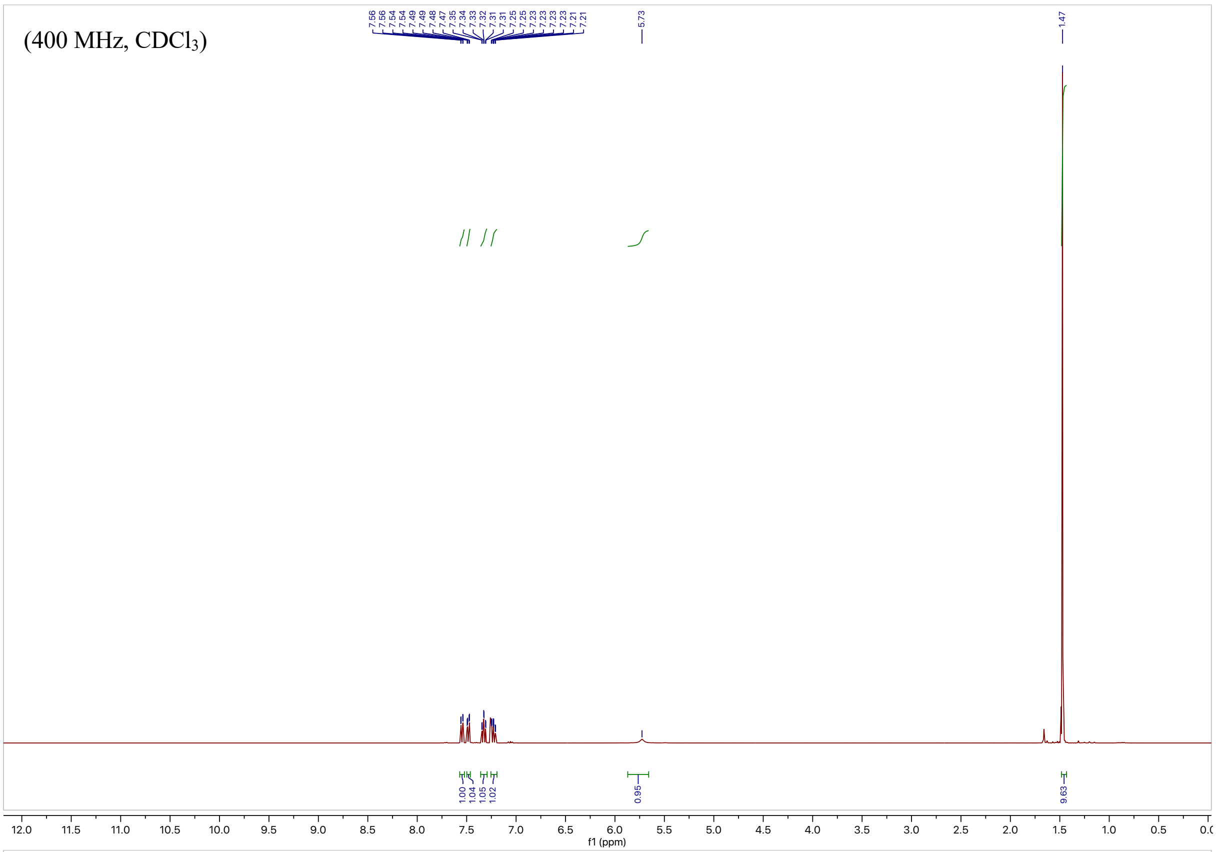

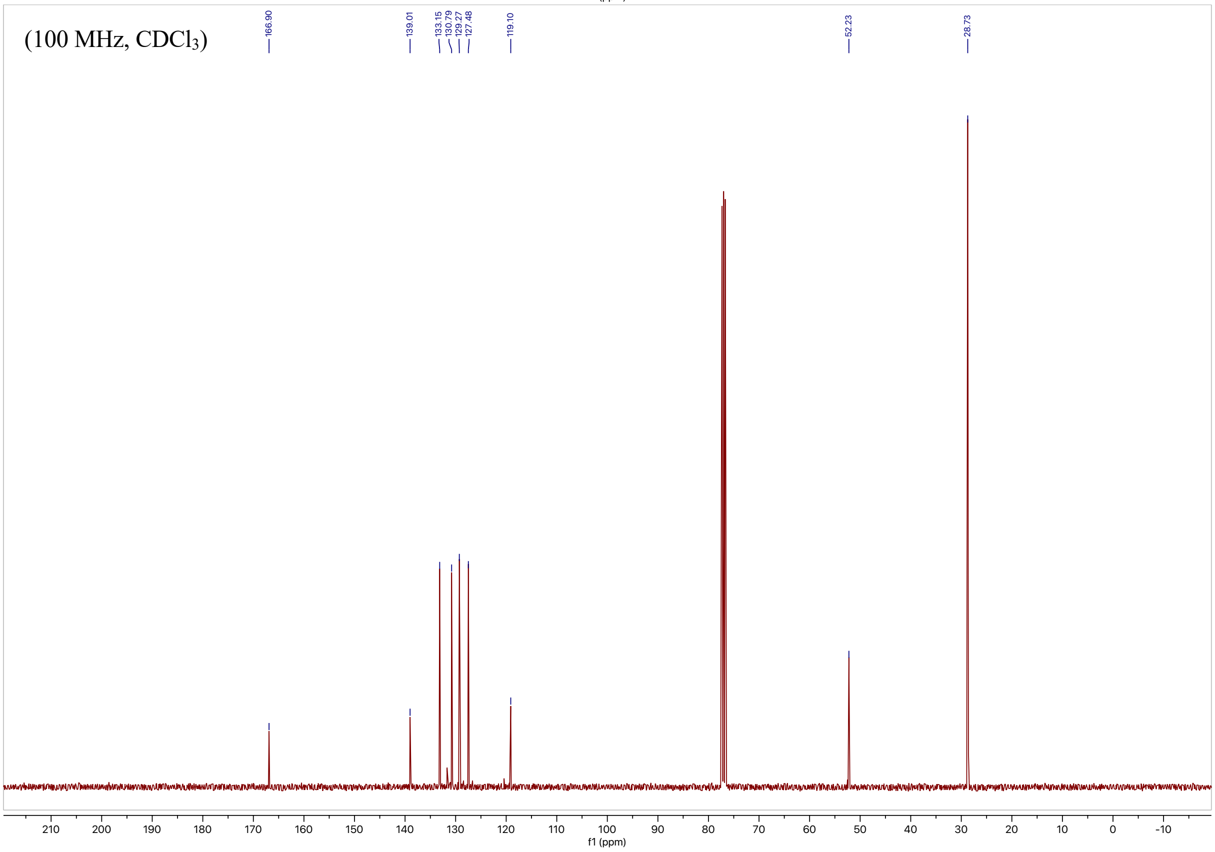

2-Iodo-*N*-isopropylbenzamide (2c)

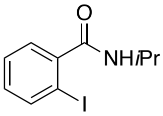

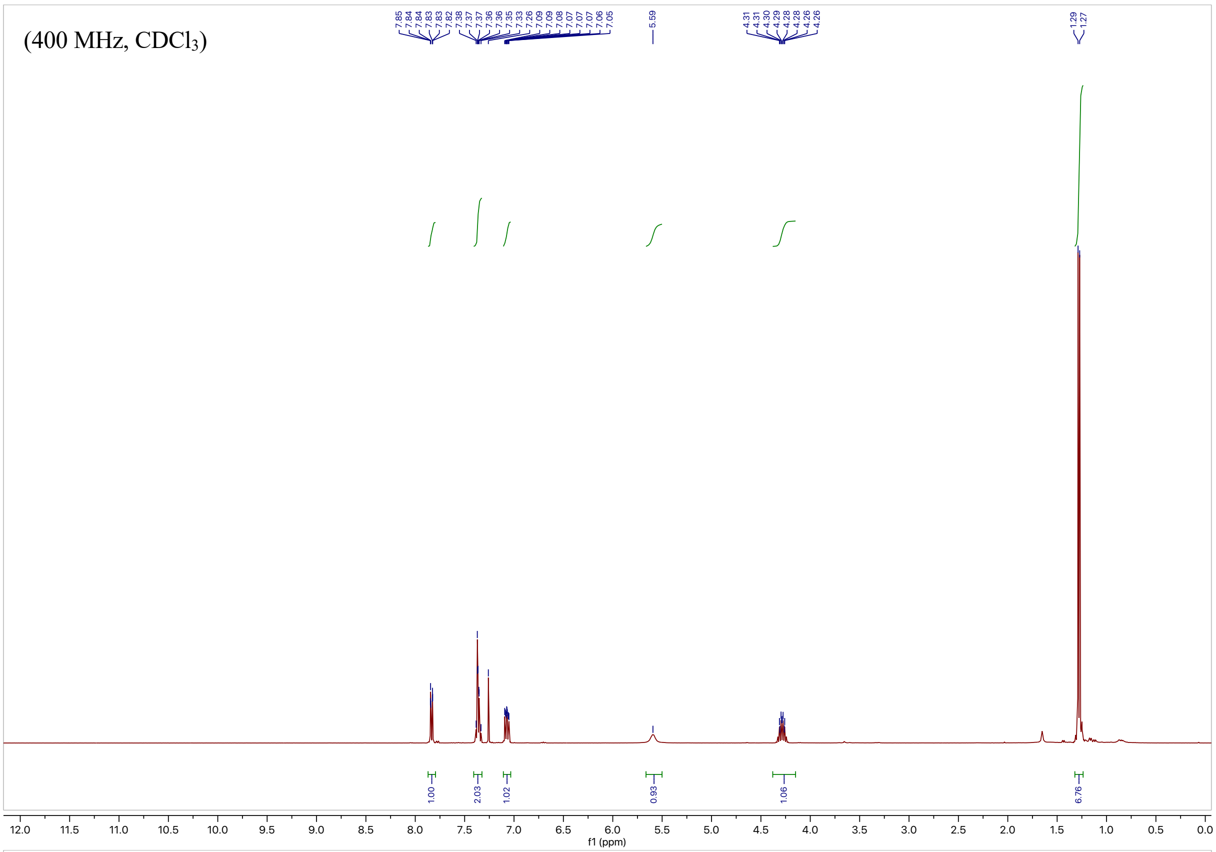

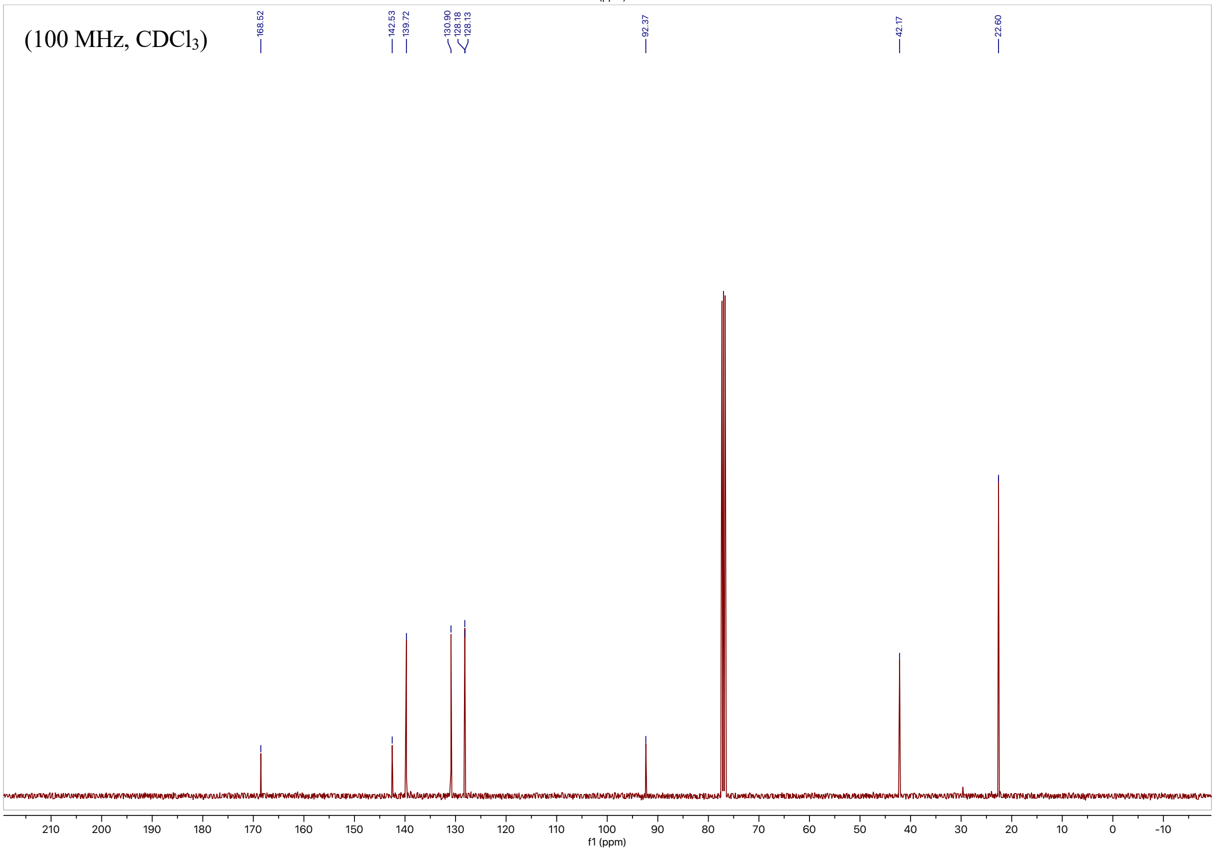

N-Ethyl-2-iodobenzamide (2d)

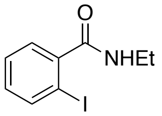

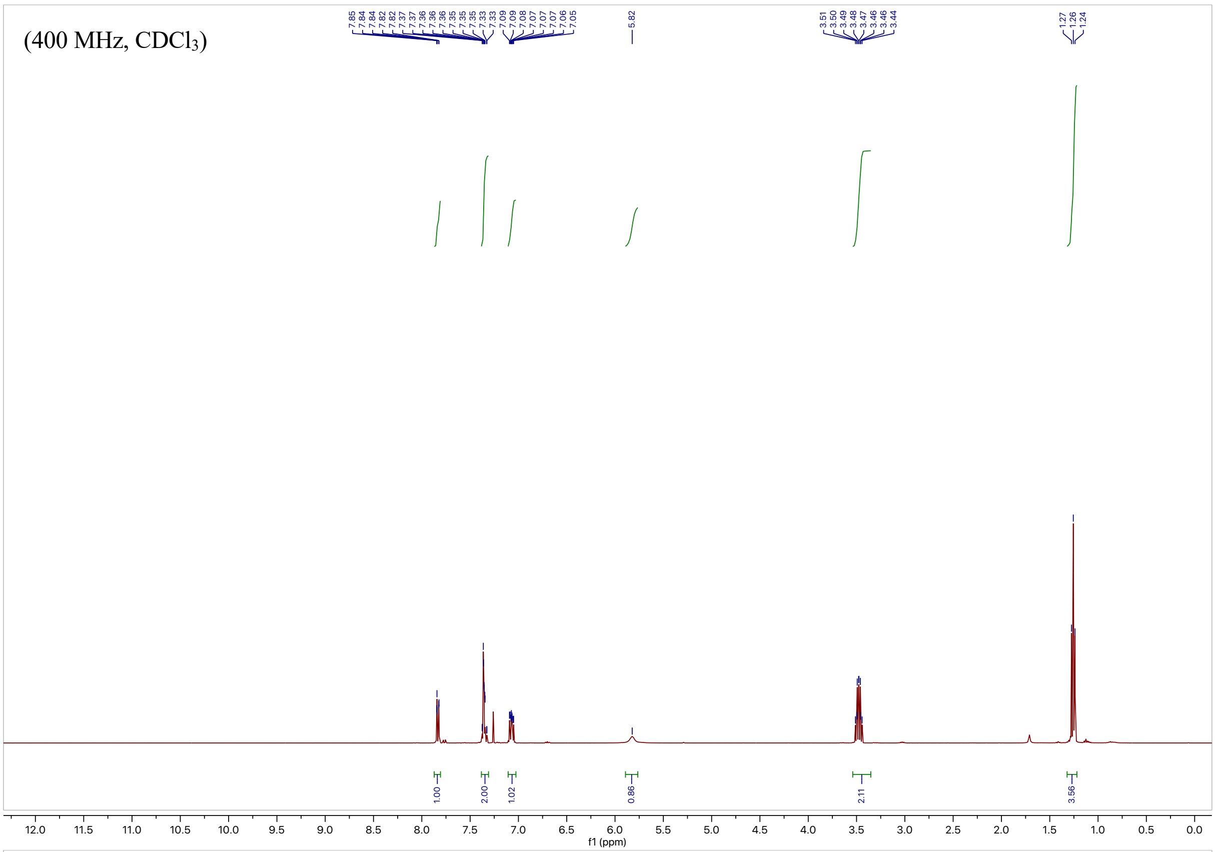

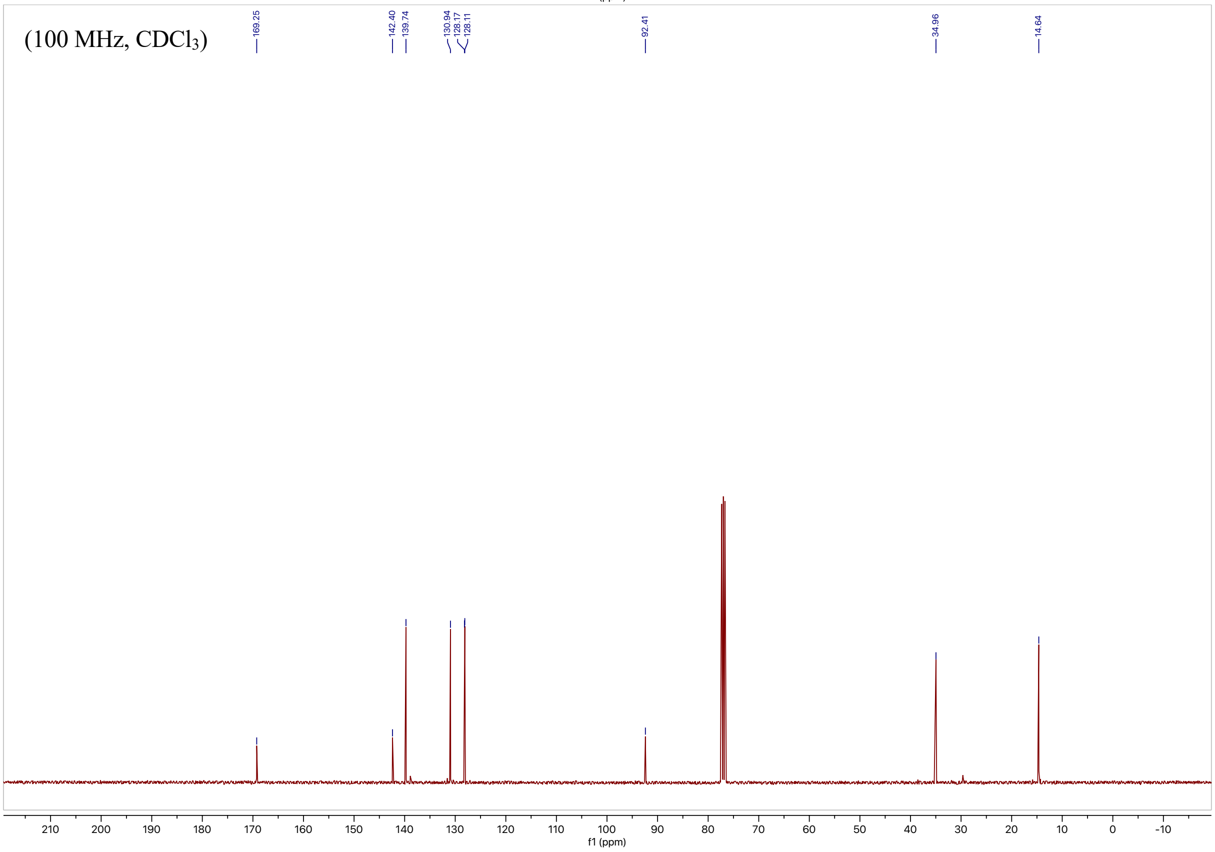

N-Benzyl-2-iodobenzamide (2e)

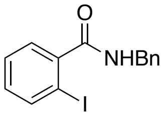

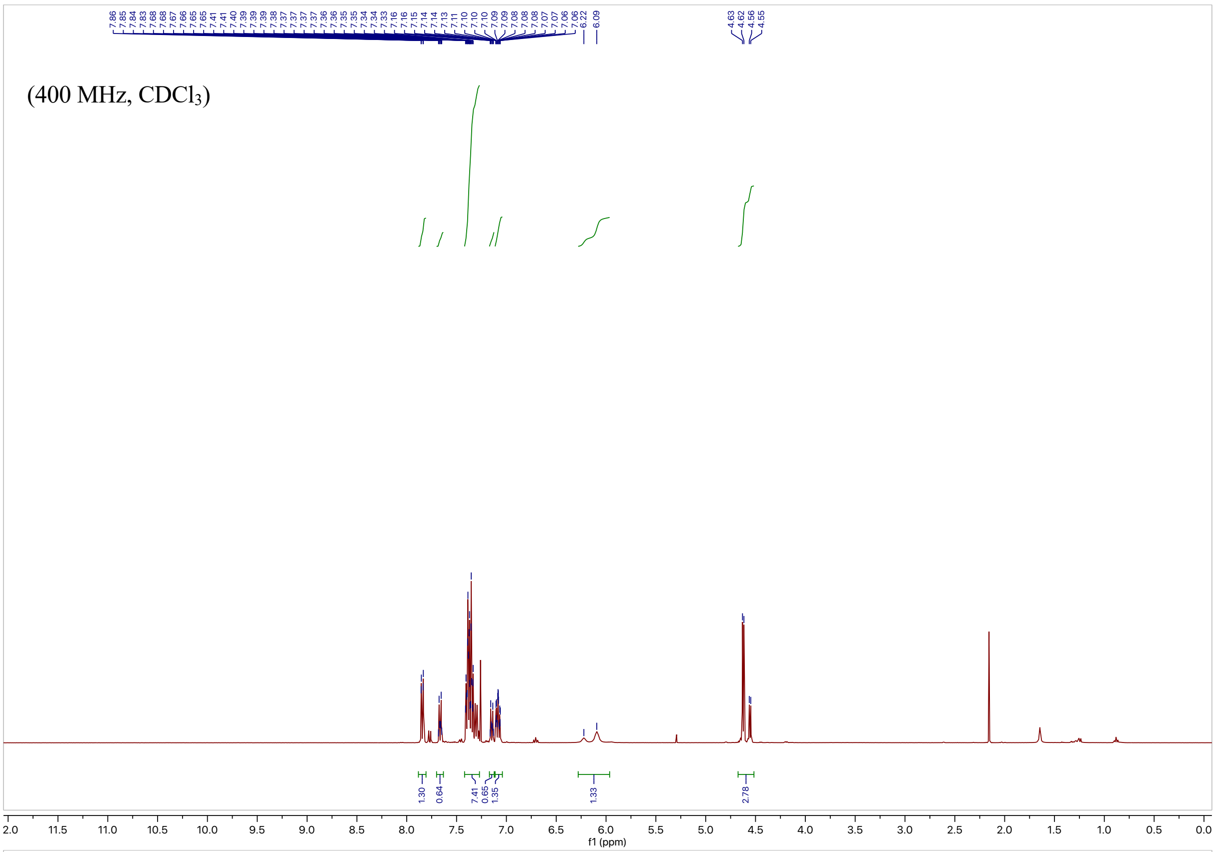

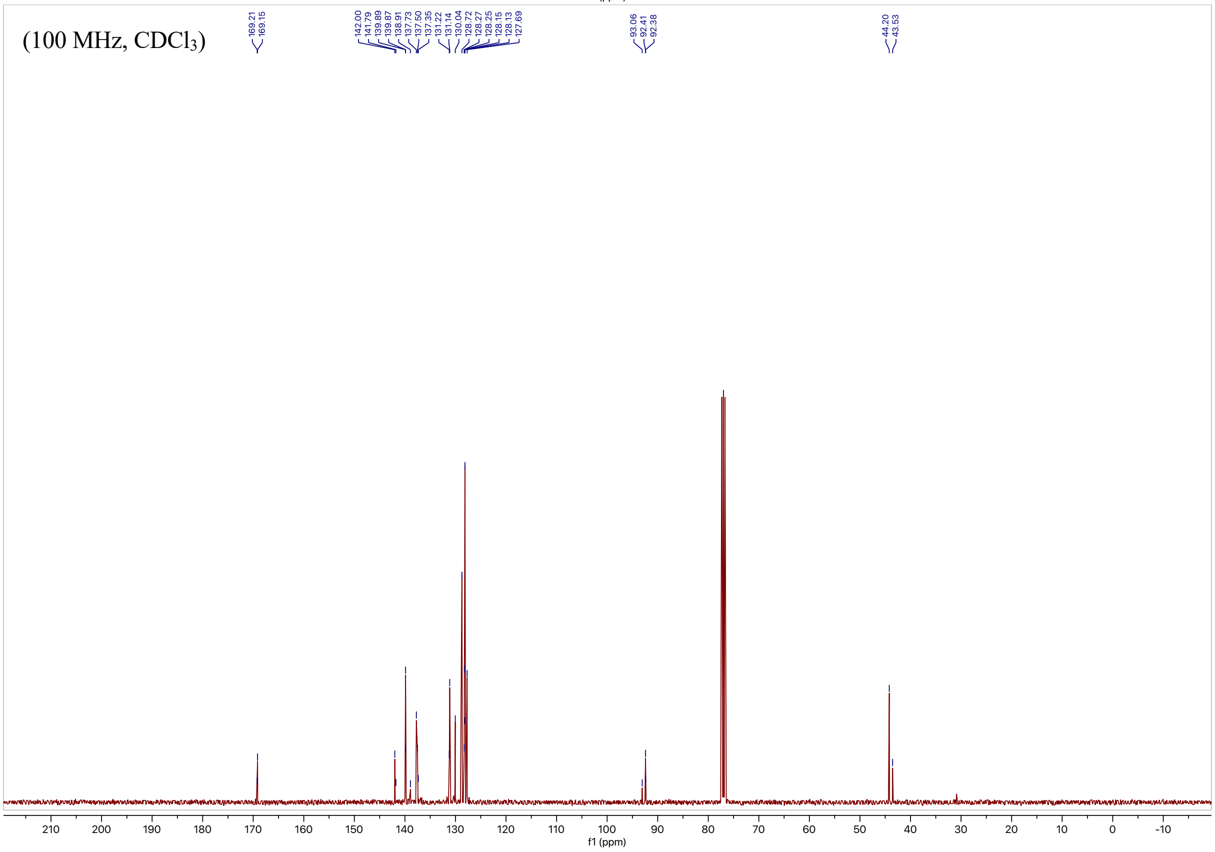

2-Iodo-*N*,*N*-diisopropylbenzamide (2f)

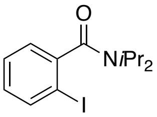

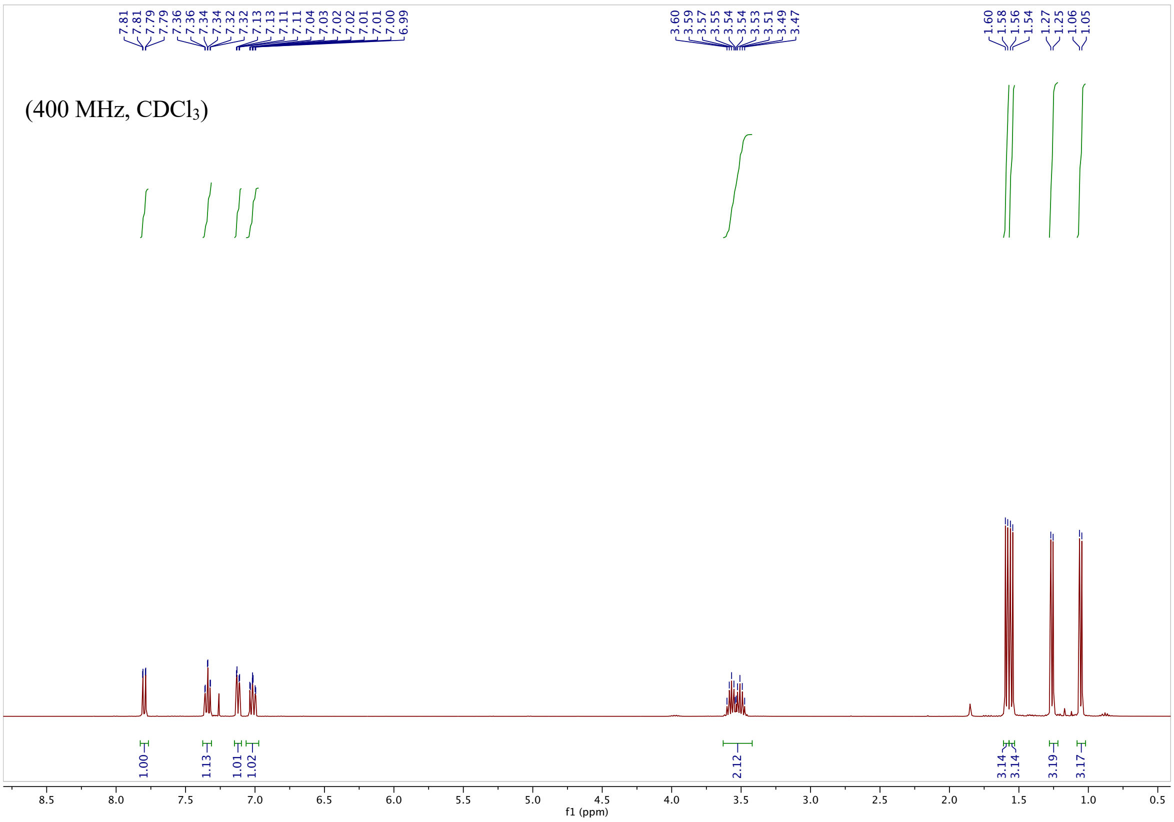

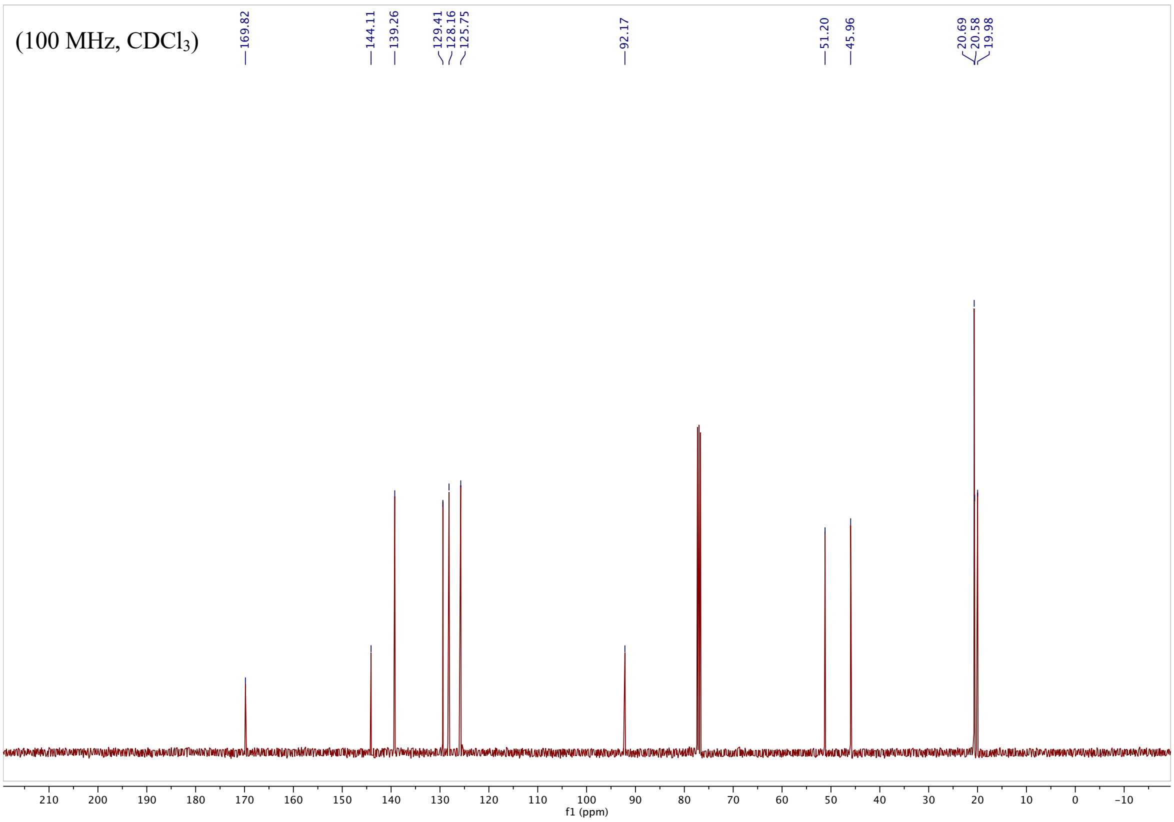

2-Iodo-*N*,*N*-dimethylbenzamide (2g)

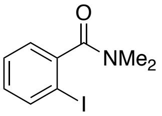

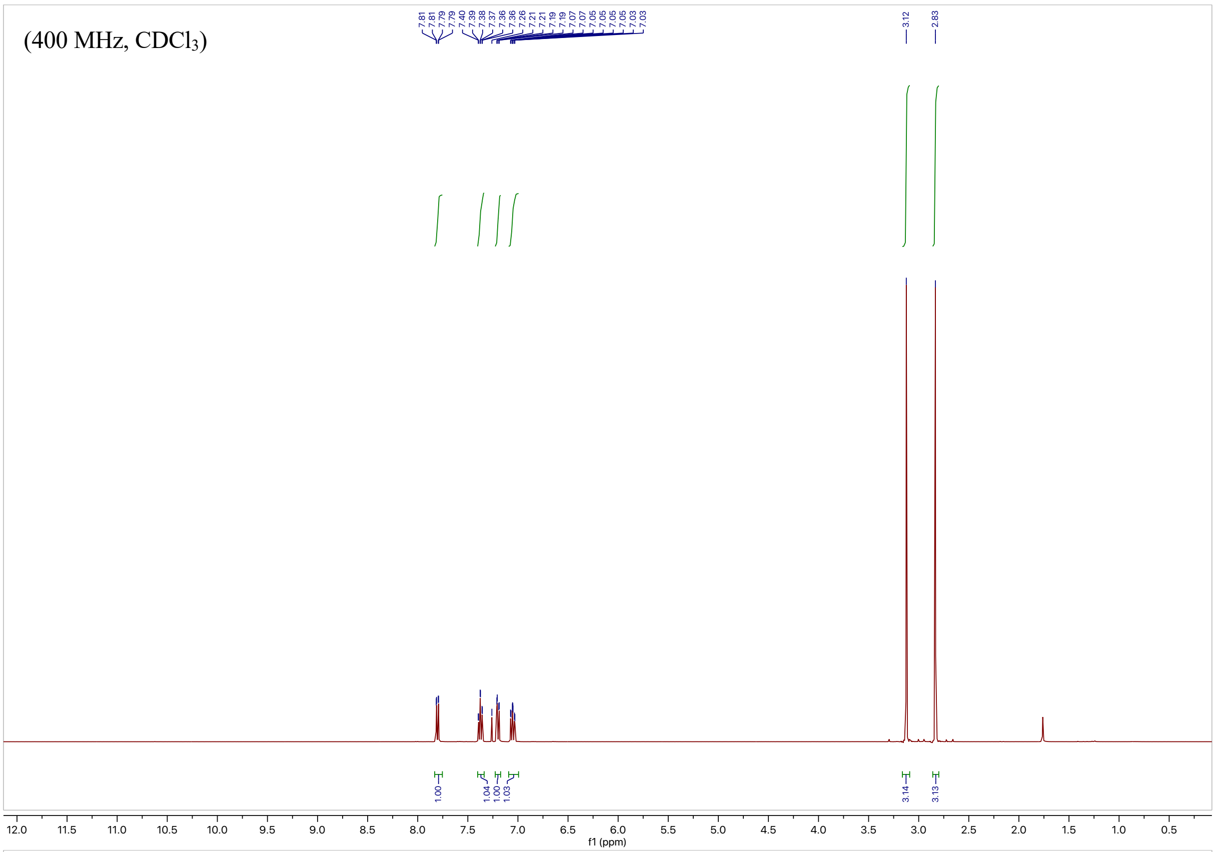

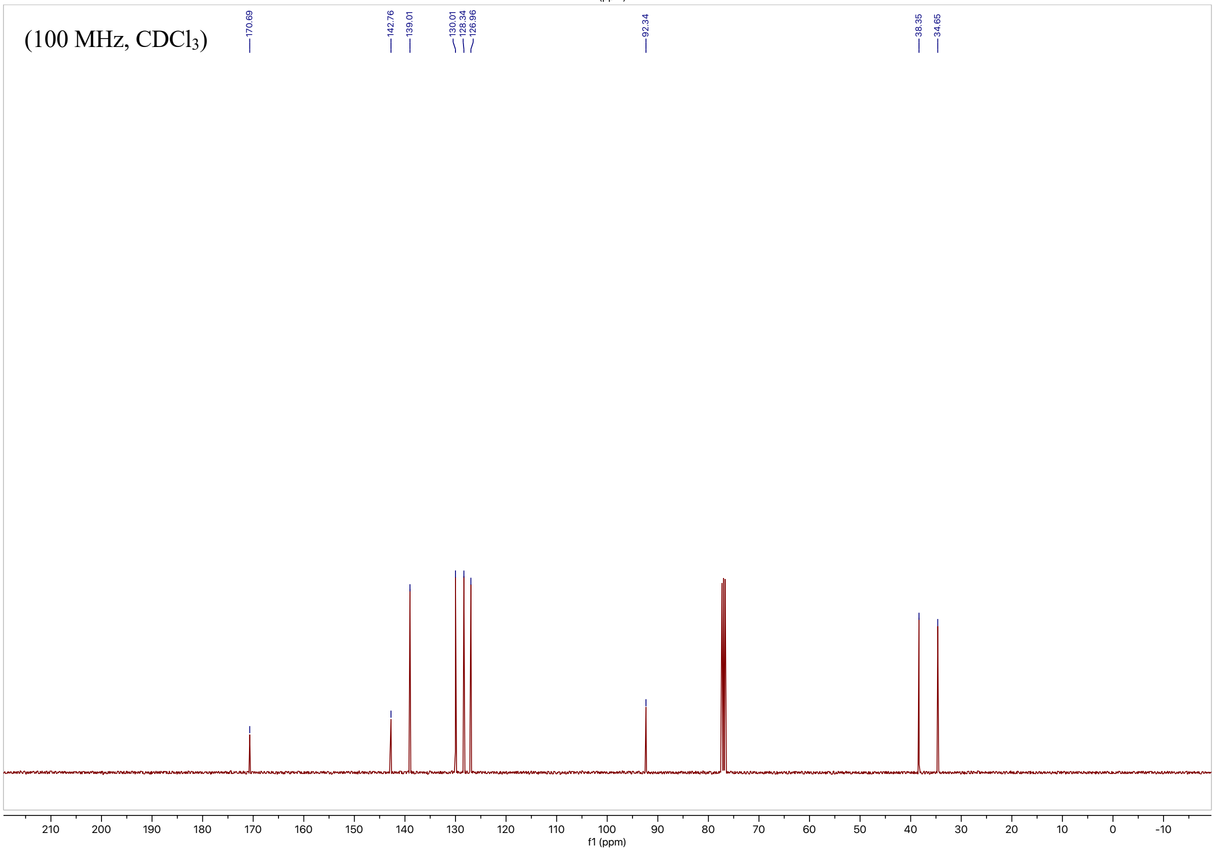

2-Iodobenzamide (2h)

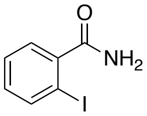

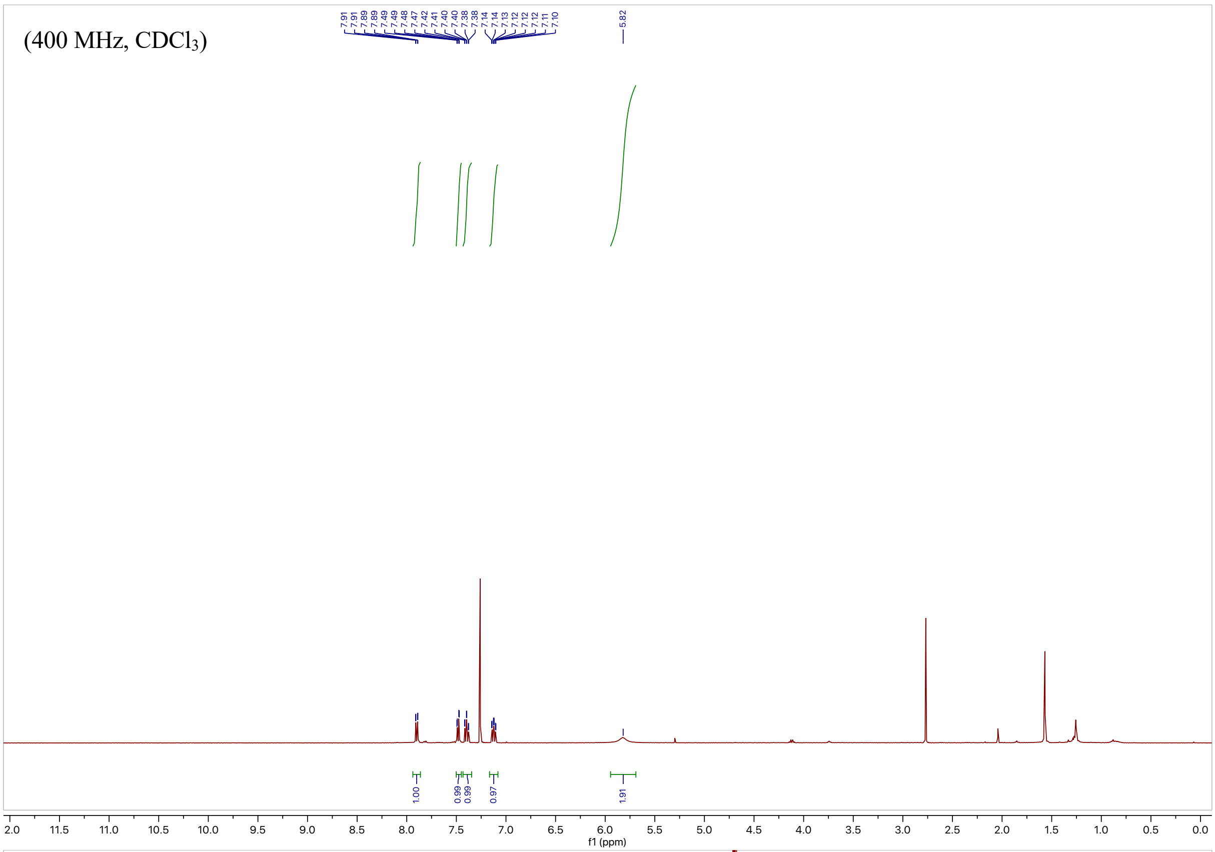

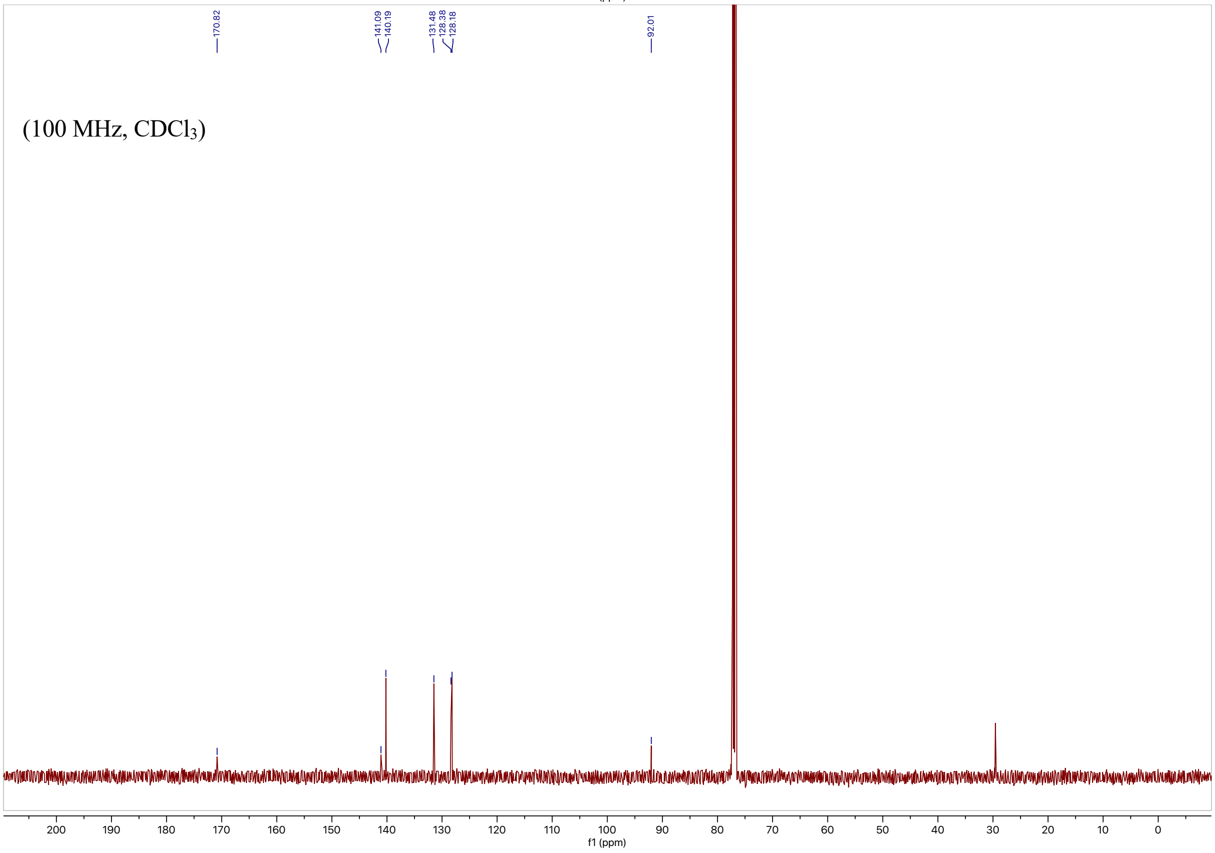

N-(*tert*-Butyl)-2,6-diiodobenzamide (3a)

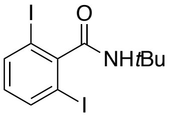

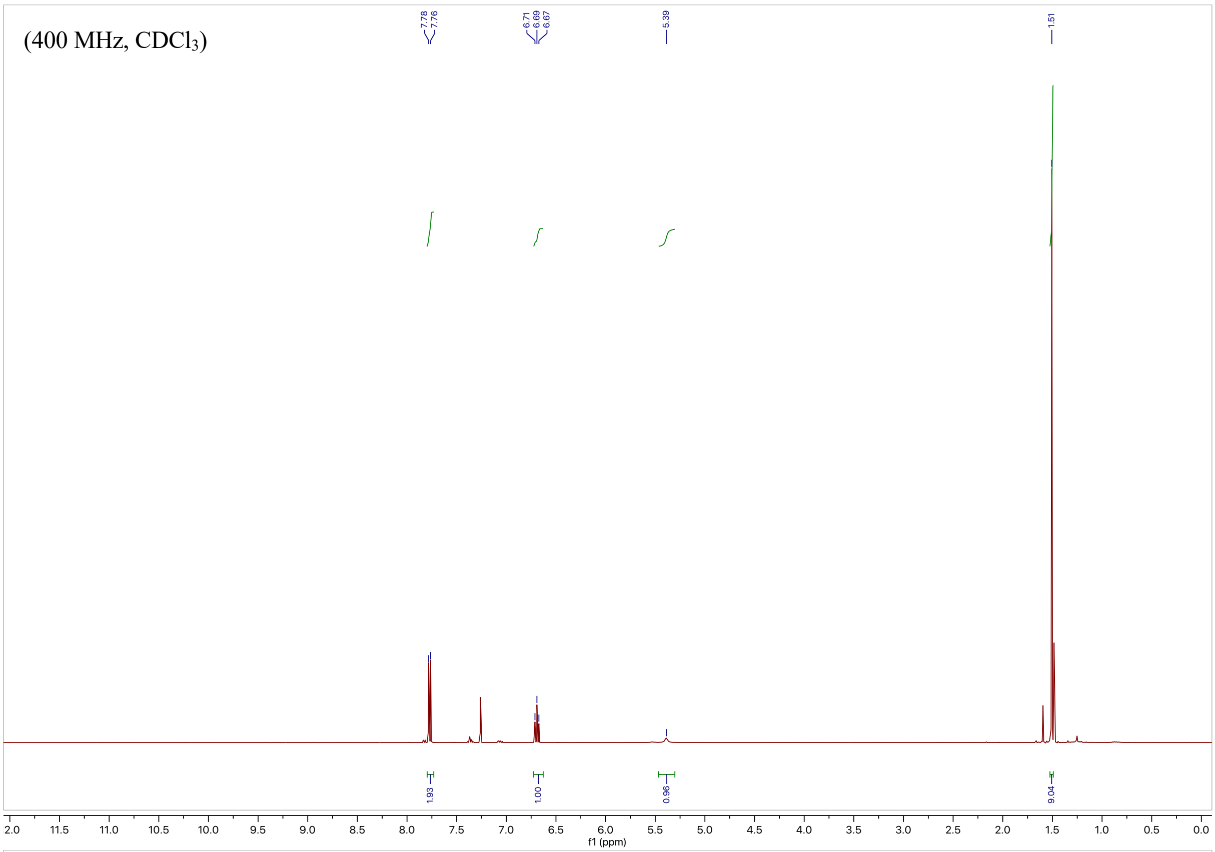

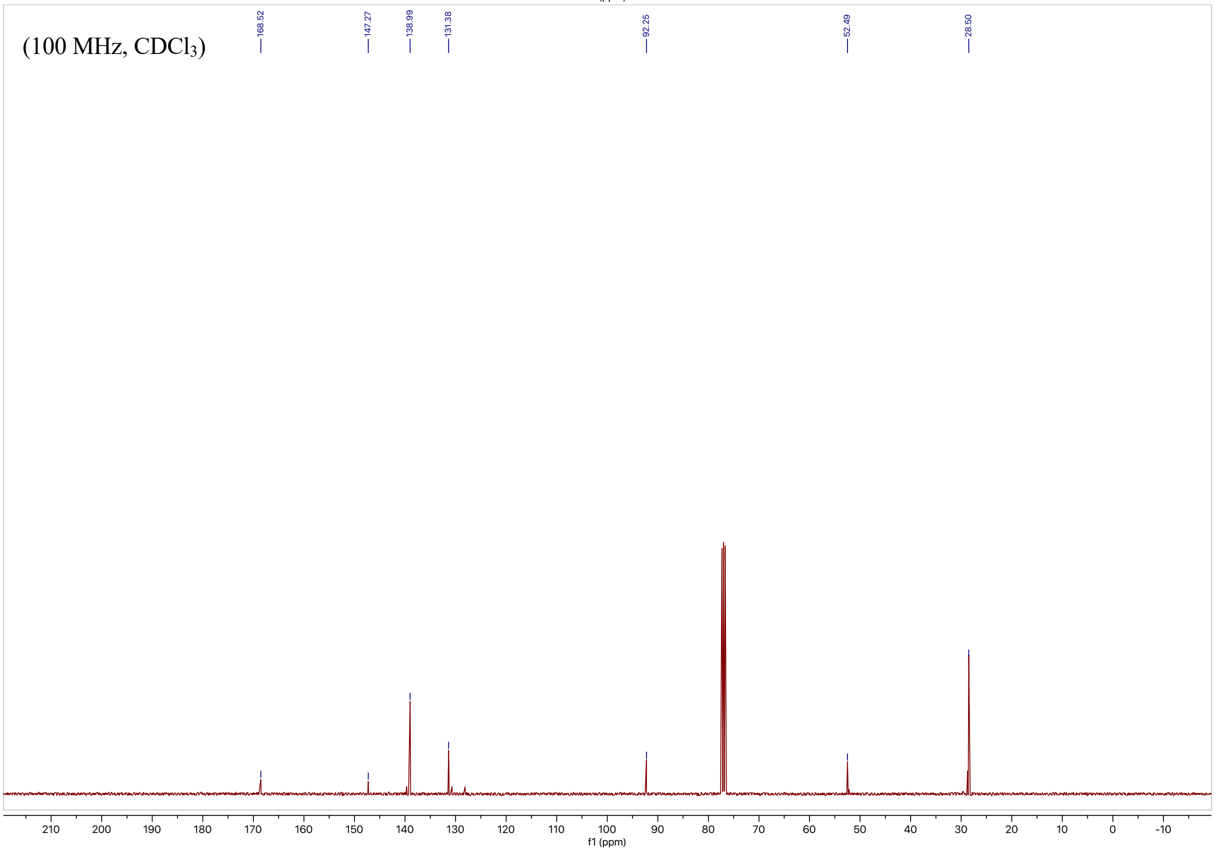

2,6-Diiodo-*N*-isopropylbenzamide (3c)

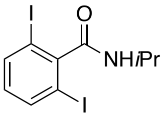

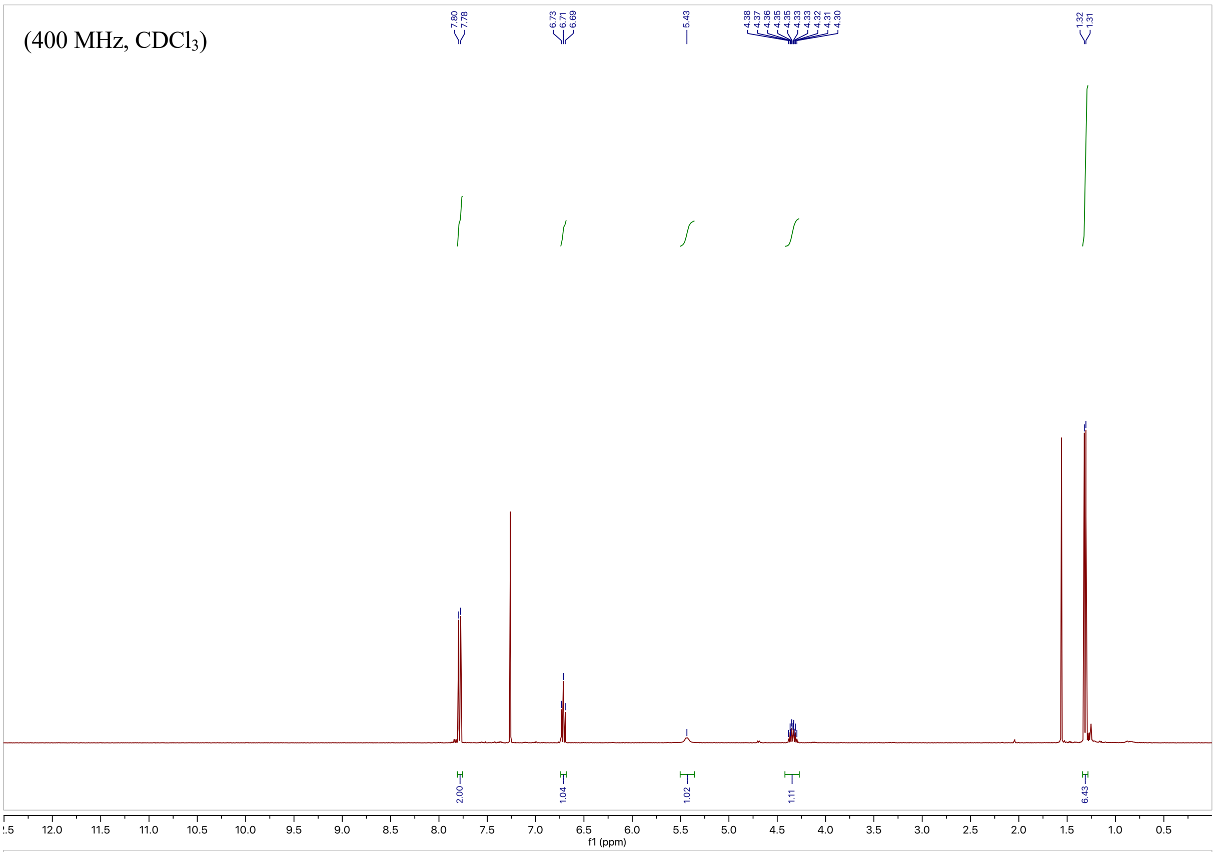

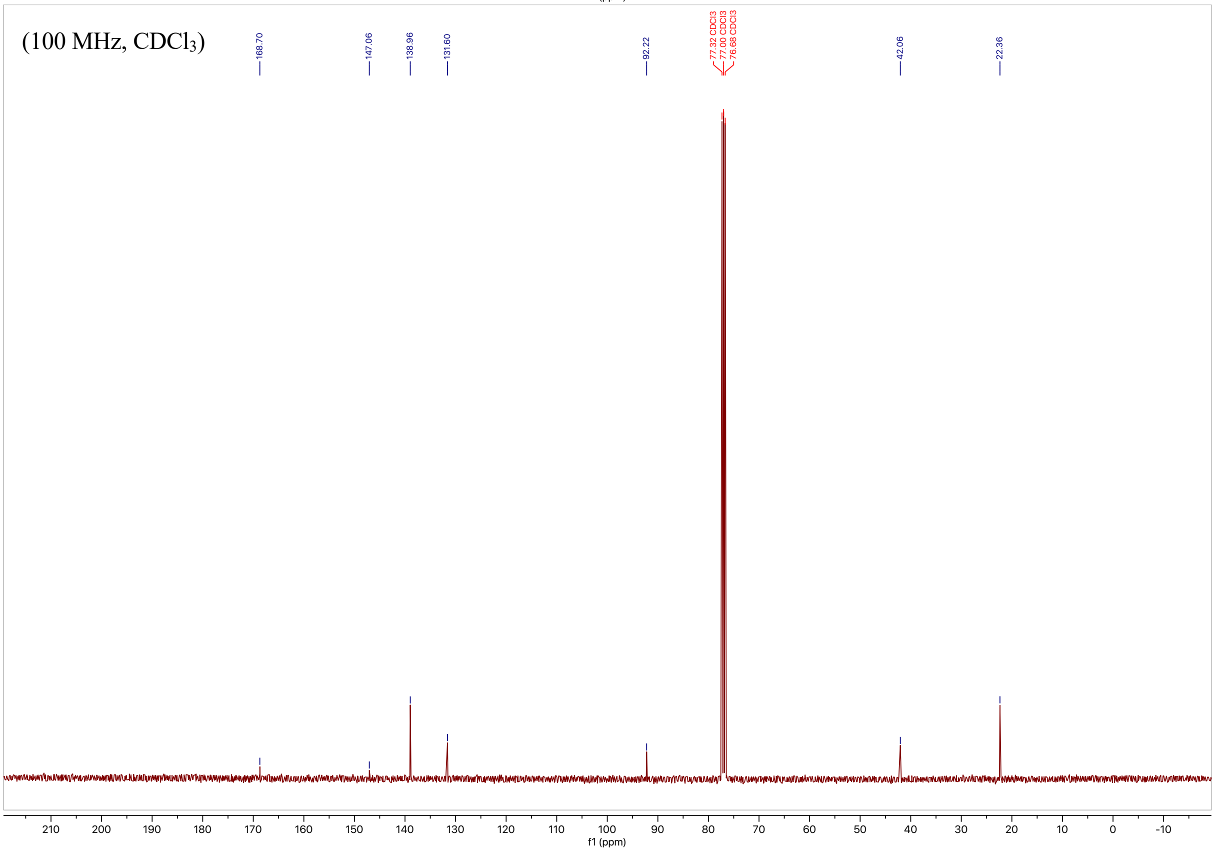

N-Ethyl-2,6-diiodobenzamide (3d)

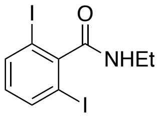

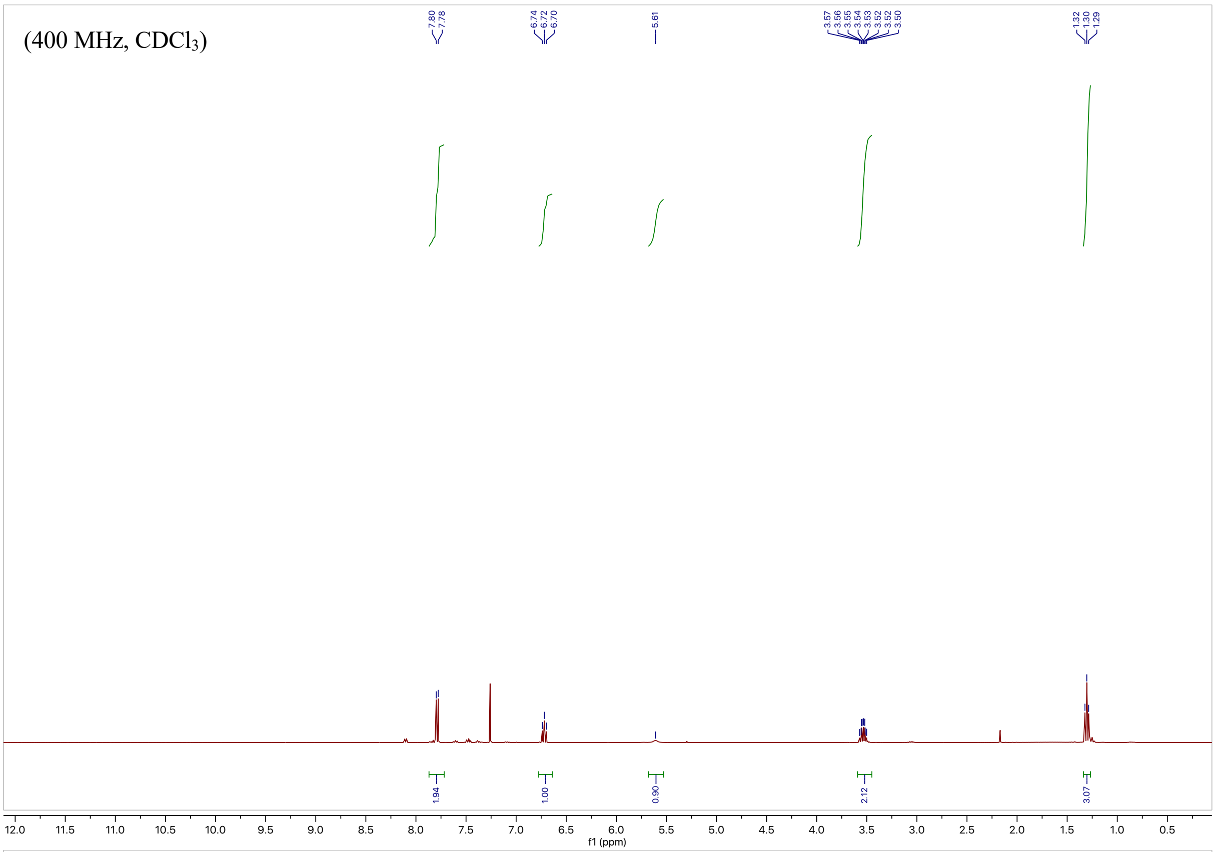

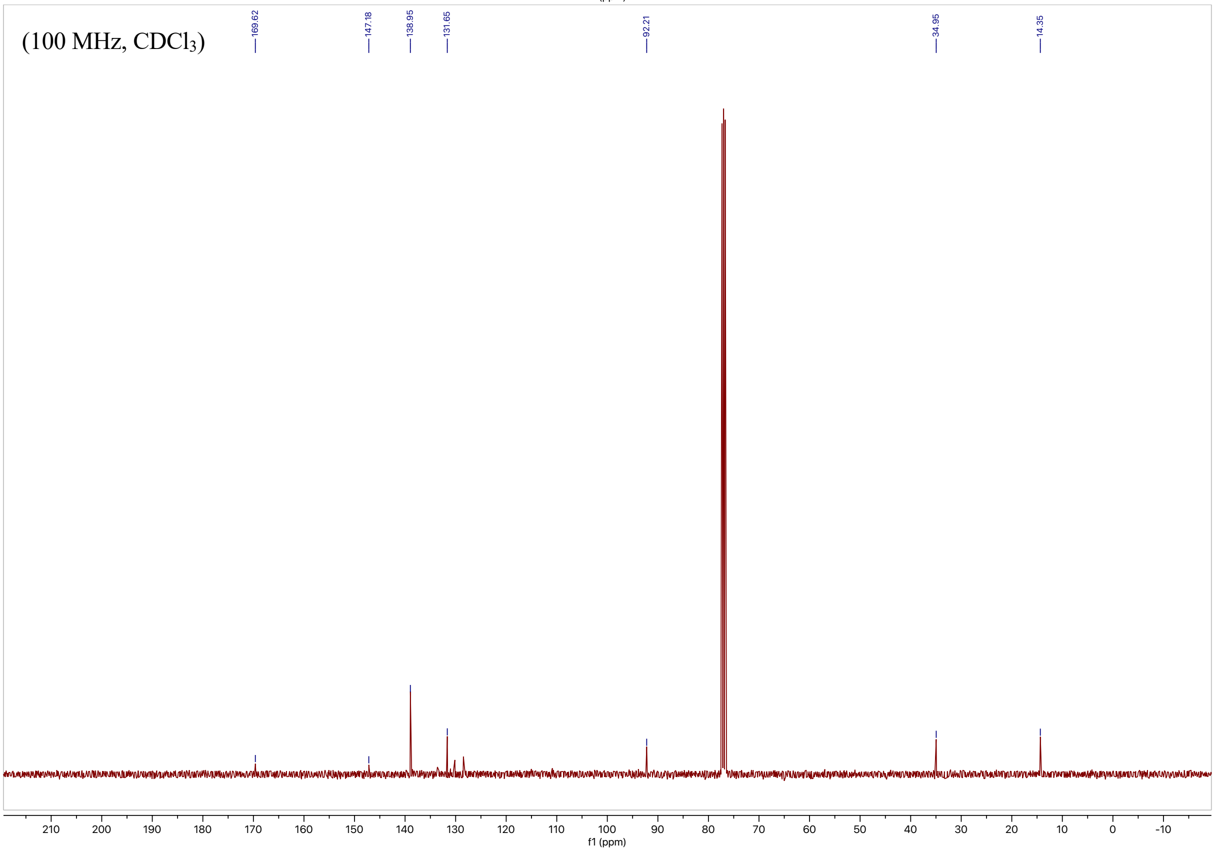

2-Iodo-*N*-methoxy-*N*-methylbenzamide (5a)

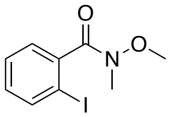

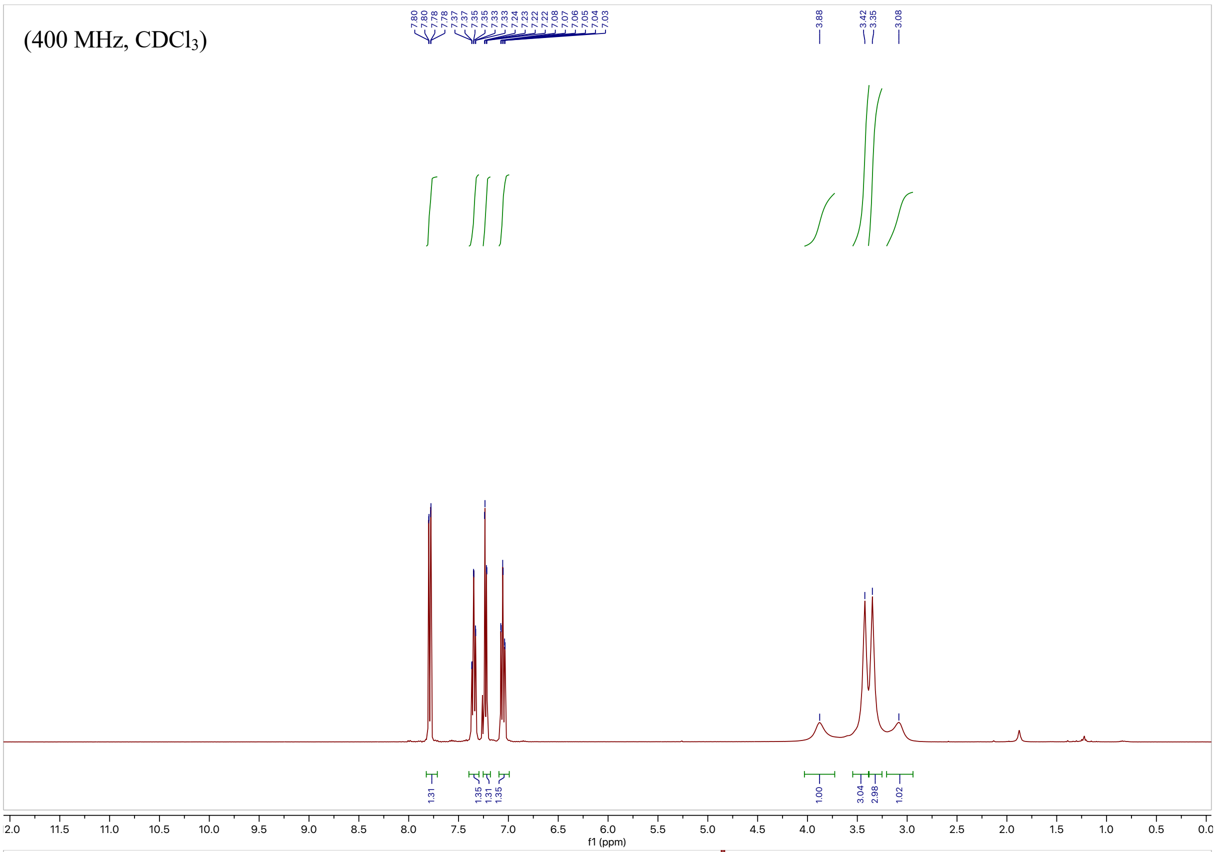

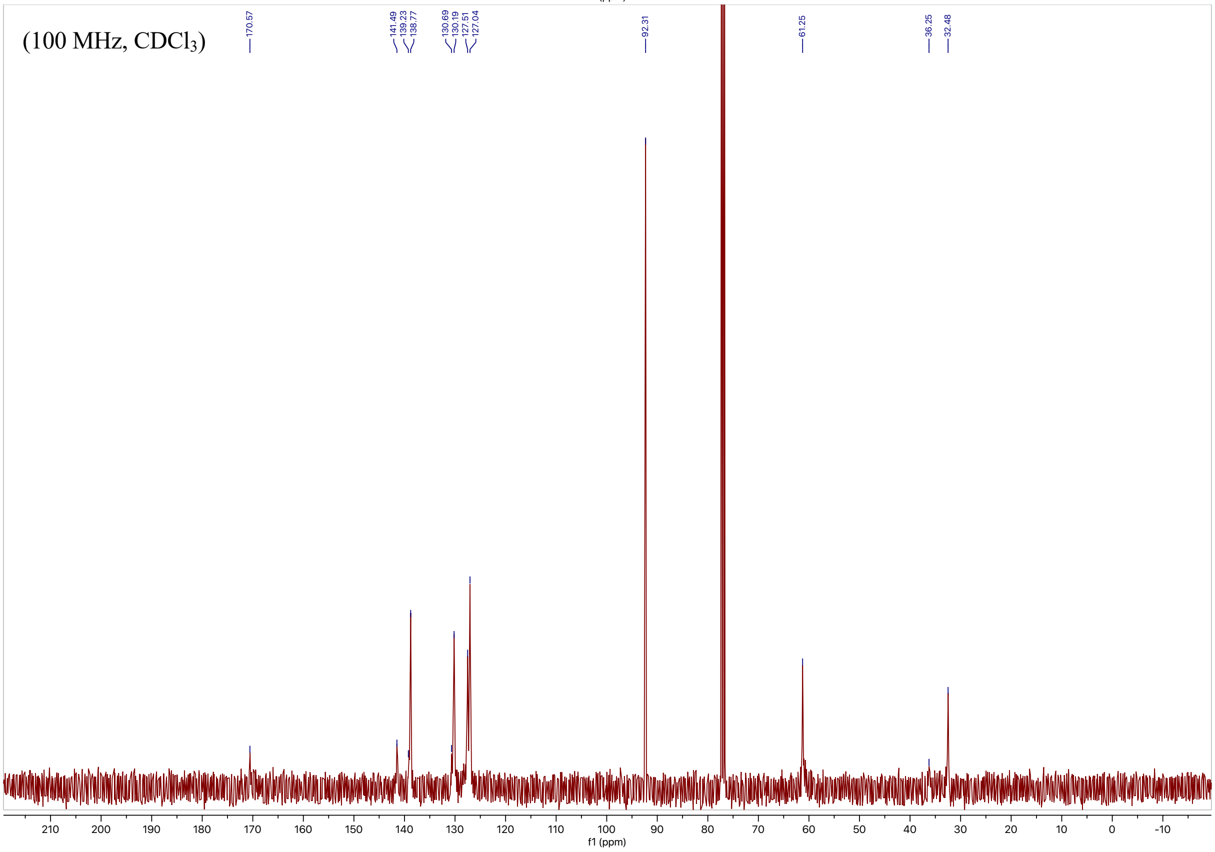

2-Fluoro-6-iodo-*N*-methoxy-*N*-methylbenzamide (5b)

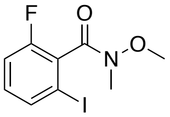

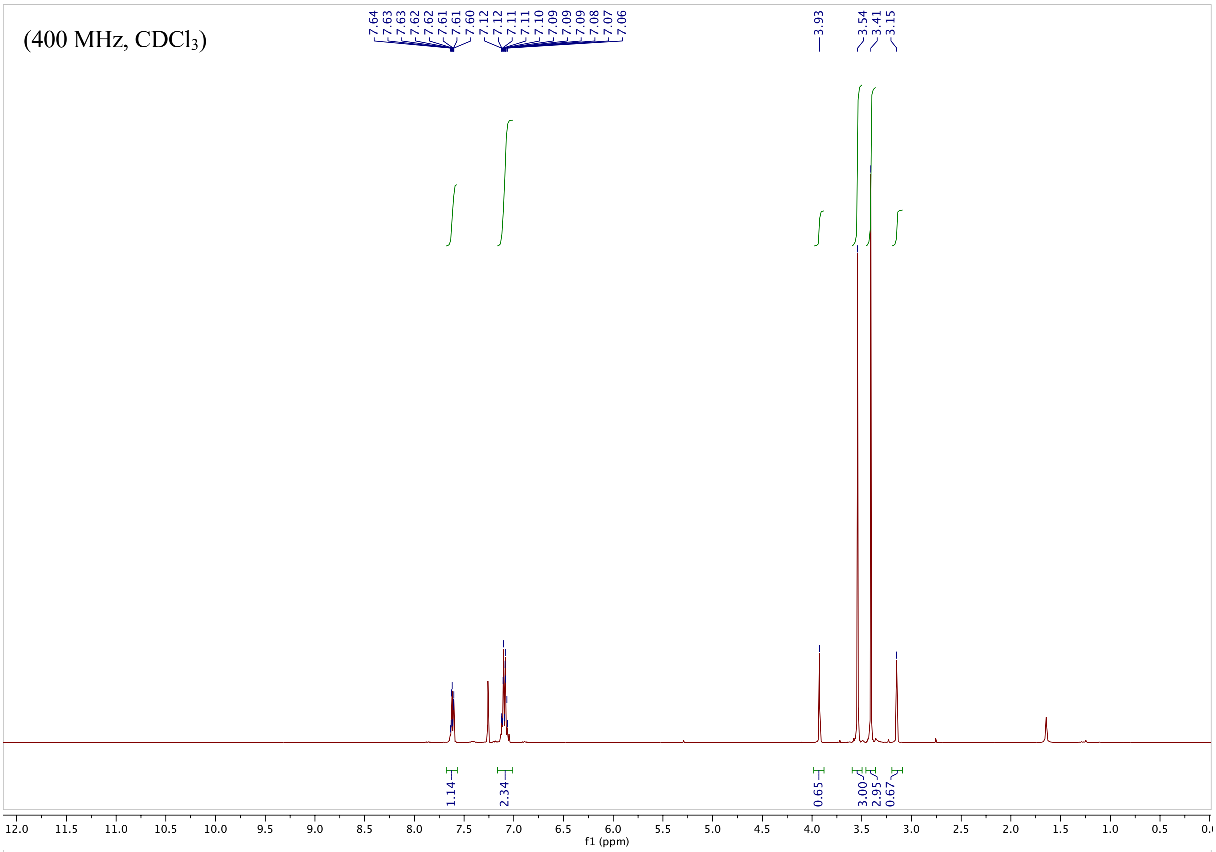

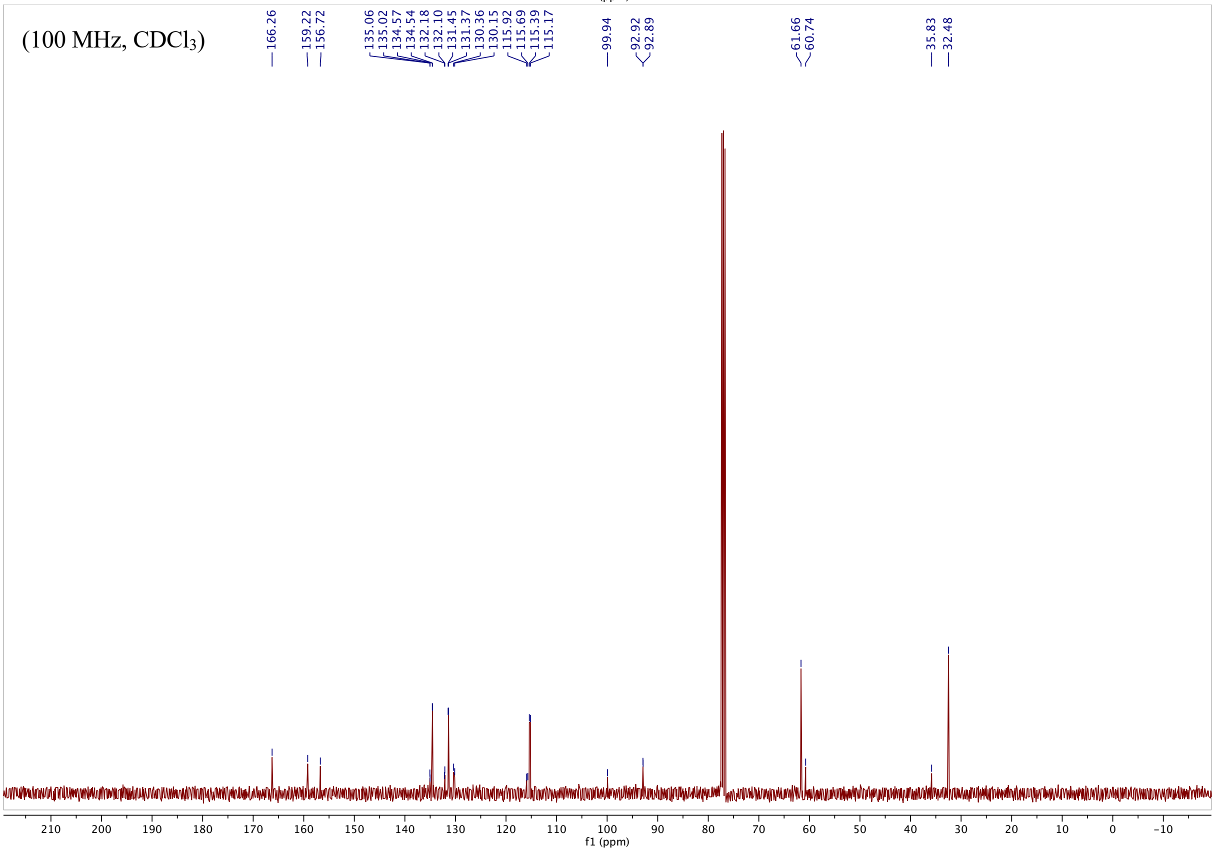

4-Bromo-2-iodo-*N*-methoxy-*N*-methylbenzamide (5c)

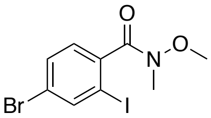

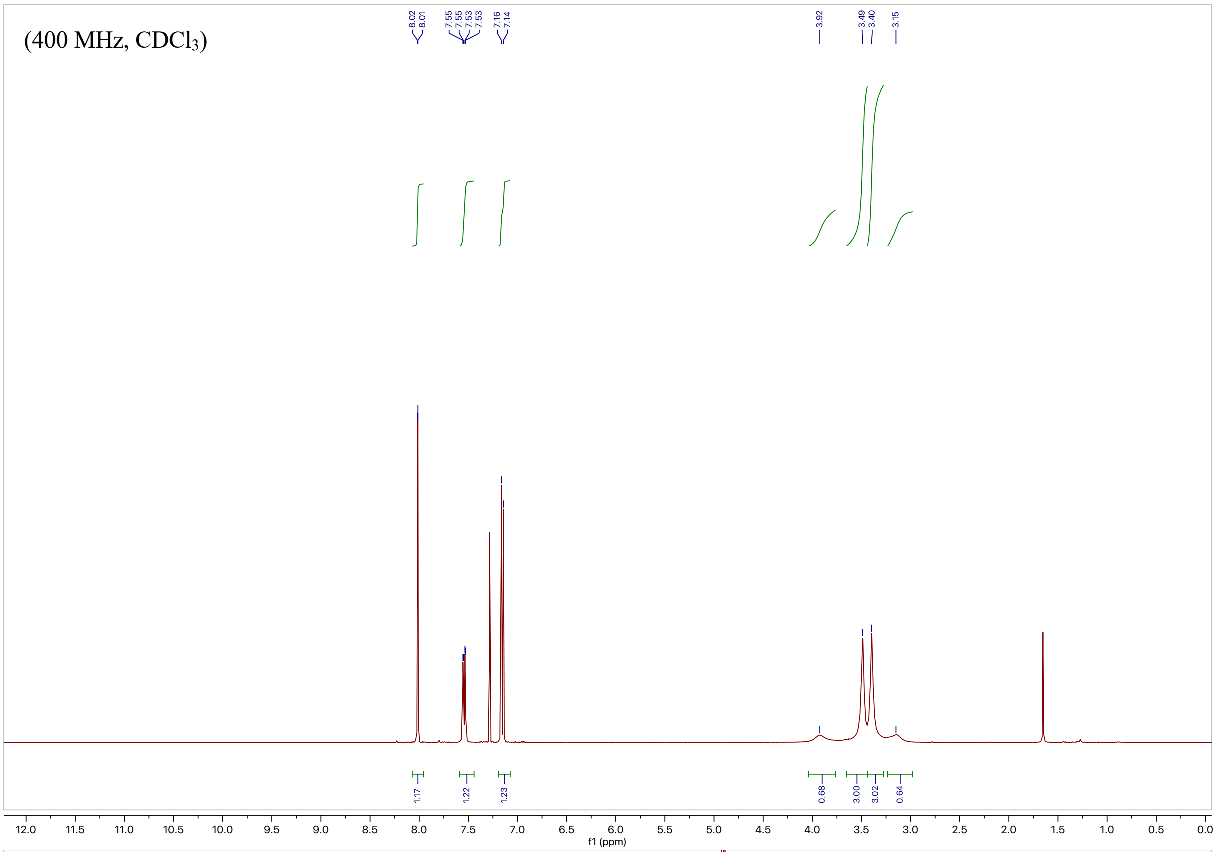

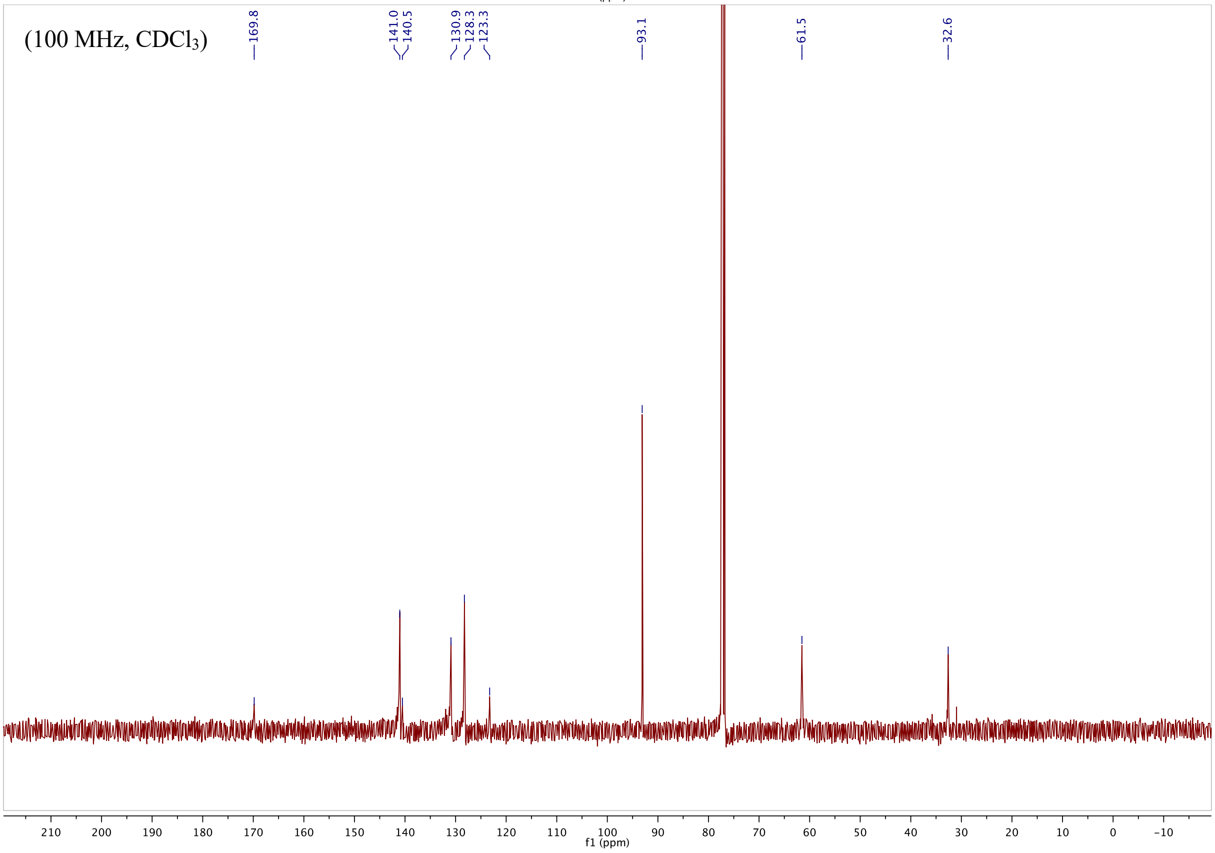

4-Fluoro-2-iodo-*N*-methoxy-*N*-methylbenzamide (5d)

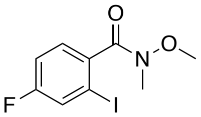

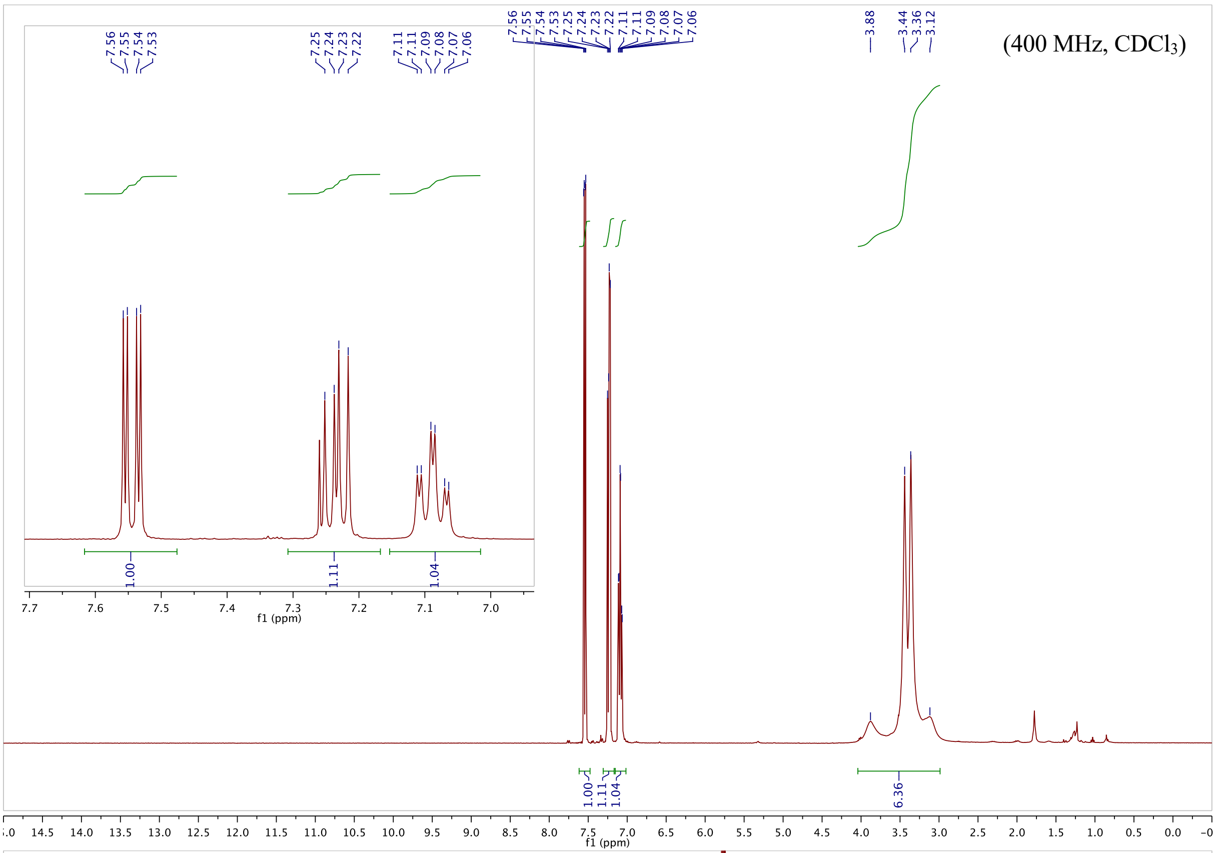

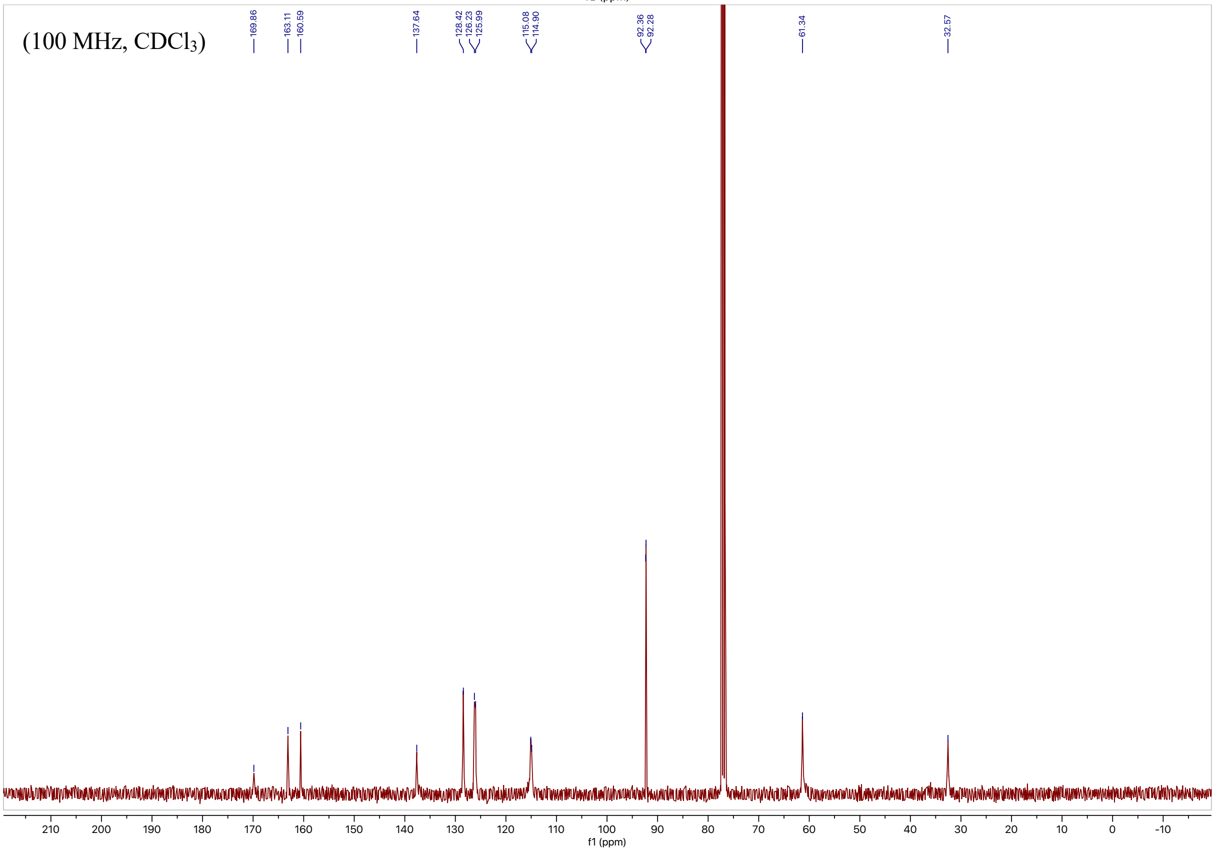

2-Iodo-*N*-methoxy-*N*,4-dimethylbenzamide (5e)

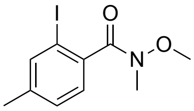

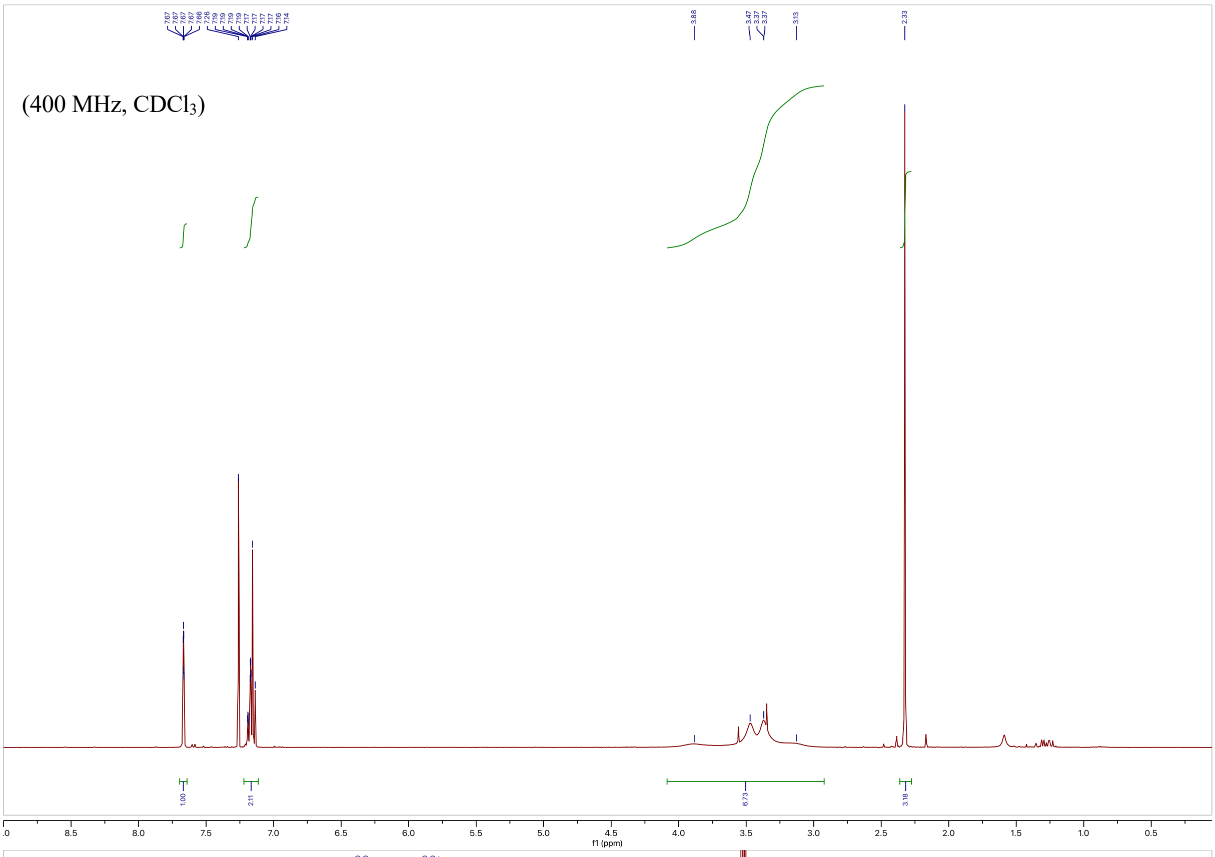

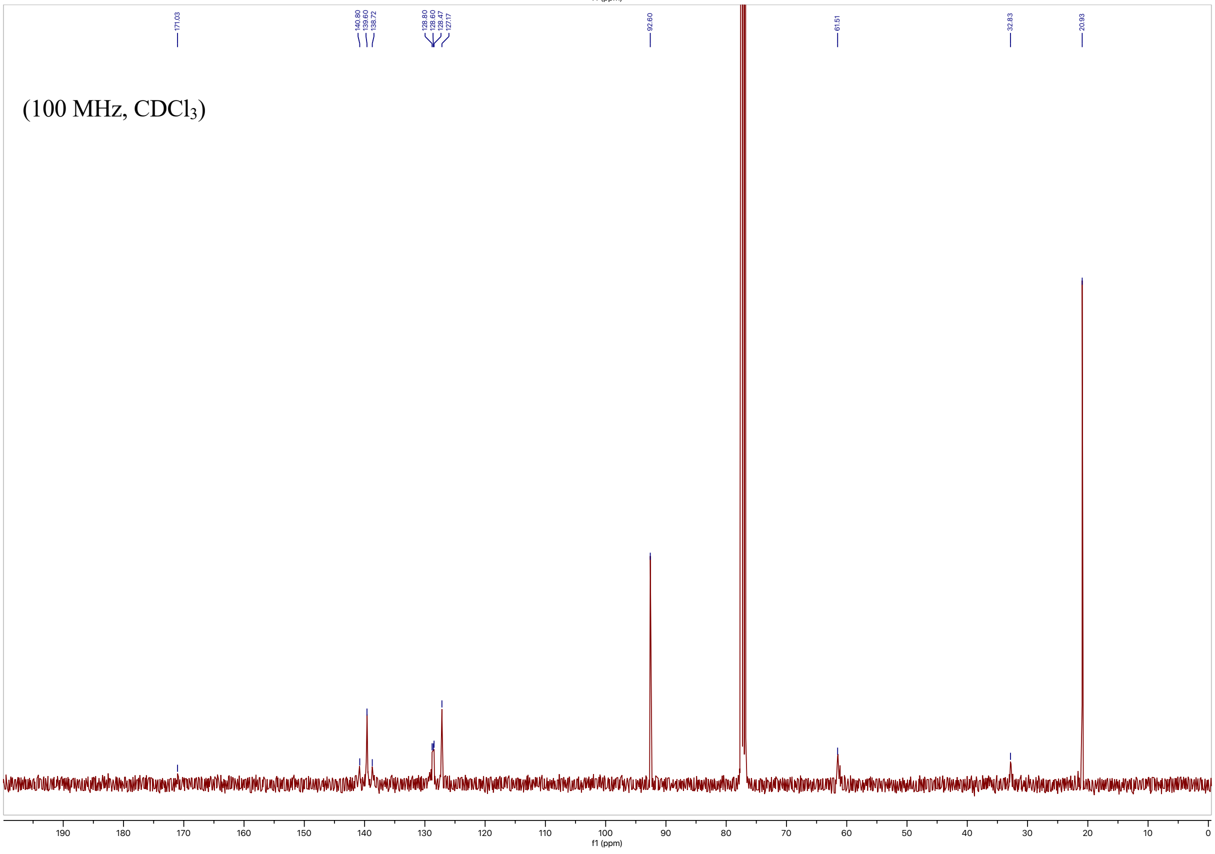

4-(Hydroxymethyl)-2-iodo-*N*-methoxy-*N*-methylbenzamide (5f)

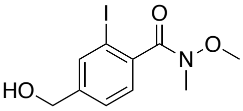

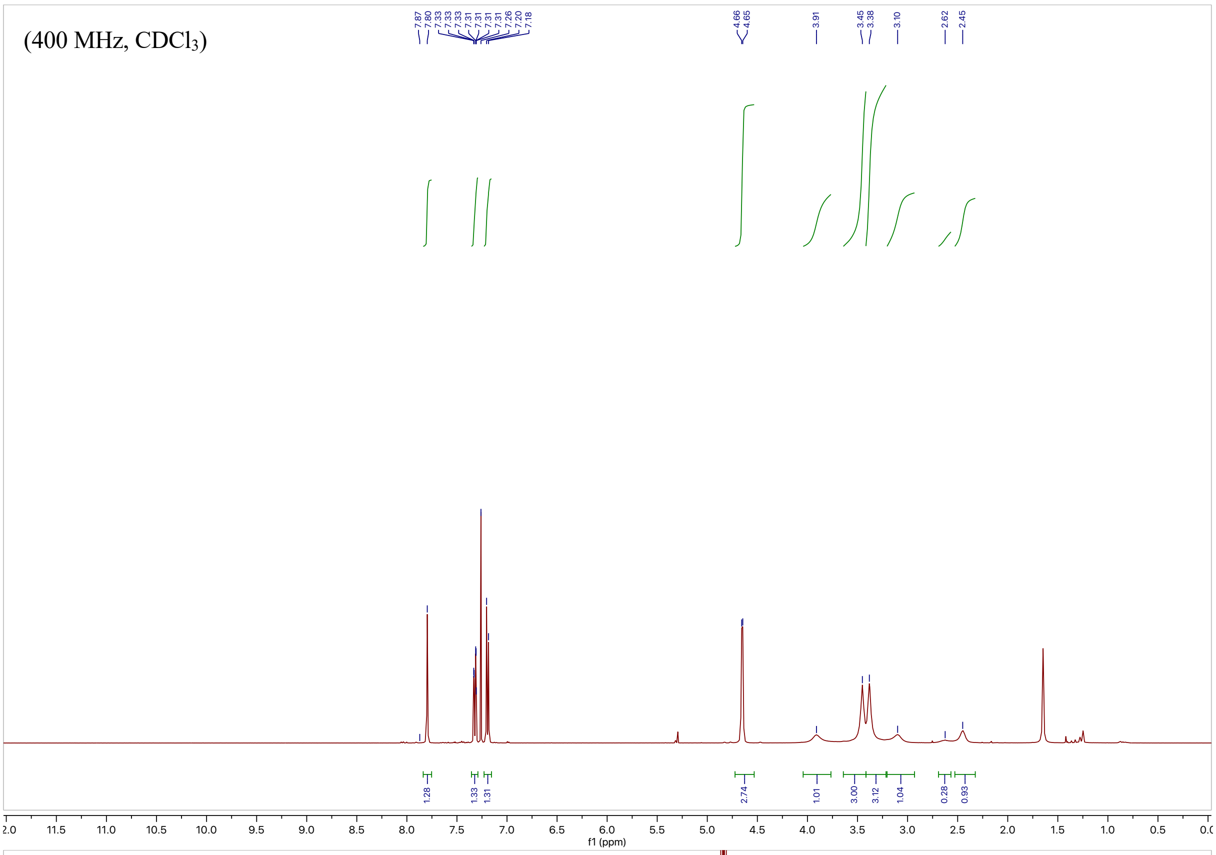

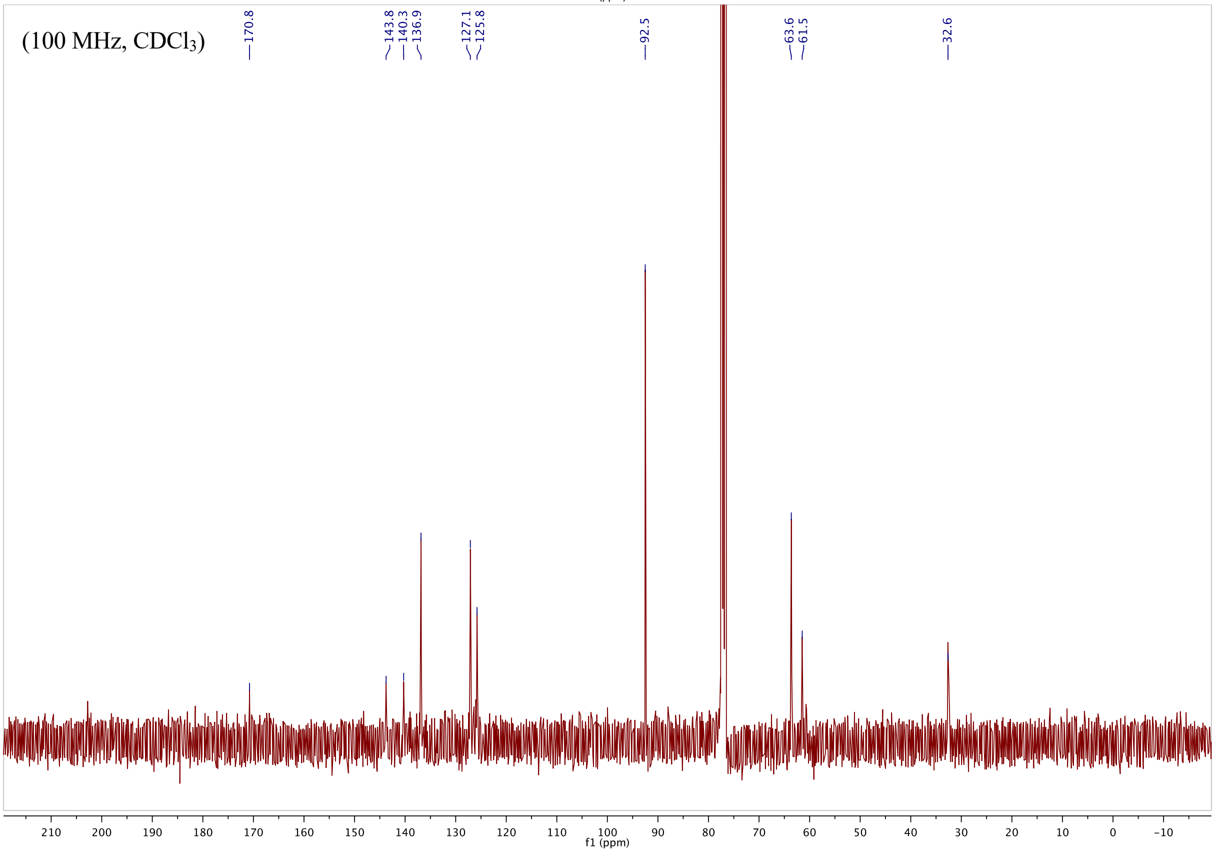

4-(2-Chloroethyl)-2-iodo-*N*-methoxy-*N*-methylbenzamide (5g)

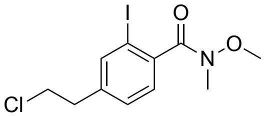

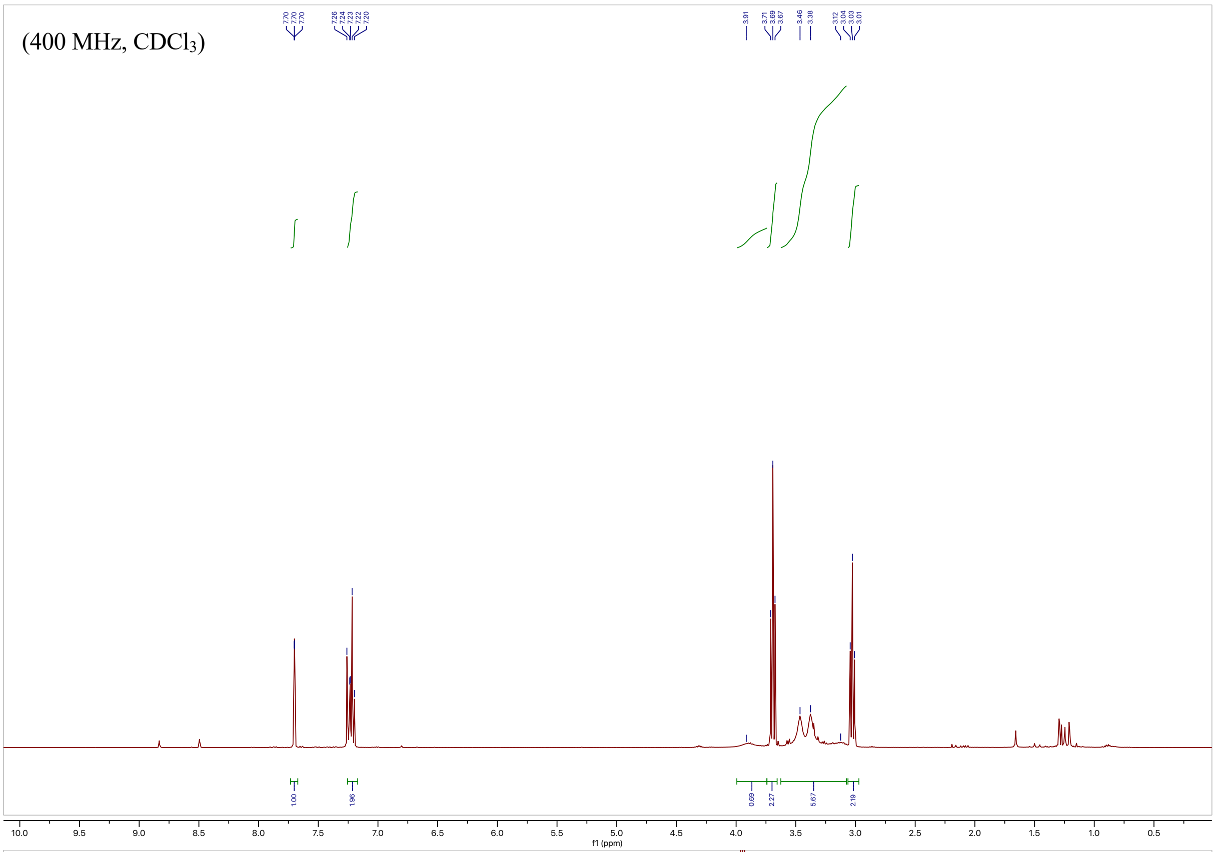

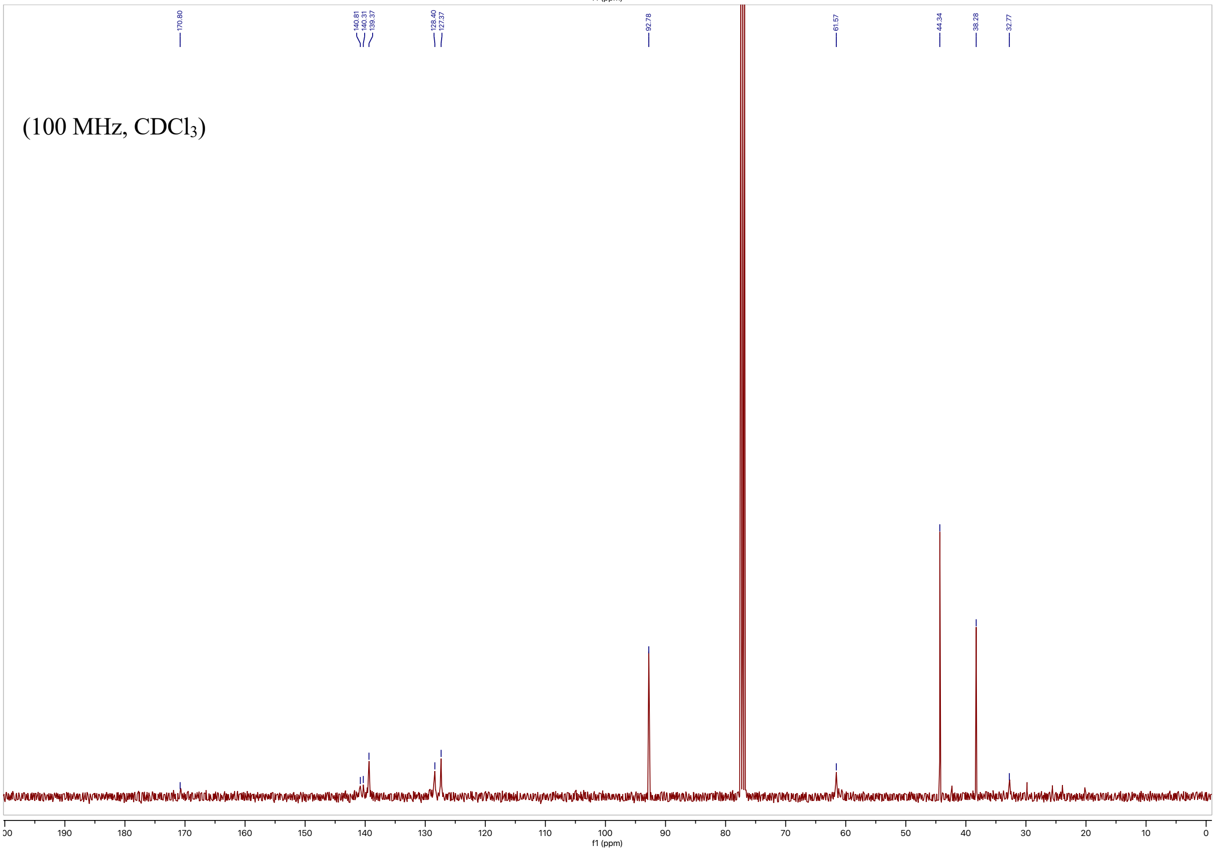

Methyl 3-iodo-4-(methoxy(methyl)carbamoyl)benzoate (5h)

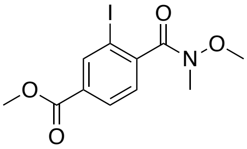

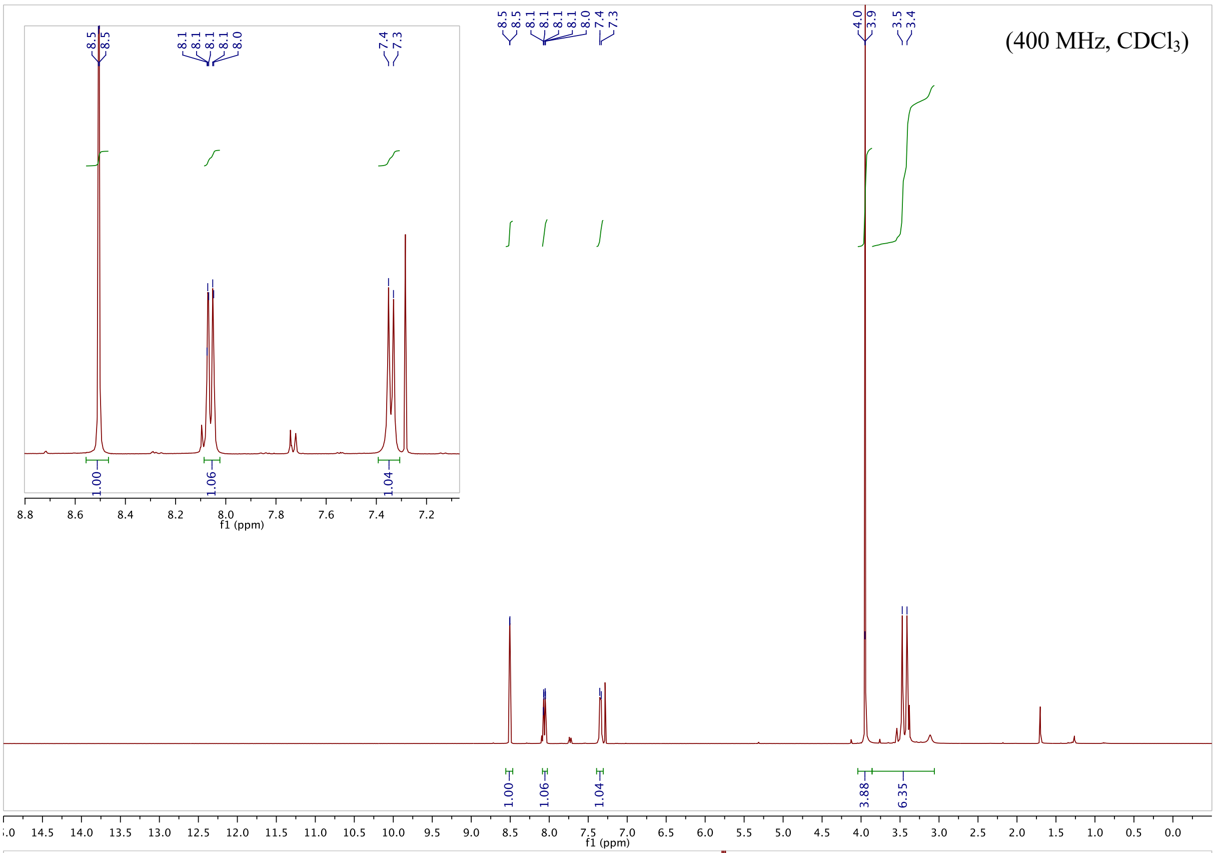

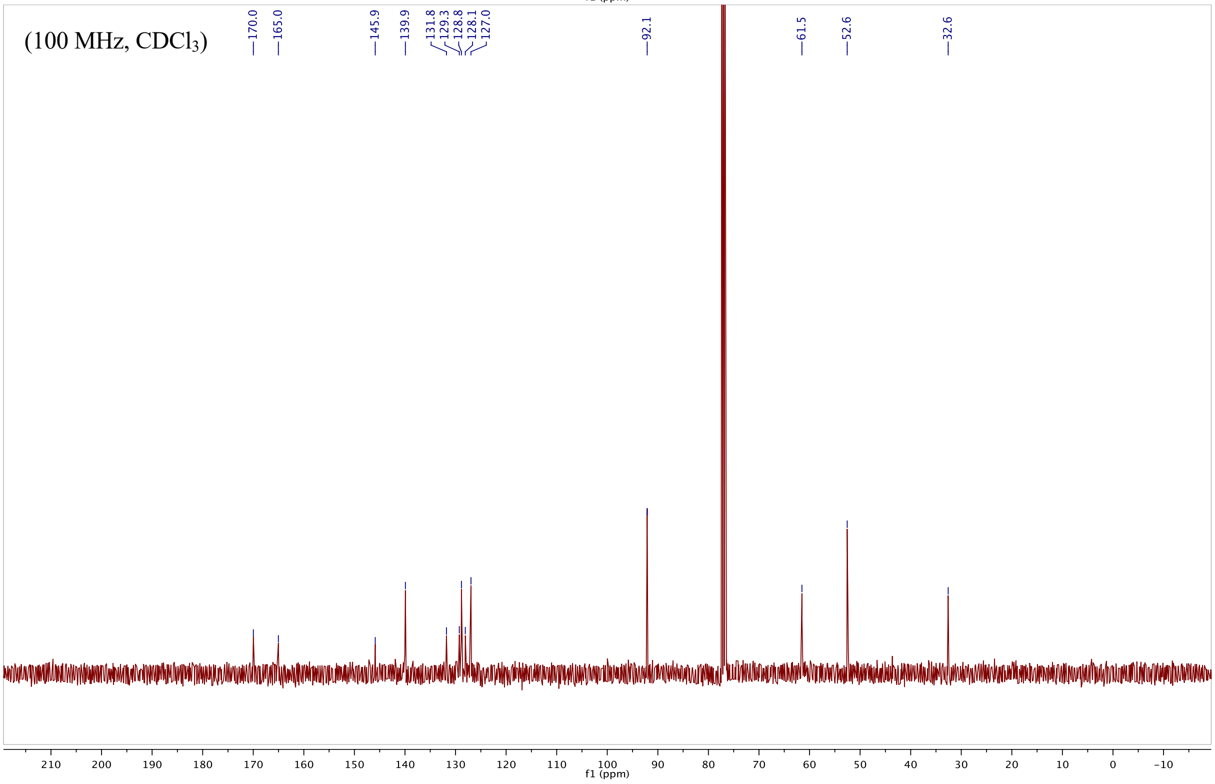

3,5-Difluoro-2-iodo-*N*-methoxy-*N*-methylbenzamide (5i)

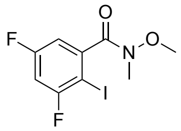

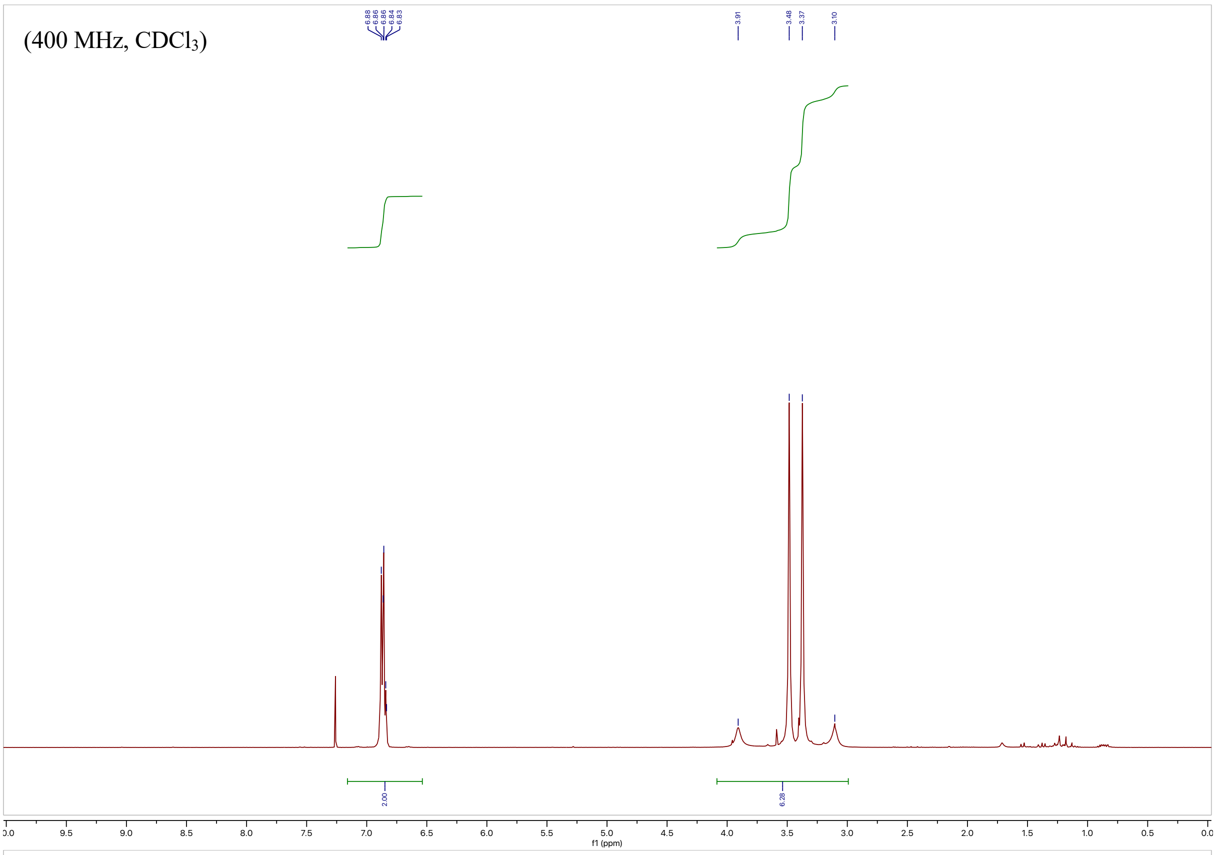

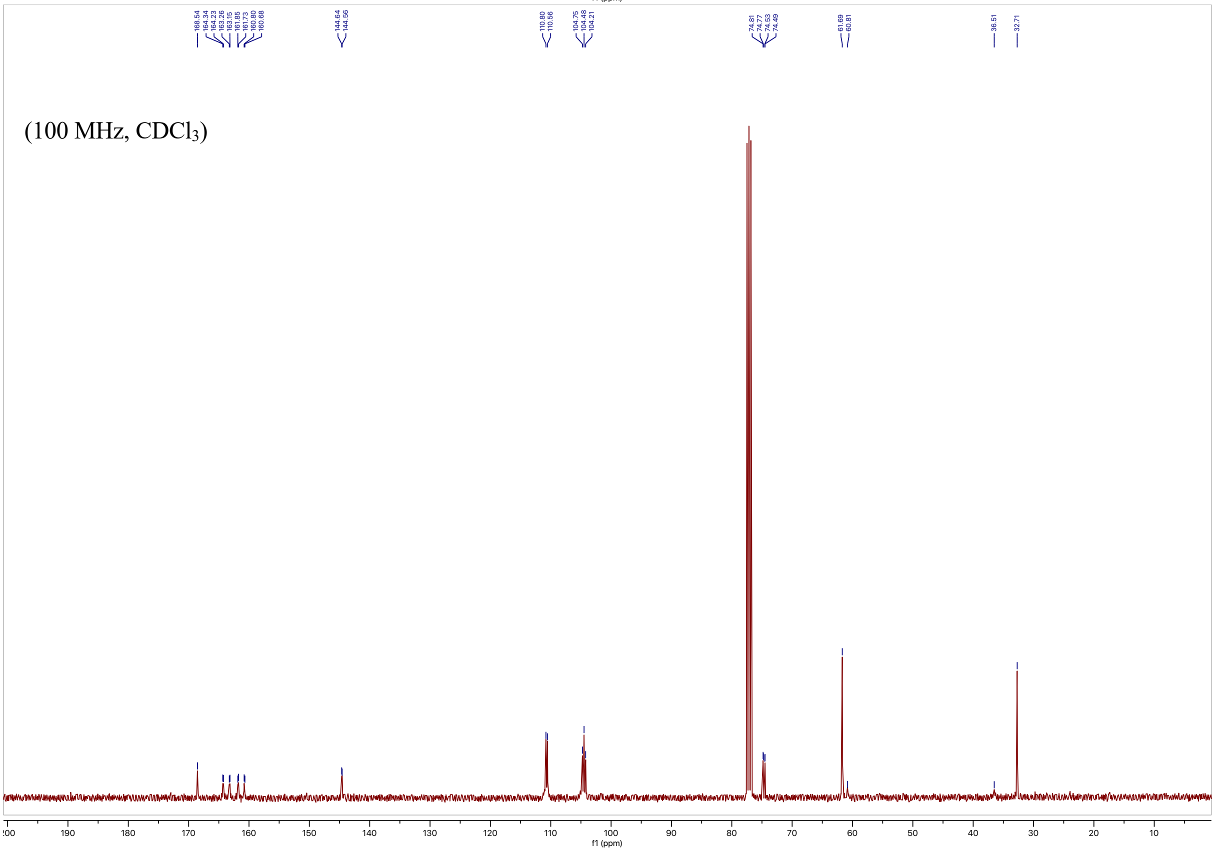

4-Iodo-N-methoxy-N-methylbenzo[d][1,3]dioxole-5-carboxamide (5j)

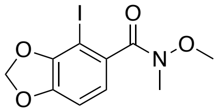

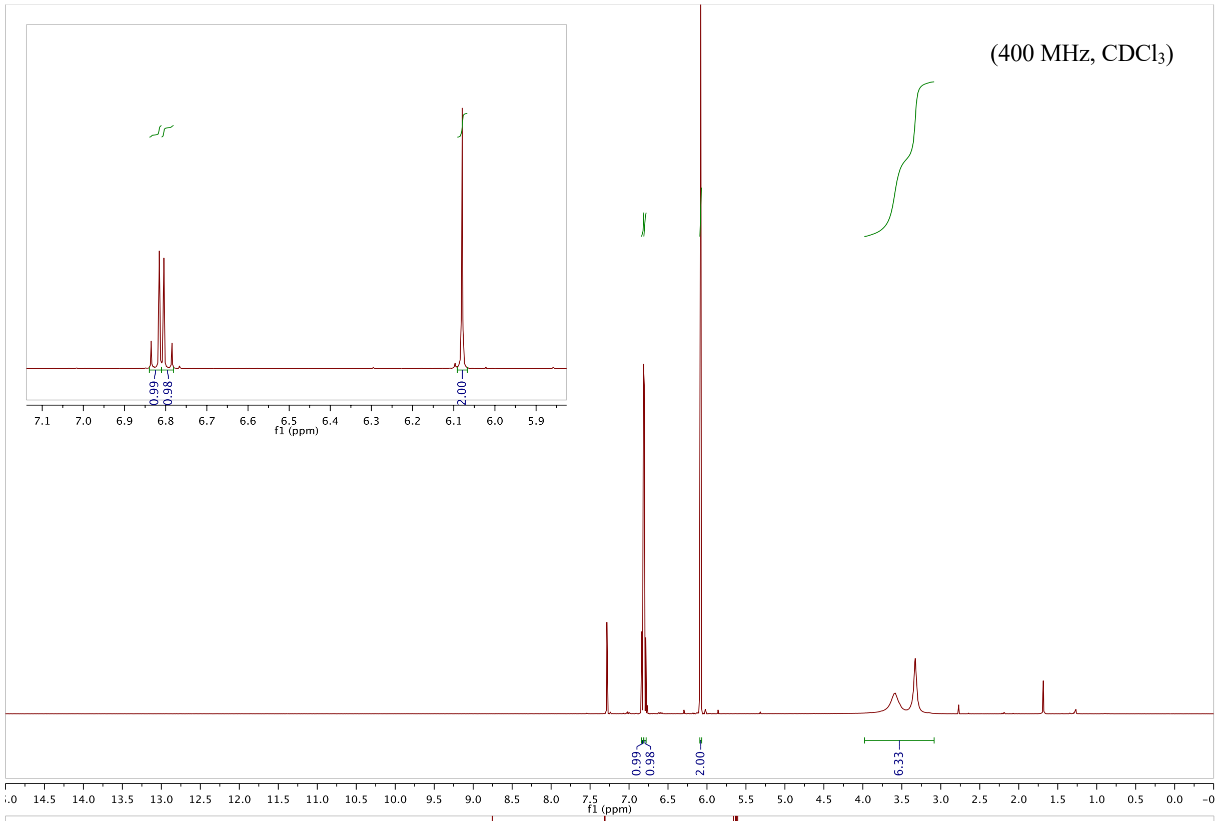

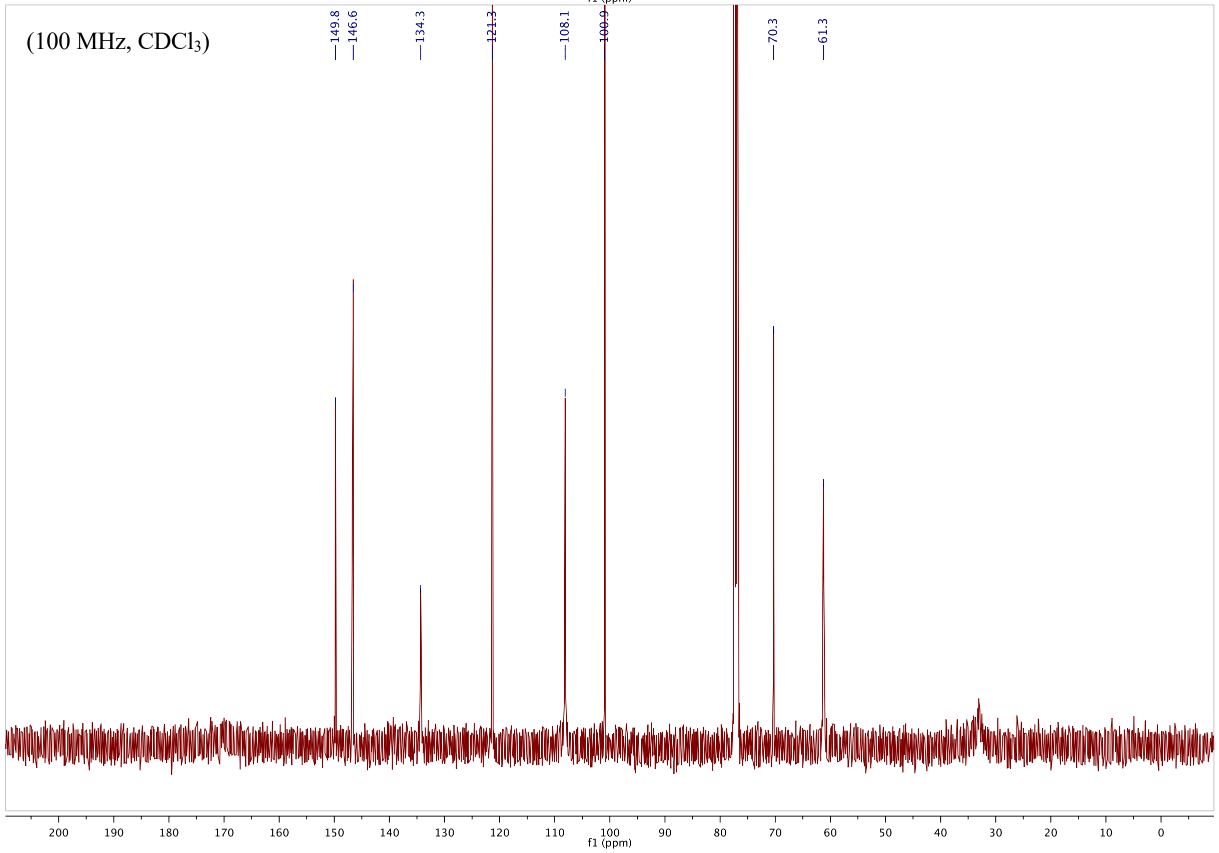

3-Iodo-*N*-methoxy-*N*-methylfuran-2-carboxamide (5k)

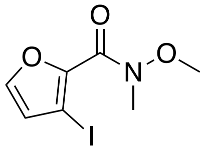

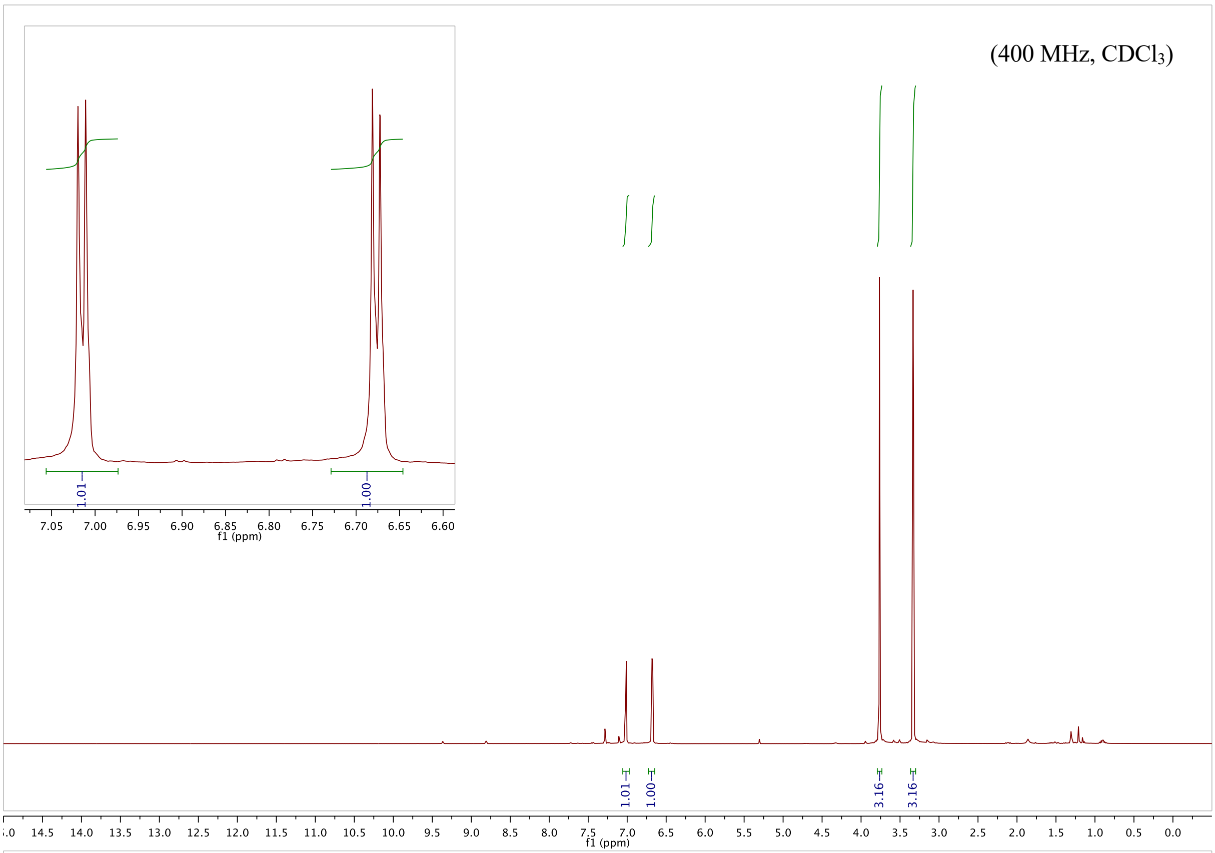

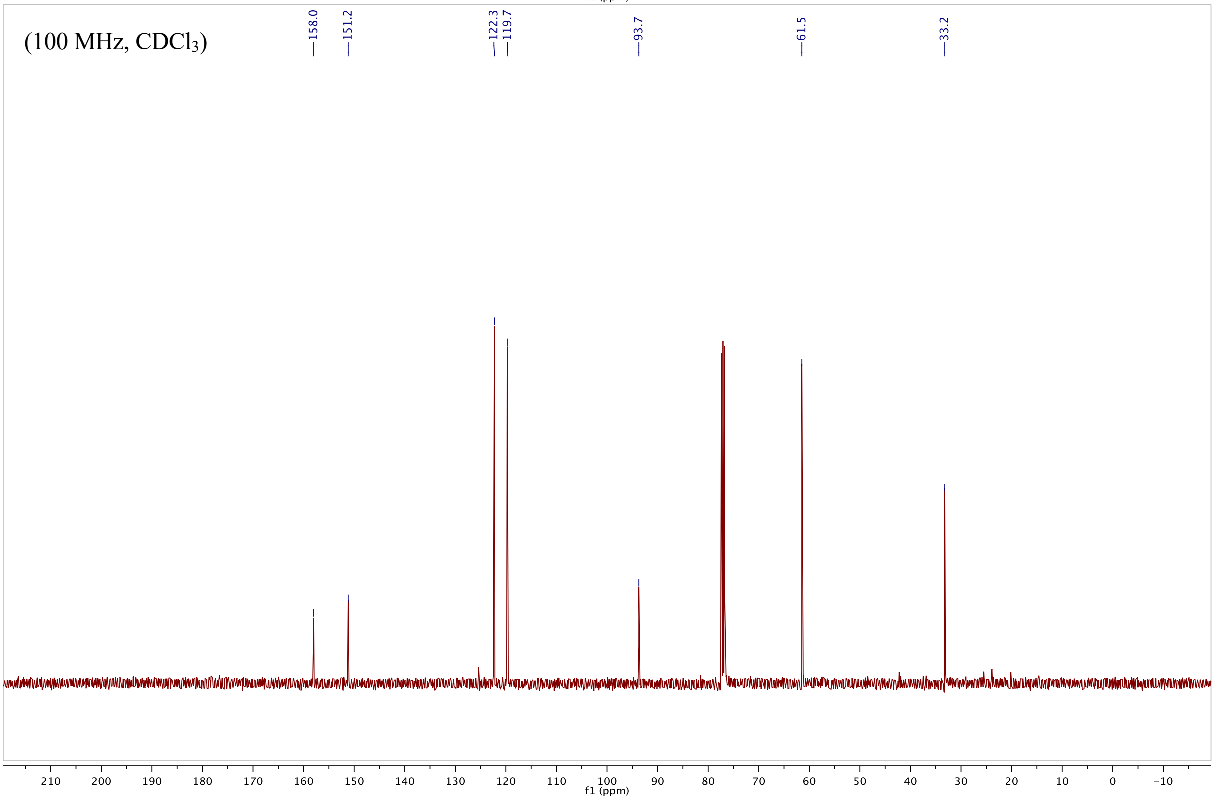

2-Iodo-*N*-methoxy-*N*,5-dimethylbenzamide + 2-iodo-*N*-methoxy-*N*,3-dimethylbenzamide (5l + 6l)

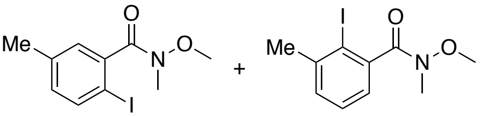

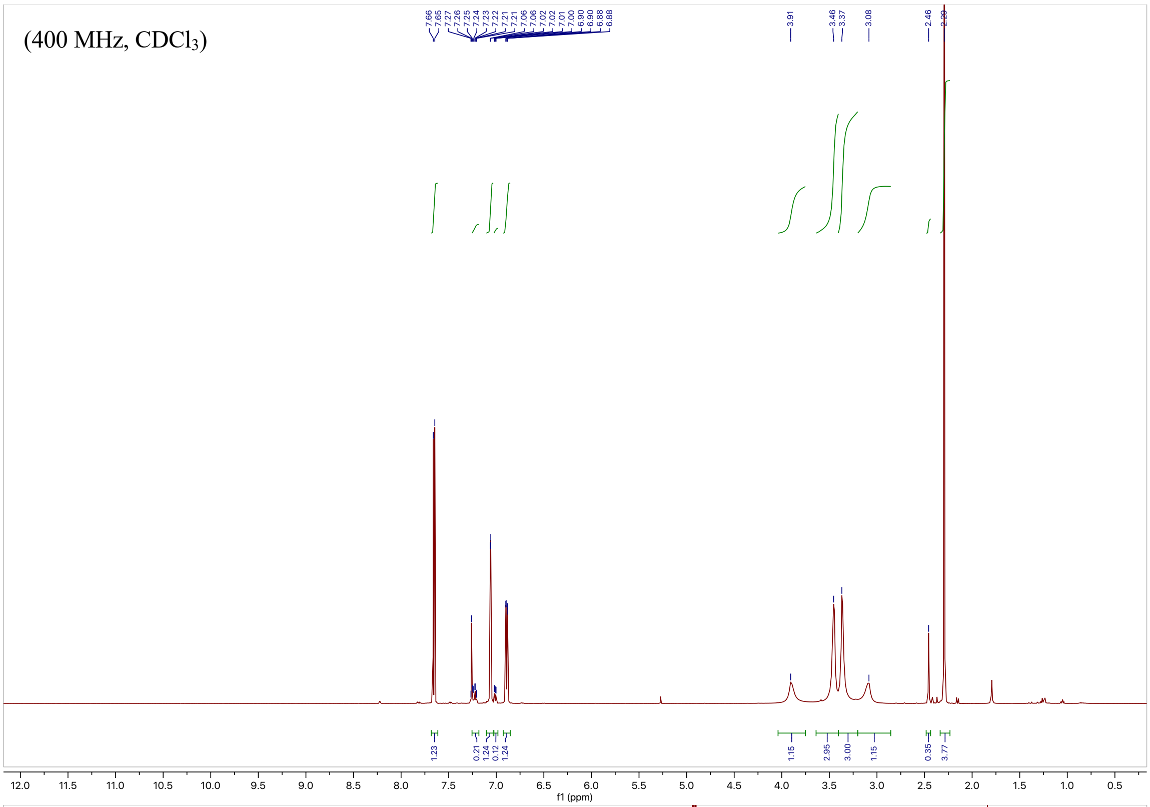

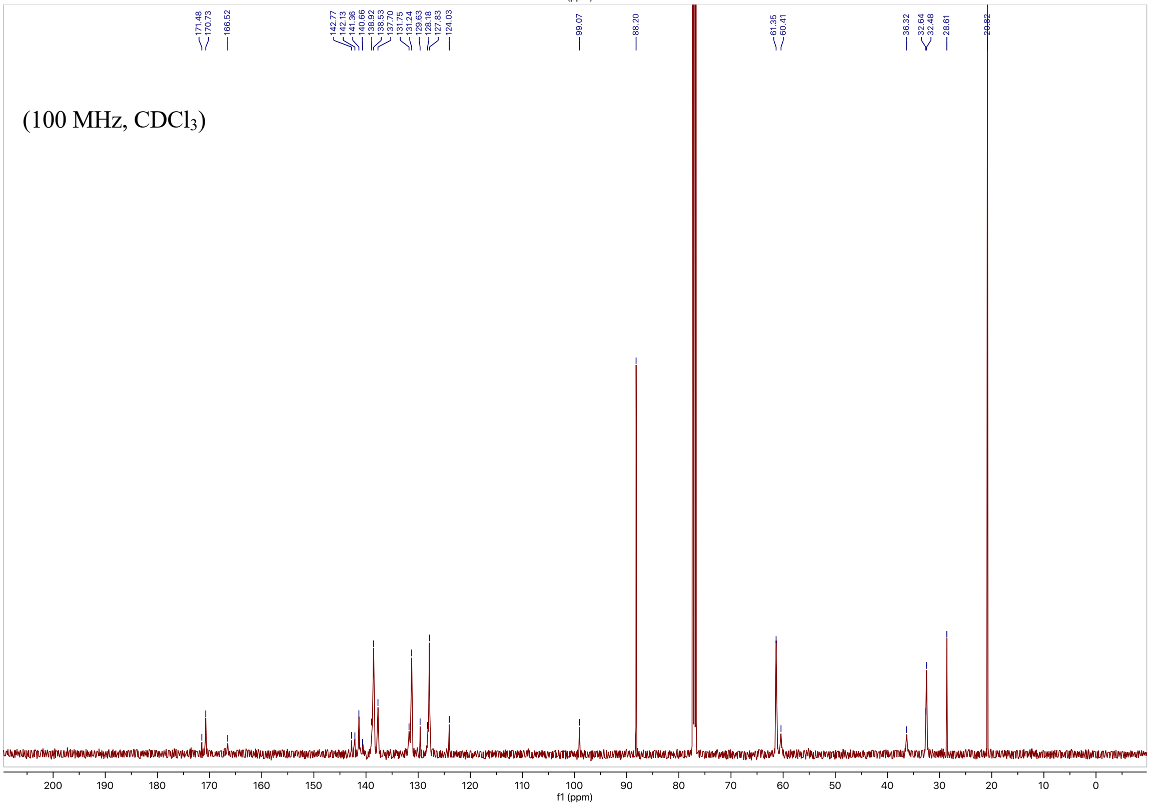

5-Bromo-2-iodo-*N*-methoxy-*N*-methylbenzamide (5m)

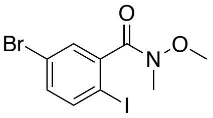

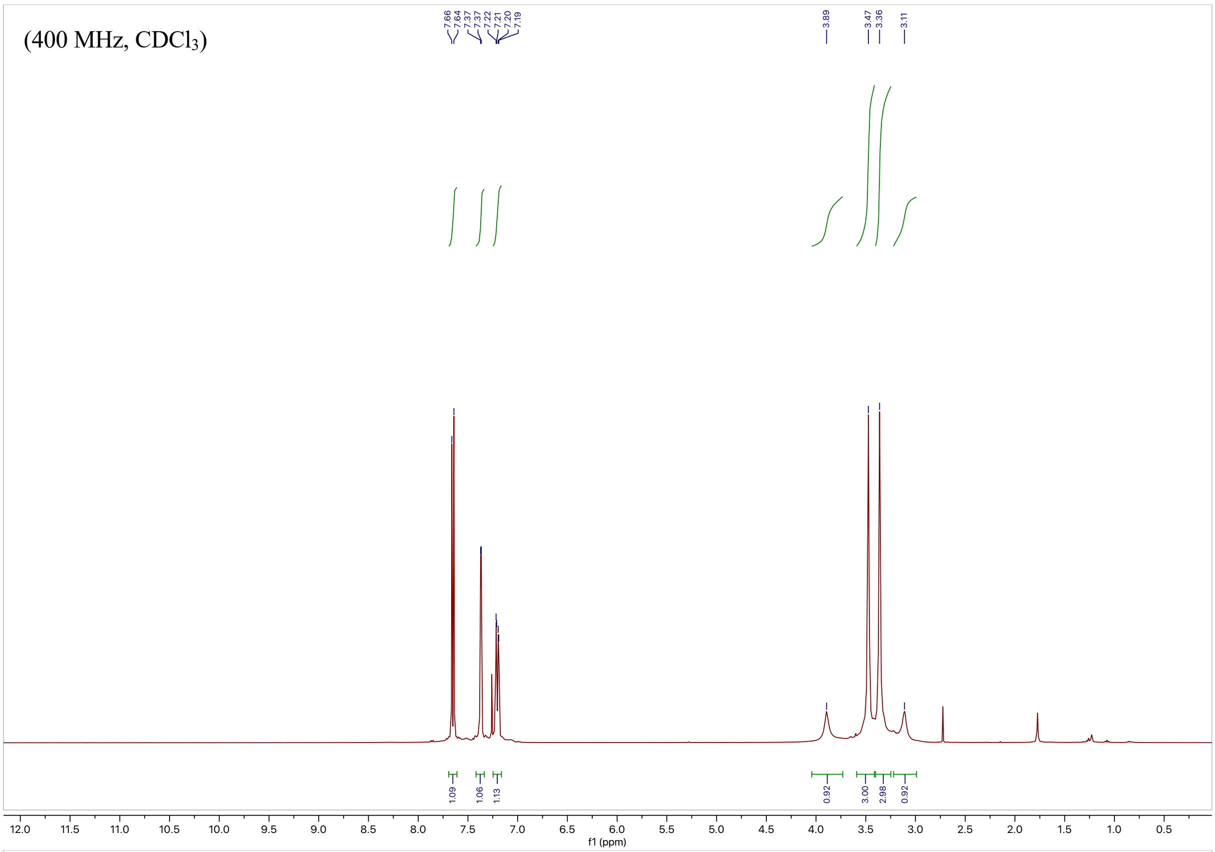

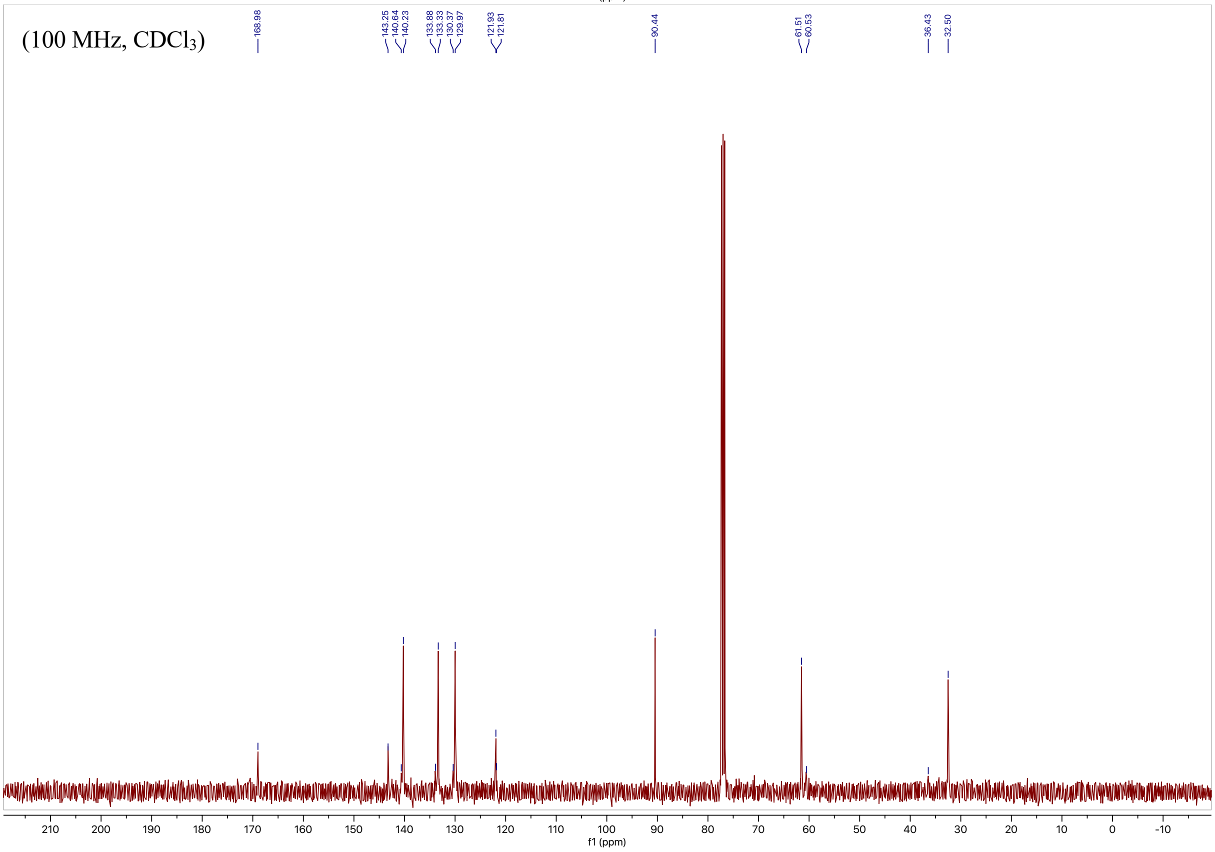

3-Bromo-2-iodo-*N*-methoxy-*N*-methylbenzamide (6m)

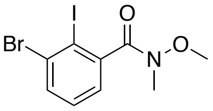

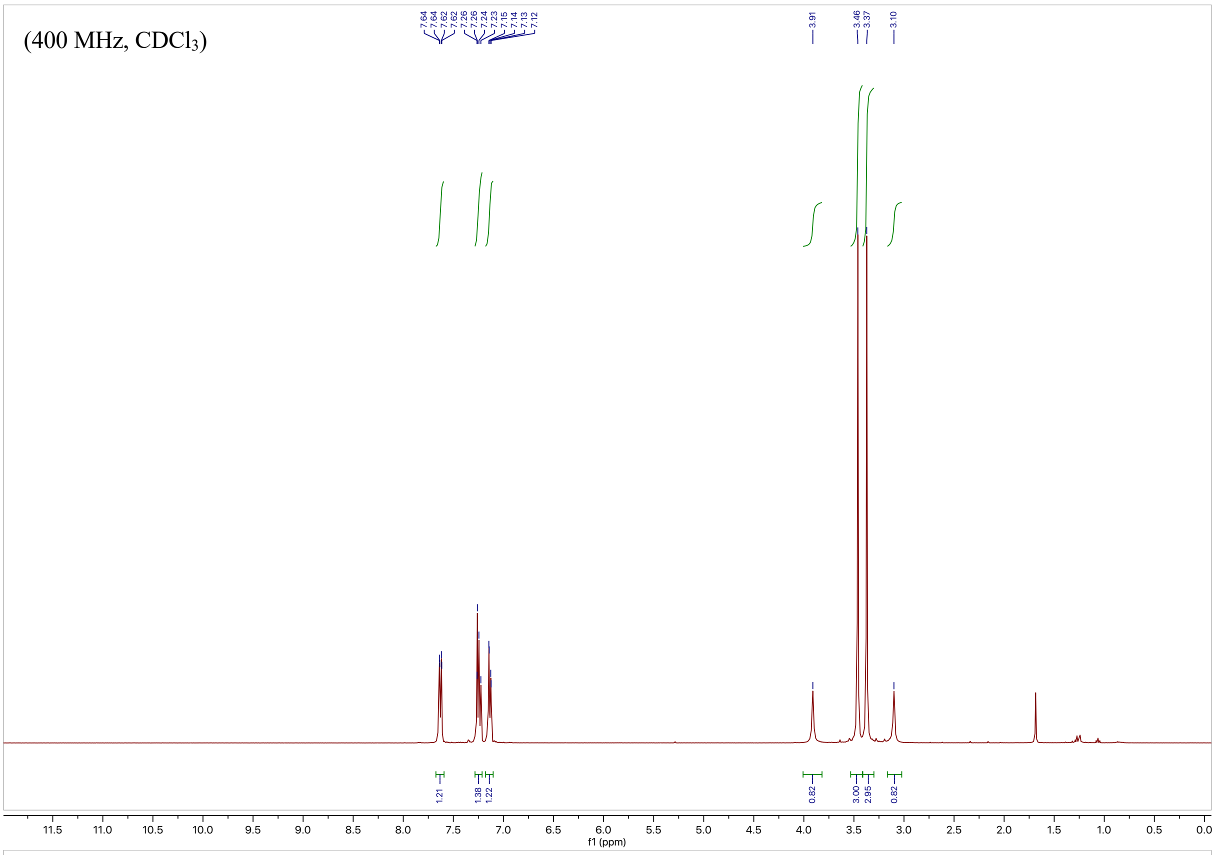

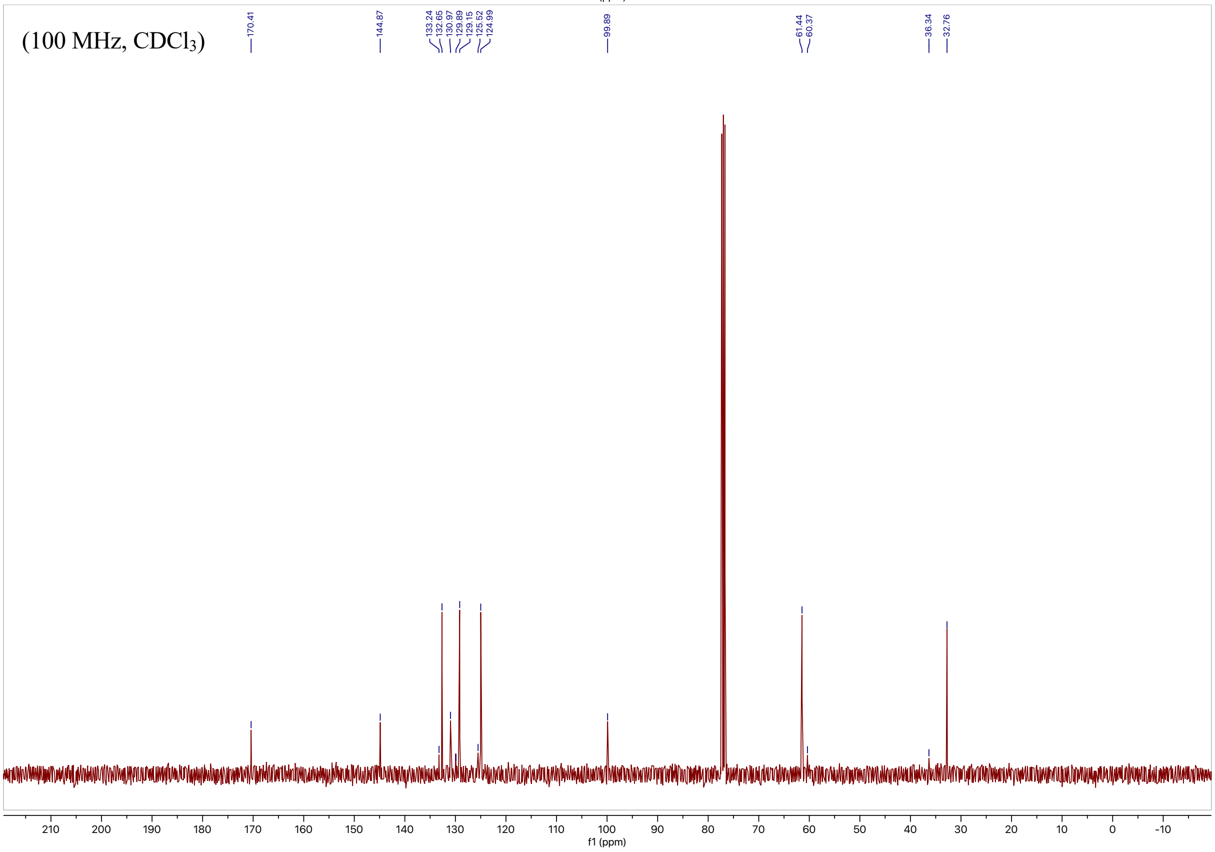

5-Fluoro-2-iodo-*N*-methoxy-*N*-methylbenzamide (5n)

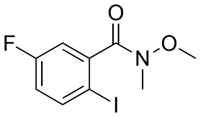

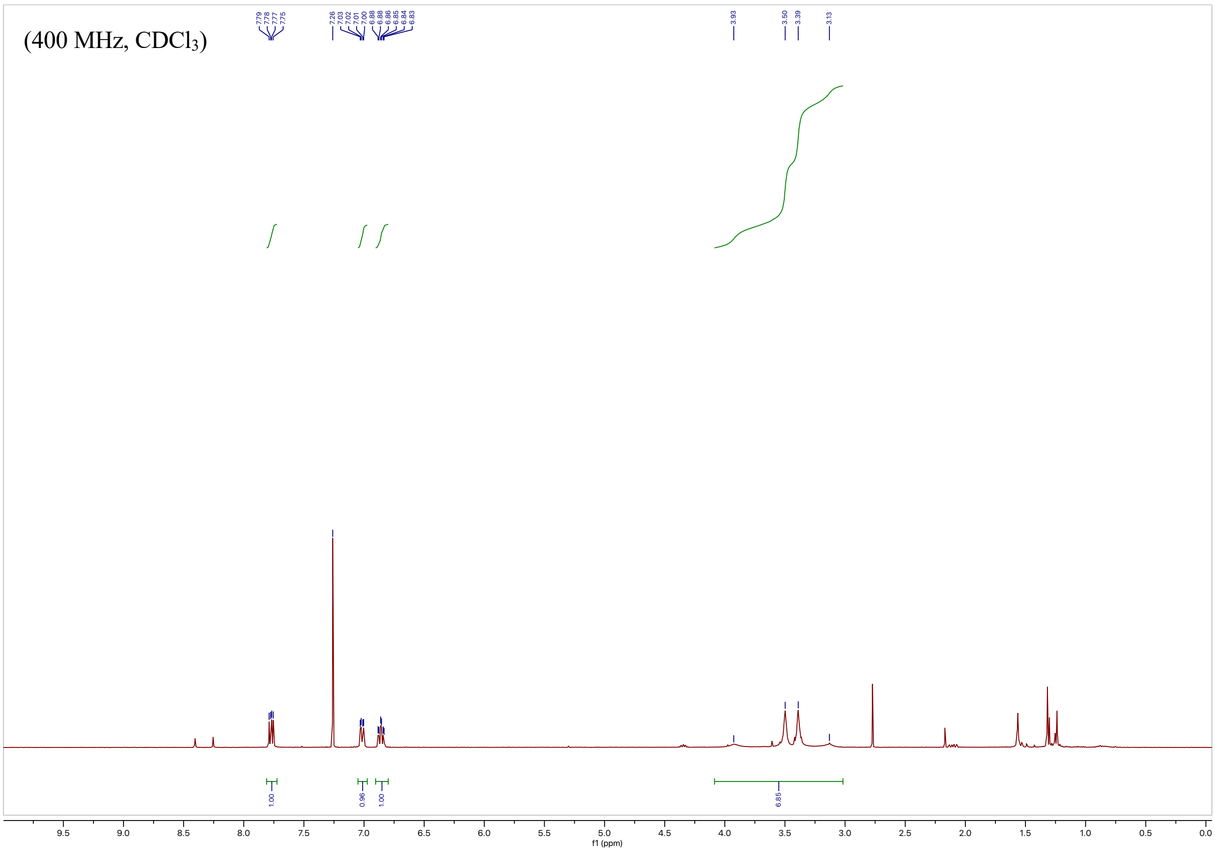

3-Fluoro-2-iodo-*N*-methoxy-*N*-methylbenzamide (6n)

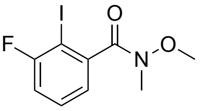

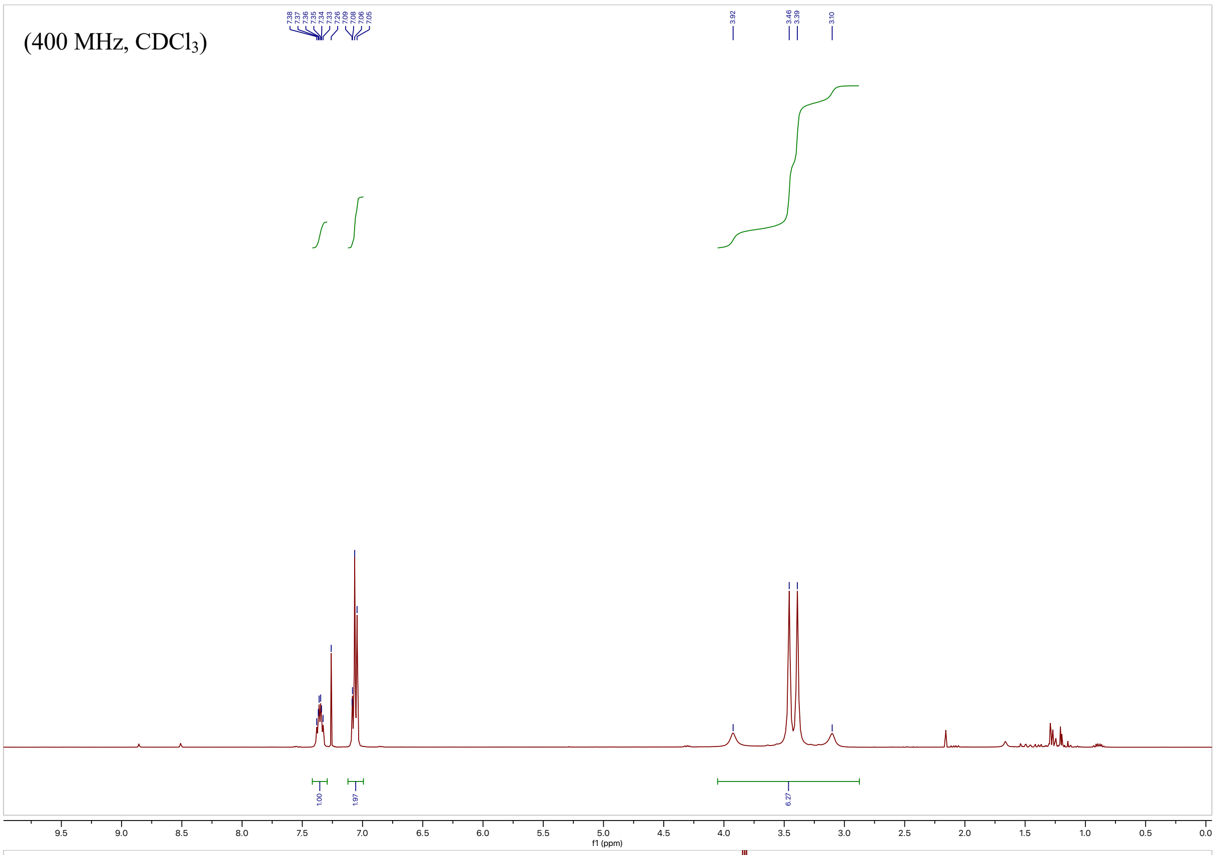

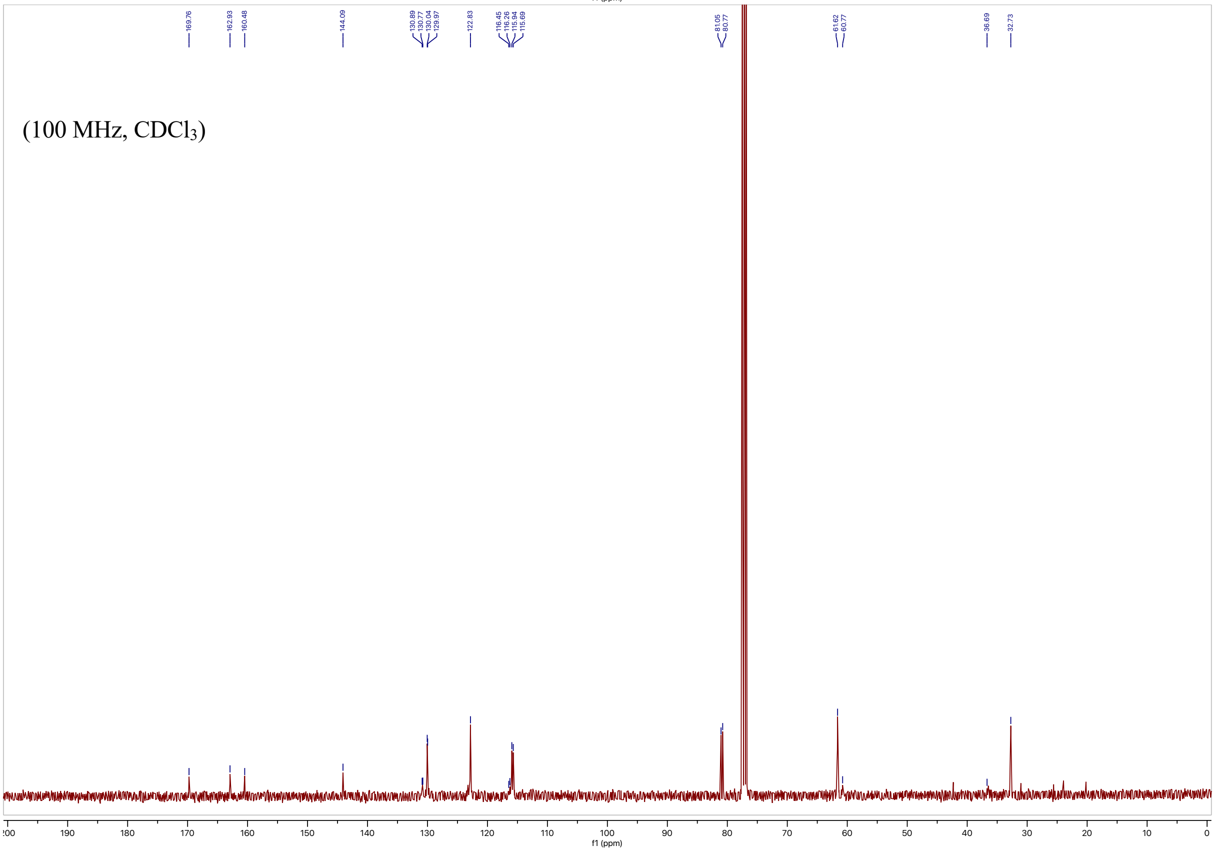

2-Iodo-*N*-methoxy-*N*-methyl-5-nitrobenzamide (5o)

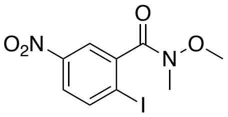

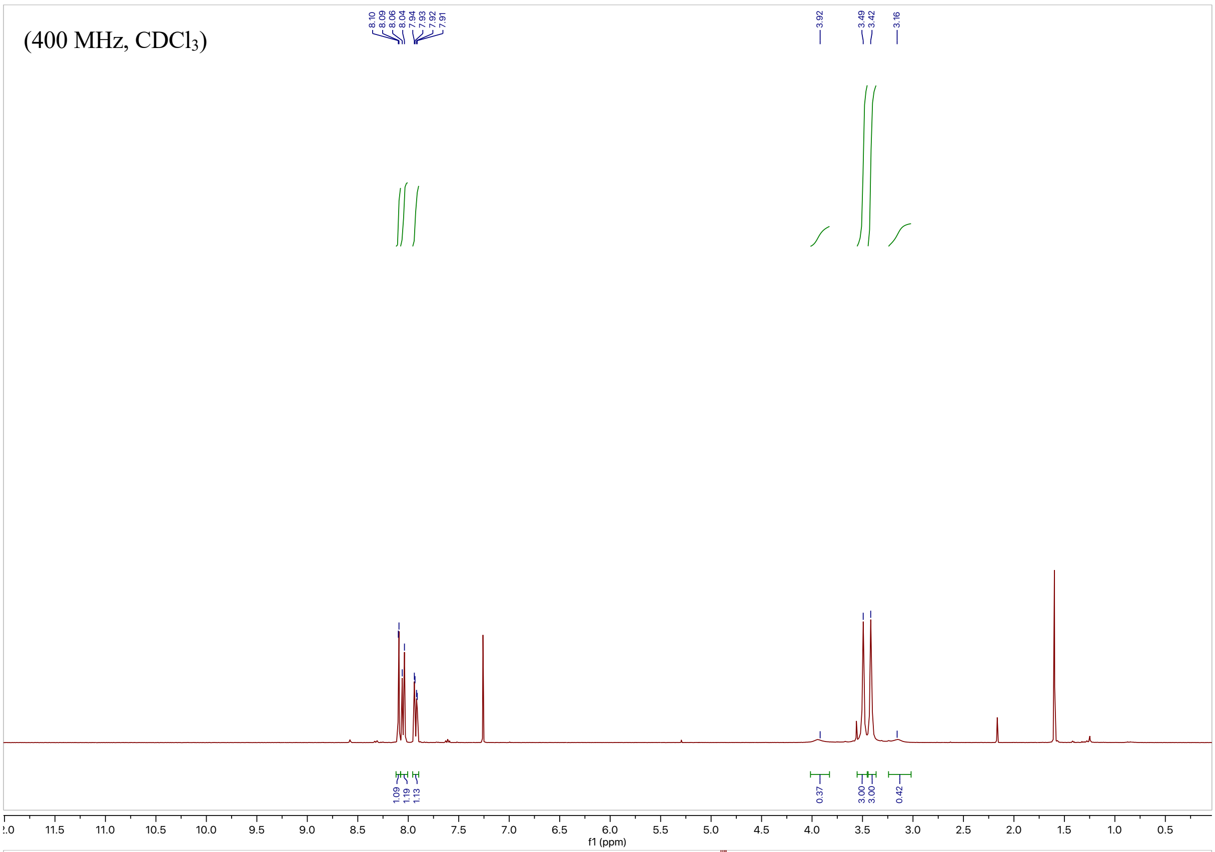

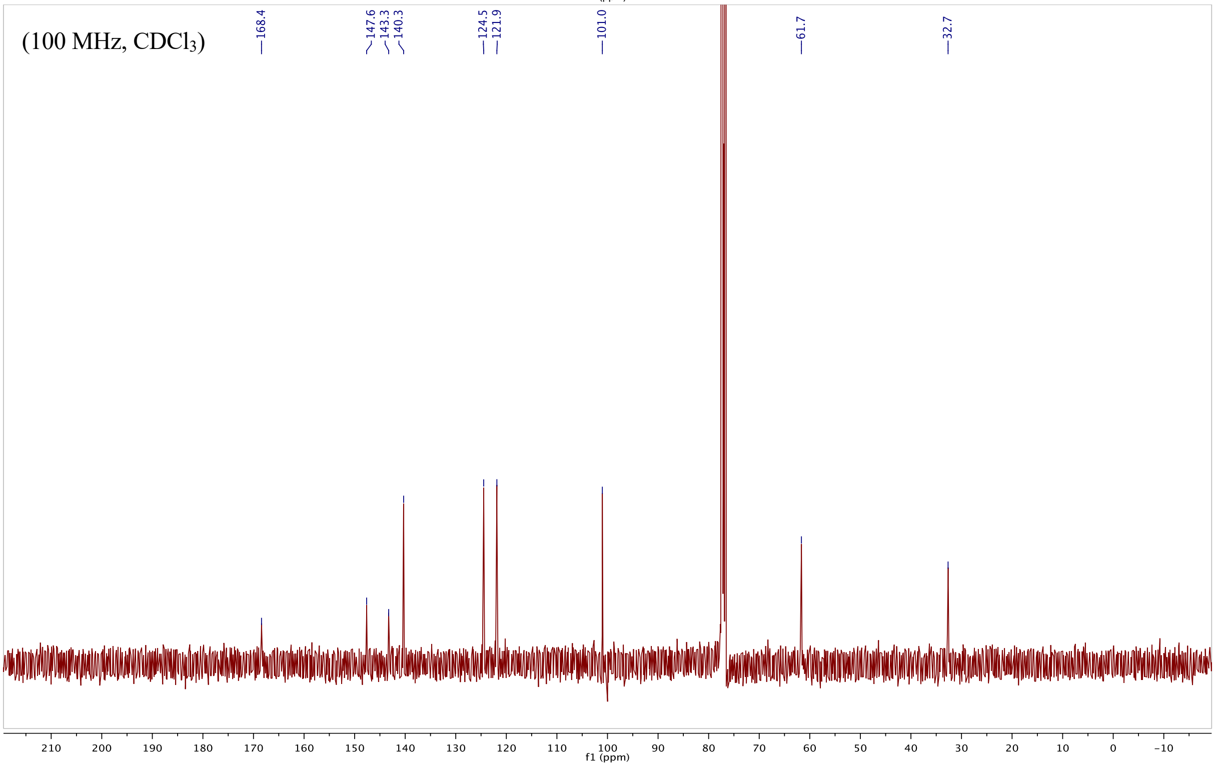

2-Iodo-N-methoxy-N-methyl-3-nitrobenzamide (6o)

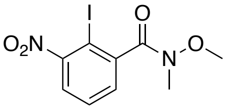

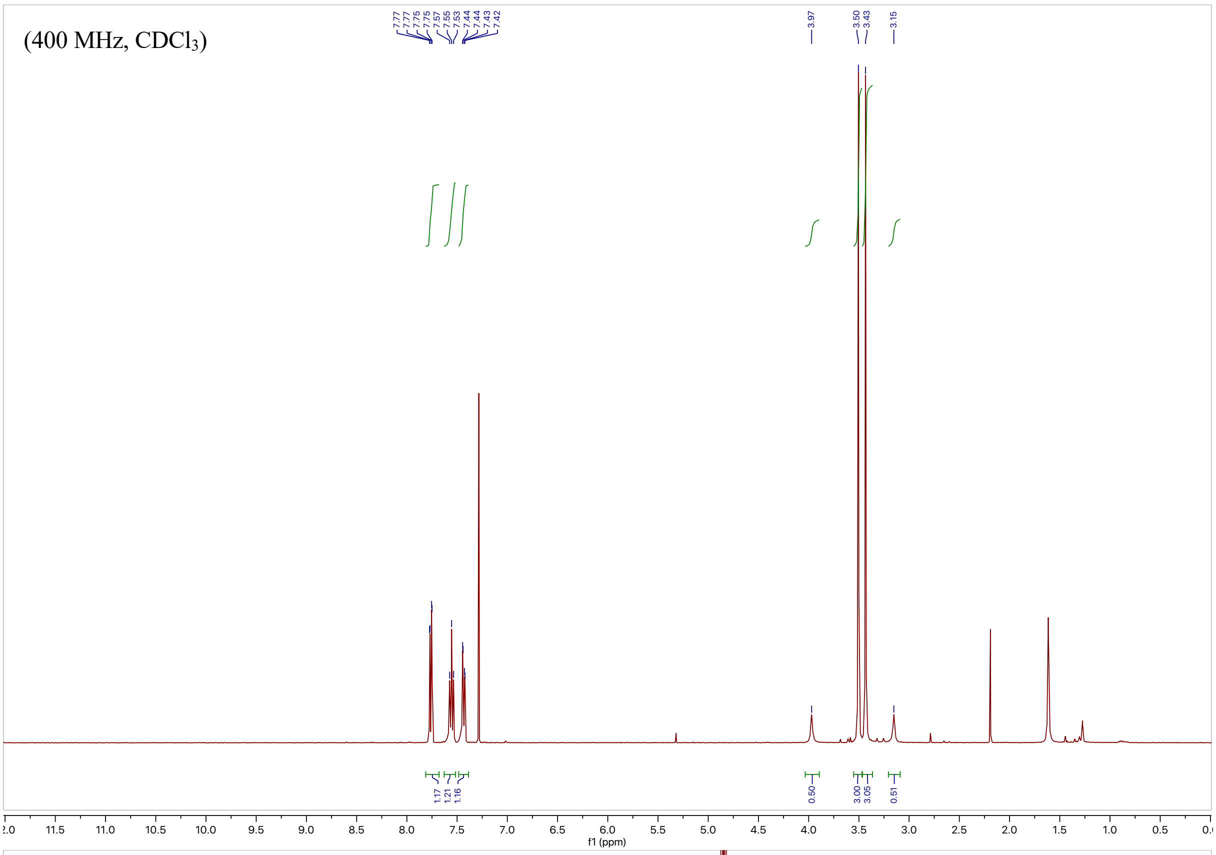

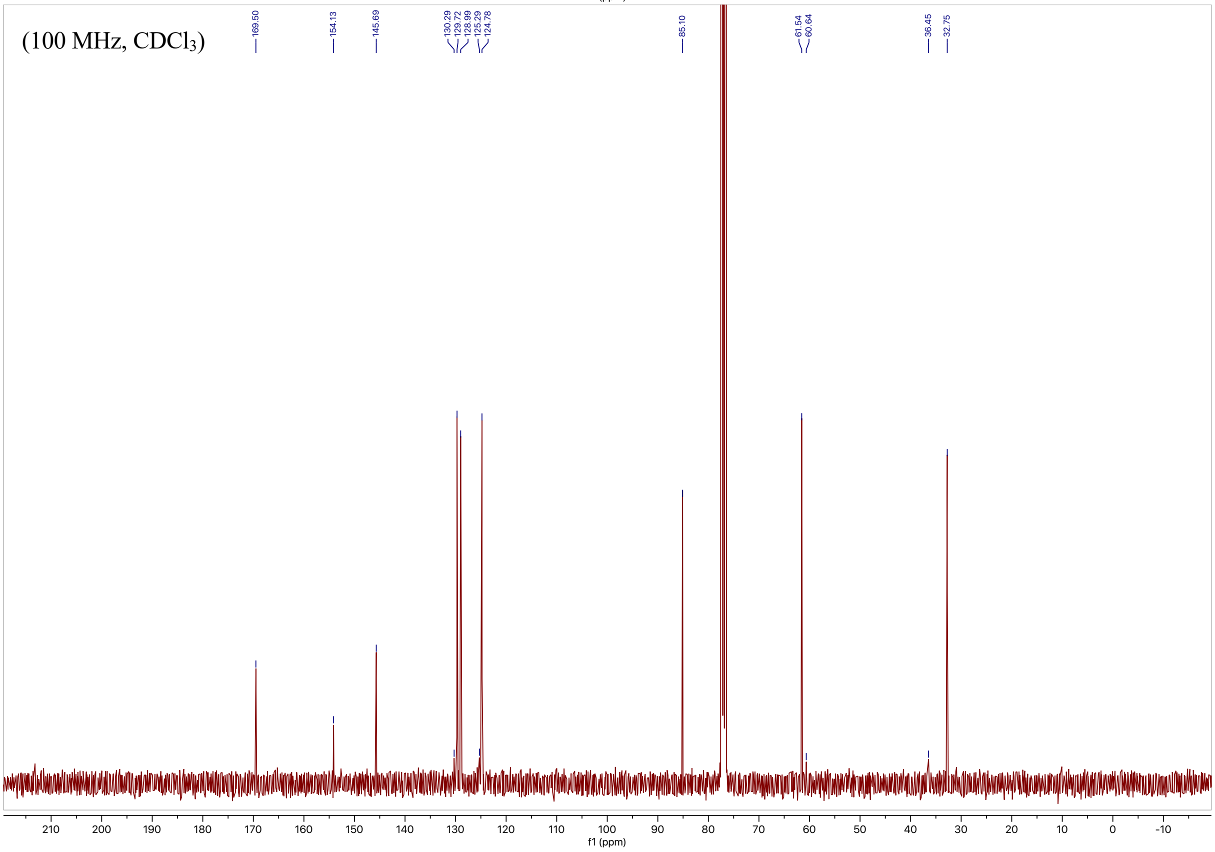

^{1}H NMR, ^{13}C NMR of deuterated benzamides 1a-d_2 and 4a-d_2

***N*-(*tert*-Butyl)benzamide-2,6-d_2 (1a-d_2)**

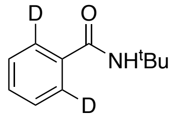

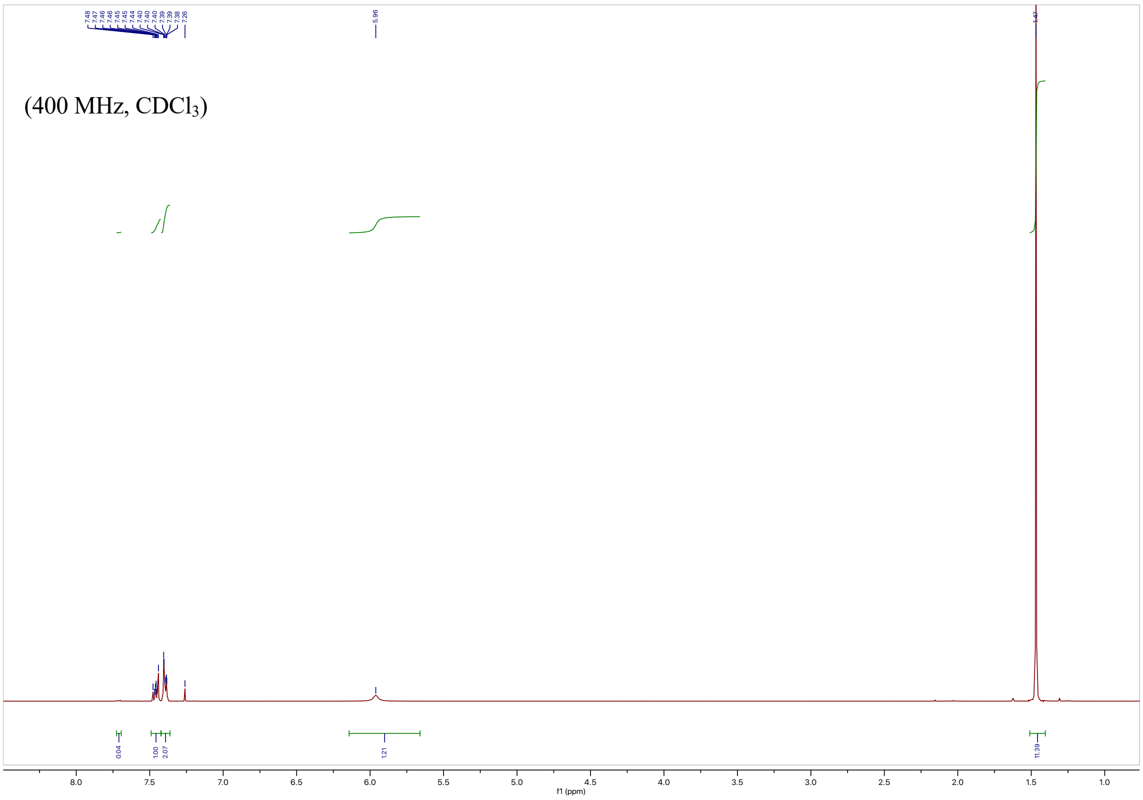

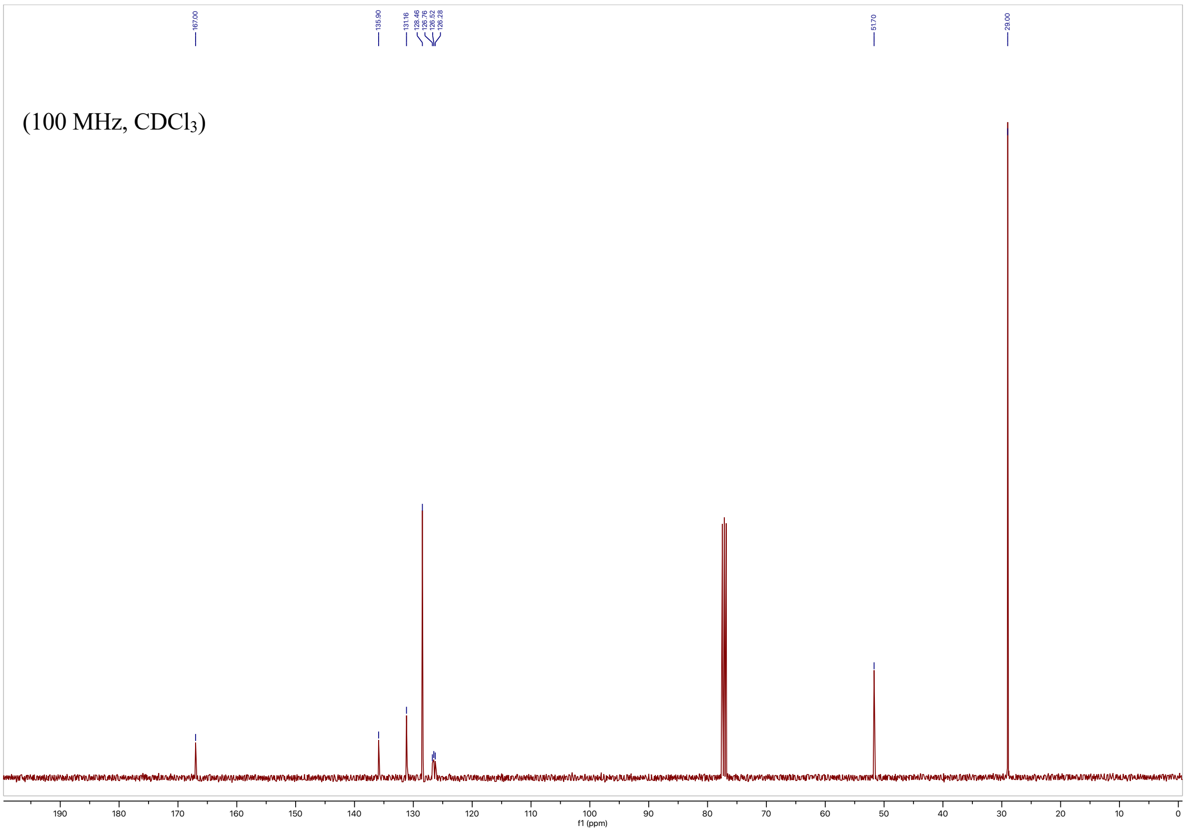

N-Methoxy-*N*-methylbenzamide-2,6-d_2 (4a-d_2)

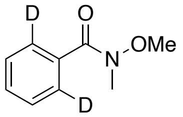

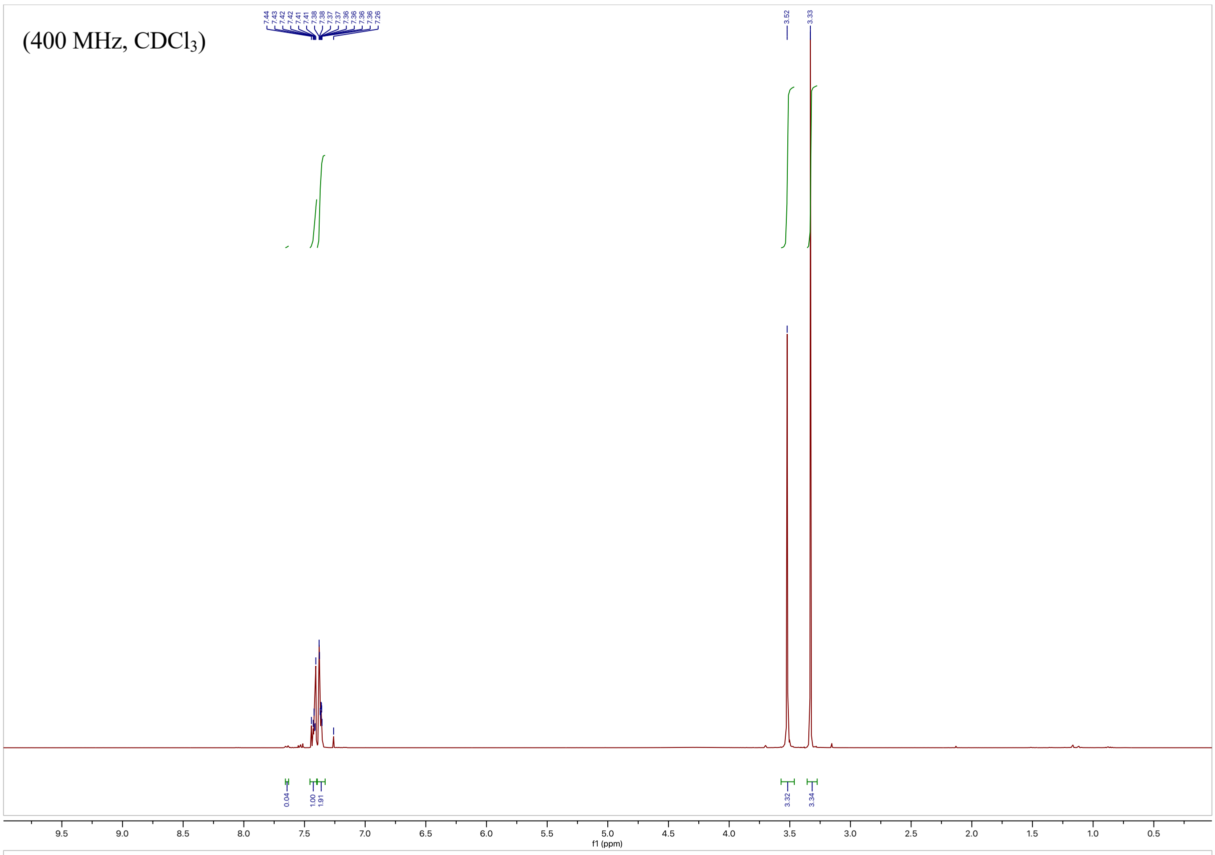

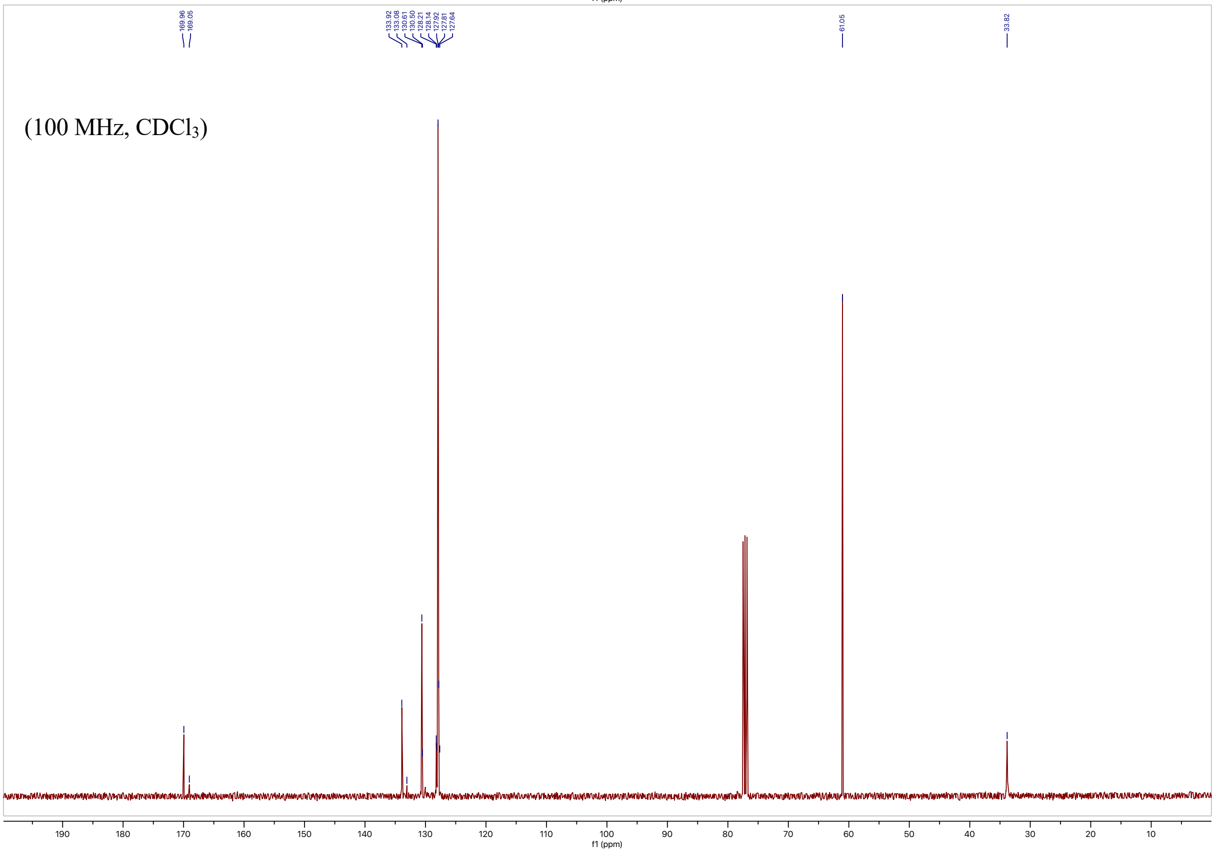

^{1}H NMR, ^{13}C NMR of deuterated *ortho*-iodo-benzamides 2a-*d* and 5a-*d*

N-(*tert*-Butyl)-2-iodobenzamide-6-*d* (2a-*d*)

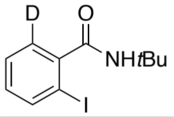

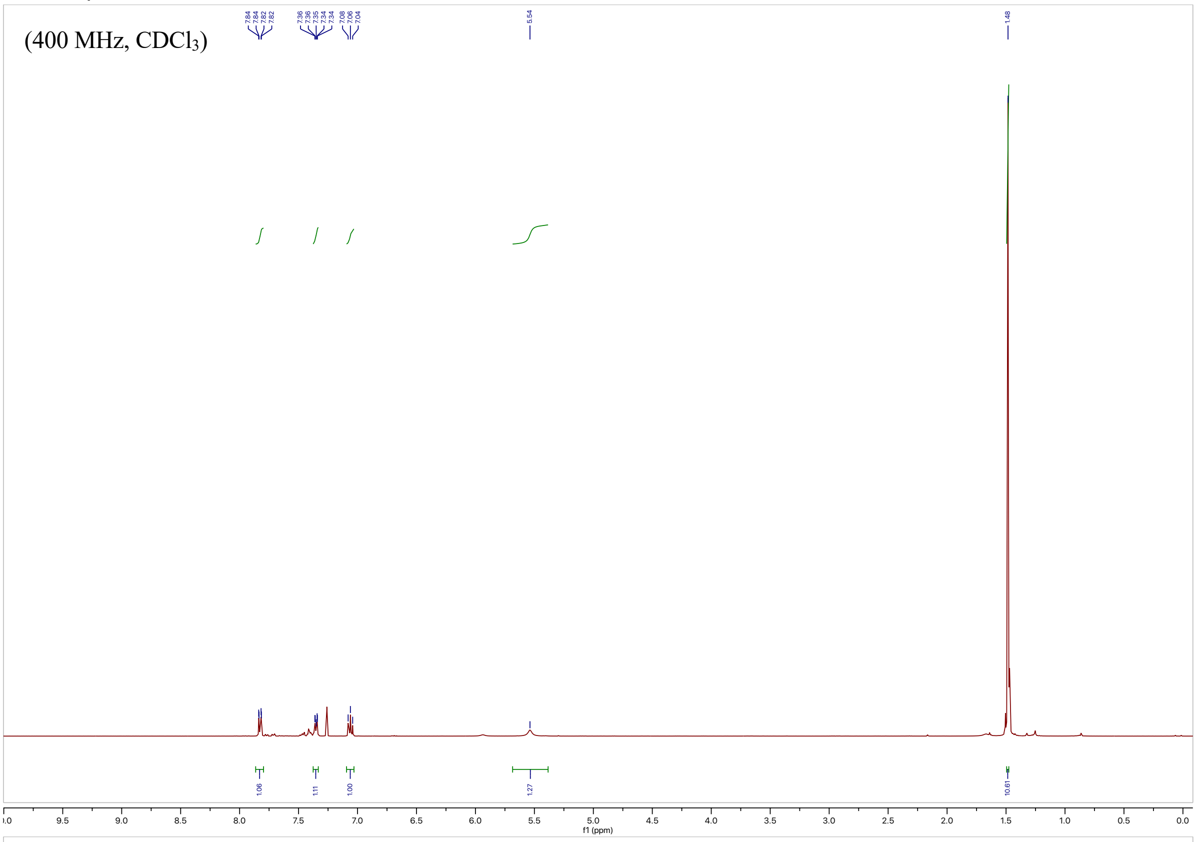

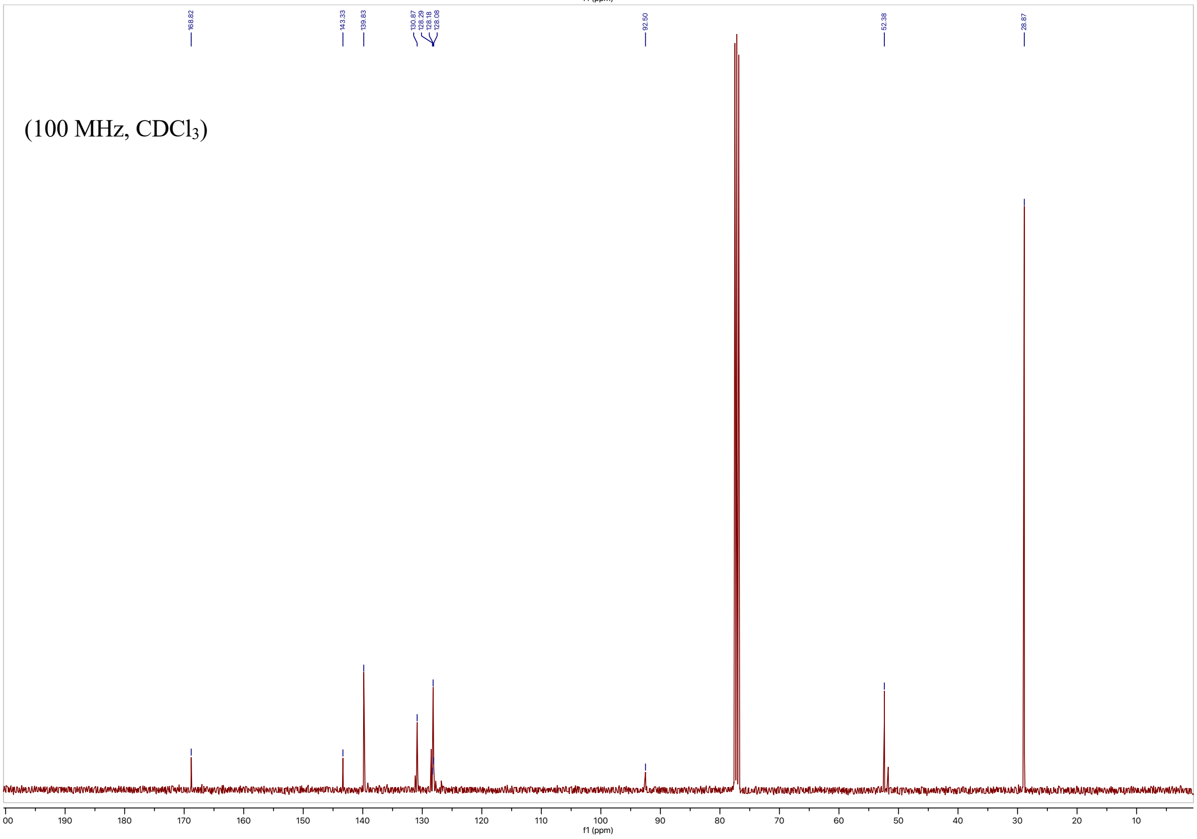

2-Iodo-*N*-methoxy-*N*-methylbenzamide-6-*d* (5a-*d*)

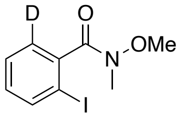

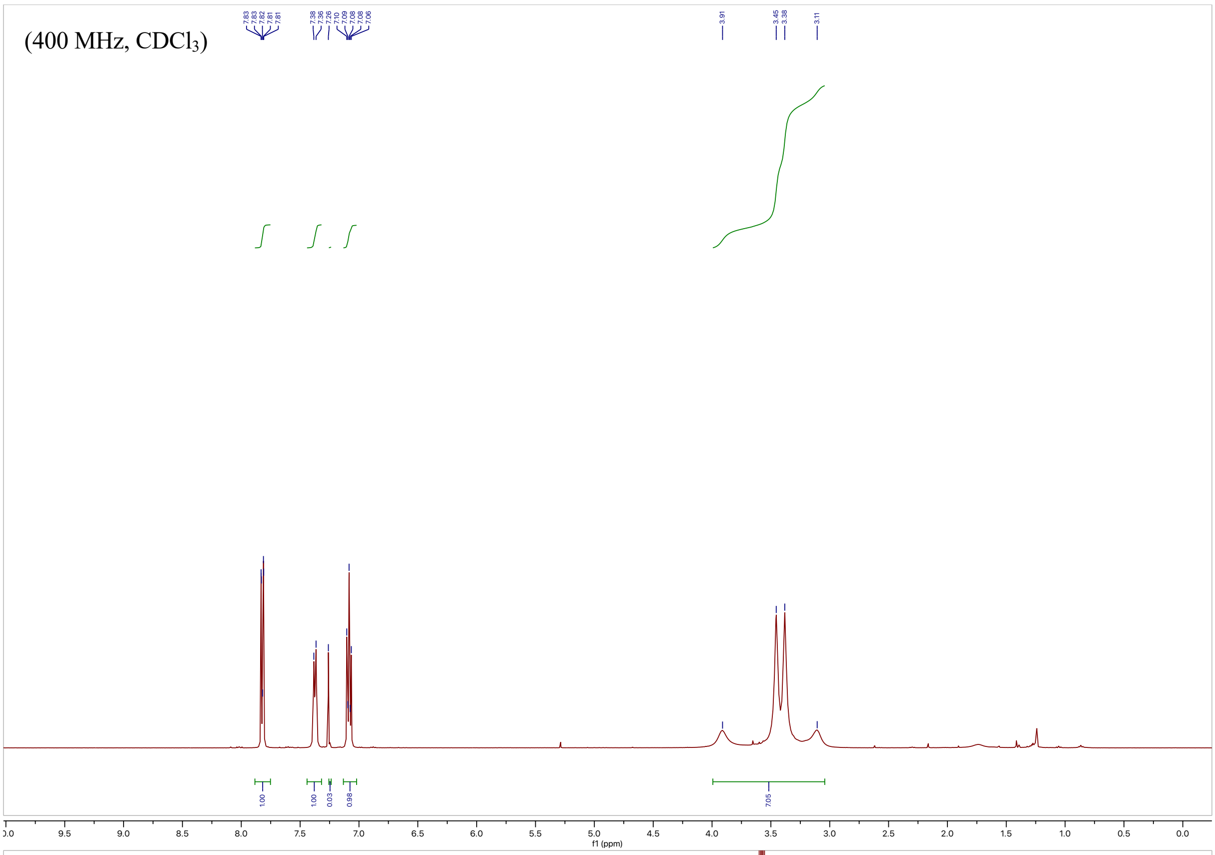

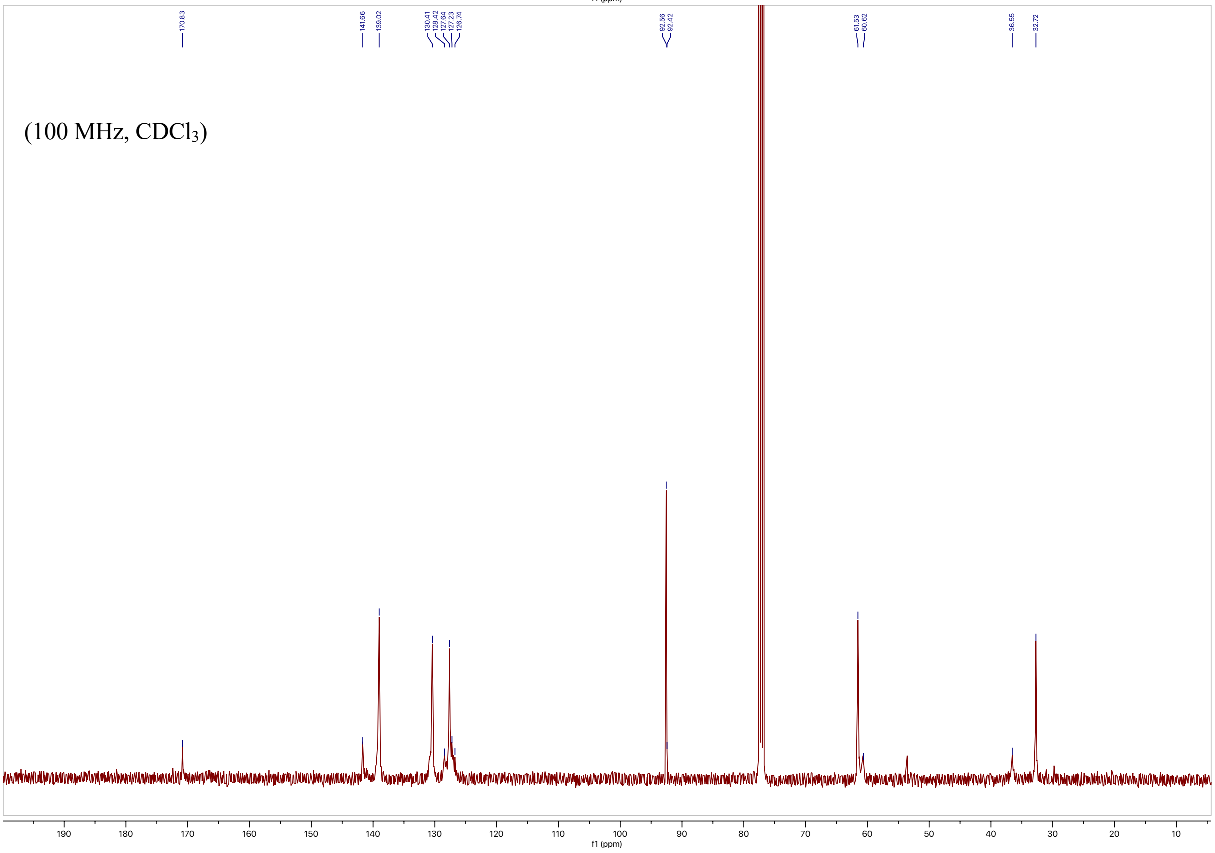

Supplement: Supplementary file 1 — ol3c03190_si_001.pdf [file ol3c03190_si_001.pdf]
